# Causal associations between both psoriasis and psoriatic arthritis and multiple autoimmune diseases: a bidirectional two-sample Mendelian randomization study

**DOI:** 10.3389/fimmu.2024.1422626

**Published:** 2024-07-25

**Authors:** Kexin Duan, Jingrui Wang, Shaomin Chen, Tong Chen, Jiajue Wang, Shujing Wang, Xinsheng Chen

**Affiliations:** ^1^ The Second Clinical Medical College, Guangzhou University of Chinese Medicine, Guangzhou, China; ^2^ The First Clinical Medical College, Guangzhou University of Chinese Medicine, Guangzhou, China; ^3^ Department of Dermatology, Guangdong Provincial Hospital of Traditional Chinese Medicine, Guangzhou, China

**Keywords:** psoriasis, psoriatic arthritis, autoimmune diseases, Mendelian randomization analysis, genome-wide association studies, single nucleotide

## Abstract

**Background:**

Numerous observational studies have identified associations between both psoriasis (PsO) and psoriatic arthritis (PsA), and autoimmune diseases (AIDs); however, the causality of these associations remains undetermined.

**Methods:**

We conducted a bidirectional two-sample Mendelian Randomization study to identify causal associations and directions between both PsO and PsA and AIDs, such as systemic lupus erythematosus (SLE), Crohn’s disease (CD), ulcerative colitis (UC), multiple sclerosis (MS), uveitis, bullous pemphigoid (BP), Hashimoto’s thyroiditis (HT), rheumatoid arthritis (RA), vitiligo, and ankylosing spondylitis (AS). The causal inferences were drawn by integrating results from four regression models: Inverse Variance Weighting (IVW), MR-Egger, Weighted Median, and Maximum Likelihood. Furthermore, we performed sensitivity analyses to confirm the reliability of our findings.

**Results:**

The results showed that CD [IVW odds ratio (OR_IVW_), 1.11; 95% confidence interval (CI), 1.06-1.17; *P* = 8.40E-06], vitiligo (OR_IVW_, 1.16; 95% CI, 1.05-1.28; *P* = 2.45E-03) were risk factors for PsO, while BP may reduce the incidence of PsO (OR_IVW_, 0.91; 95% CI, 0.87-0.96; *P* = 1.26E-04). CD (OR_IVW_, 1.07; 95% CI, 1.02-1.12; *P* = 0.01), HT (OR_IVW_, 1.23; 95% CI, 1.08-1.40; *P* = 1.43E-03), RA (OR_IVW_, 1.11; 95% CI, 1.02-1.21, *P* = 2.05E-02), AS (OR_IVW_, 2.18; 95% CI, 1.46-3.27; *P* = 1.55E-04), SLE (OR_IVW_, 1.04; 95% CI, 1.01-1.08; *P* = 1.07E-02) and vitiligo (OR_IVW_, 1.27; 95% CI, 1.14-1.42; *P* = 2.67E-05) were risk factors for PsA. Sensitivity analyses had validated the reliability of the results.

**Conclusions:**

Our study provides evidence for potential causal relationships between certain AIDs and both PsO and PsA. Specifically, CD and vitiligo may increase the risk of developing PsO, while CD, HT, SLE, RA, AS, and vitiligo may elevate the risk for PsA. Additionally, it is crucial to closely monitor the condition of PsO patients with specific AIDs, as they have a higher likelihood of developing PsA than those without AIDs. Moving forward, greater attention should be paid to PsA and further exploration of other PsO subtypes is warranted.

## 1 Introduction

Psoriasis (PsO), a chronically relapsing dermatological condition with immune involvement, is predominantly featured by erythematous plaques with a scaly appearance (1), affecting approximately 0.11%-1.88% of the global population (2). Since 2014, the World Health Organization has defined PsO by five characteristics: chronic, non-communicable, painful, disfiguring, and disabling (3), exerting a significant psychosocial and economic burden on patients. Approximately 30% of patients with PsO, who only present with skin lesions, will progress to psoriatic arthritis (PsA) (4). PsA is a seronegative inflammatory arthritis, clinically characterized by synovitis with peripheral joint osteolysis, axial involvement, sacroiliitis, nail disorders, and tendinitis (5). In 85% of PsA patients, skin manifestations precede joint symptoms, with joint damage typically appearing about 10 years after the disease onset (6). Furthermore, PsA also shares immunological features with PsO (1), with the IL-23/Th17 pathway playing a central role in the immune regulation and inflammation. The activation of Th1 and Th17 cells and the resultant cytokines they produce, such as IL-17, TNF, and IL-22, drive an inflammatory response that stimulates abnormal proliferation and keratinization of keratinocytes, and exacerbate inflammation in the joints and surrounding soft tissues, leading to cutaneous and skeletal damage (1, 6).

Numerous observational studies have found that PsO and PsA could be comorbid with autoimmune diseases (AIDs) (7, 8). Specifically, research indicated that the risk of comorbidities with AIDs in PsO patients was five times greater than that of the general population (9). Additionally, a South Korean study found a significantly higher risk of rheumatoid arthritis (RA), ankylosing spondylitis (AS), Crohn’s disease (CD), and systemic lupus erythematosus (SLE) in 321,354 PsO patients compared to 321,354 healthy individuals (10). Sardu C et al. selected 25,885 individuals from Sardinia, Italy, to assess the prevalence of 12 AIDs, finding that PsO/PsA ranked second in prevalence within the population (11). Additionally, a retrospective study demonstrated a direct correlation between comorbidity incidence and disease severity in PsO patients, with PsA patients experiencing a significantly higher incidence of comorbidities than those with PsO (12). Although observational studies are widely used for the preliminary etiological investigations, their susceptibility to confounding factors and challenges in determining the temporal sequence of causality make these relationships unclear (13).

Therefore, we performed a Mendelian randomization (MR) analysis to delve deeper into the causal relationships between PsO, PsA, and AIDs such as SLE, multiple sclerosis (MS), CD, ulcerative colitis (UC), uveitis, bullous pemphigoid (BP), Hashimoto’s thyroiditis (HT), RA, AS, and vitiligo. Its principle is founded on Mendel’s Second Law, which states that during meiosis for gamete production, the parental alleles are allocated to the progeny randomly, uninfluenced by environmental, socioeconomic, or other confounding factors. MR analysis utilizes genetic variations as instrumental variables (IVs) to infer causality between exposures and outcomes, thus mitigating the interference from reverse causation and weak confounders (14). It has been extensively applied in exploring complex etiologies of diseases.

## 2 Methods

### 2.1 Study design

**Figure 1:**
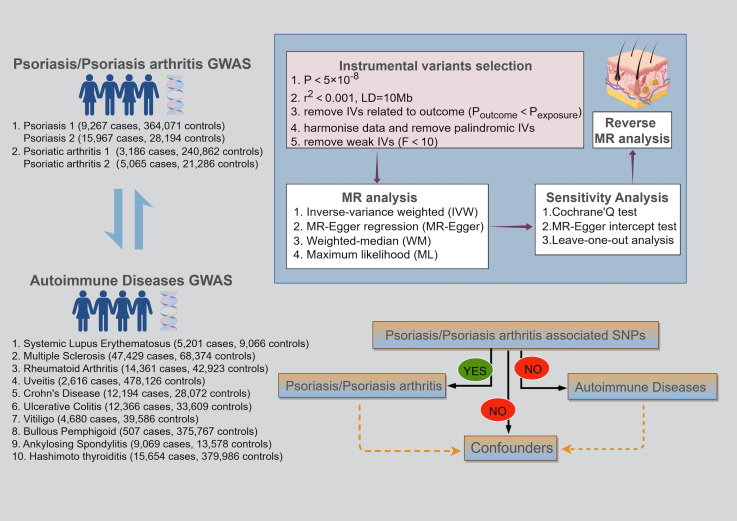
Schematic of the three key assumptions of Mendelian randomization studies and the research flowchart.

We conducted a bidirectional two-sample MR analysis based on the Genome-Wide Association Studies (GWAS) database to explore the causal links between PsO, PsA, and AIDs. This MR study followed three fundamental assumptions: (1) the correlation assumption: the IVs should be strongly correlated with the exposure; (2) the exclusivity assumption: the IVs have no direct relation to the outcome; (3) the independence assumption: the IVs shouldn’t be related to any confounder that affects the exposure-outcome relationship (15, 16) (**Figure 1**).

### 2.2 Data sources

The GWAS databases for PsO (9,267 cases and 364,071 controls), PsA (3,186 cases and 240,862 controls), and BP (507 cases, 375,767 controls) all originated from the FinnGen Consortium, which is a large-scale biomedical research project based in Finland that aims to uncover new biomarkers and therapeutic targets by analyzing the genetic information and health data of Finnish participants. To address the sample overlap among PsO, PsA, and BP, additional GWAS databases were selected for PsO and PsA. Specifically, the GWAS data for PsO (15,967 cases and 28,194 controls) were derived from a cross-ethnic investigation conducted by Stuart PE et al., which compared PsO susceptibility between South Asians and Europeans (17). The GWAS data for PsA (5,065 cases and 21,286 controls) were sourced from a study by Soomro M et al. (18), which developed a database to examine genetic markers differentiating PsA from PsO without arthritis. GWAS data for SLE (5,201 cases and 9,066 controls), MS (47,429 cases and 68,374 controls), CD (12,194 cases and 28,072 controls), UC (12,366 cases and 33,609 controls), uveitis (2,616 cases and 478,126 controls), AS (9,069 cases and 13,578 controls), RA (14,361 cases and 33,609 controls), and HT (15,654 cases and 379,986 controls) were sourced from the IEU database (https://gwas.mrcieu.ac.uk). Moreover, GWAS data for vitiligo (4,680 cases and 39,586 controls) were sourced from the most extensive meta-analyses available (19). The comprehensive GWAS data information is summarized in **Supplementary Table S1**.

### 2.3 Instrumental variables selection

Selecting appropriate IVs requires adherence to six steps. Firstly, a single nucleotide polymorphism (SNP) must demonstrate a strong correlation with the exposure (*p* < 5×10^-8^). Secondly, we set a linkage disequilibrium (LD) threshold (*r^2^*) of 0.001 and a 10 Mb clumping window to guarantee the independence of each SNP (20). Should the number of selected SNPs be insufficient, the thresholds for *p* and *r^2^* can be adjusted to a minimum of *p* < 5×10^-6^ and *r^2^* < 0.01, respectively. Thirdly, we exclude the SNPs that exhibit a strong correlation with the outcome variable (*p_outcome_* < *p_exposure_*). Fourthly, harmonize the data between SNP_exposure_ and SNP_outcome_ to ensure alignment of allelic directions and compatibility for analysis. Fifthly, confounders are eliminated through the Phenoscanner website to mitigate potential pleiotropic effects (21). Finally, the association strength between IVs and the exposure is measured using F-statistics, computed as *F*=*R^2^*/(1-*R^2^*)×(*N*-*K*-1)/*K*, with *R^2^ = *2×*MAF*×(1-*MAF*)×*β^2^*. In the absence of *MAF* values, *R^2^* is determined by *R^2^* =*β^2^*/(*β^2^* +*SE^2^* ×*N*) *(* 22–24), where *R^2^* indicates the variance in exposure explained by the IVs, *N* represents the total sample size of the exposure GWAS, *MAF* denotes the frequency of minor allele, *K* refers to the number of IVs, and *β* signifies the SNP’s effect size on exposure. If the F-statistic exceeds 10, it implies that MR analyses are unlikely to be biased by weak IVs. Conversely, SNPs with F-statistics below this threshold should be excluded.

### 2.4 Mendelian randomization analysis

MR analysis primarily employs Inverse Variance Weighted (IVW) to determine the existence of causality, while MR Egger (ME), Weighted Median (WM), and Maximum Likelihood (ML) serve as supplementary analytical approaches. IVW is characterized by its disregard for the intercept term, fitting the data using the inverse of the outcome variance as weights (25). Unlike IVW, ME incorporates the intercept term within its regression model, also applying the inverse of outcome variance as weights for fitting (26). WM is the median of the distribution function derived from sorting all SNP effect values by their weights. It delivers a reliable causal effect estimate, despite having up to 50% invalid IVs (27). ML, grounded in principles of probability theory, estimates unknown parameters by identifying model parameters that maximize the likelihood of the observed data (28). If *P* < 0.05 for IVW, a causal link between exposure and outcome is inferred when all five conditions are met simultaneously: (1) at least one other statistical method yields a p-value < 0.05; (2) the odds ratios (OR) from IVW, WM, and ML consistently indicate the same direction of effect; (3) there is no significant evidence of horizontal pleiotropy (*P* > 0.05); (4) all error lines of the leave-one-out analysis plot are all on the same side of zero; (5) these conditions still hold after adjusting for heterogeneity. This study conducted MR analysis using R software (version 4.2.3), utilizing the R packages TwoSampleMR and RadialMR.

### 2.5 Sensitivity analyses

We employed Cochran’s Q test, leave-one-out analysis, and the MR-Egger intercept for sensitivity analysis. The objectives of the sensitivity analyses are threefold: first, to evaluate the dependability of the MR analysis outcomes; second, to explore potential biases, such as genetic pleiotropy and data heterogeneity; and third, to determine whether a specific SNP significantly affects the outcome. We employed Cochran’s Q test to assess the extent of heterogeneity. When significant heterogeneity occurs (*P* < 0.05), we employ MR radial analysis to remove outliers and correct the estimates to verify the reliability of the findings. Leave-one-out analysis determines the combined effect of the remaining SNPs by sequentially excluding each SNP, with all error lines located consistently on one side of zero, suggesting dependable outcomes. Furthermore, MR studies should primarily focus on the horizontal pleiotropy to avoid genetic variants influencing the outcome through exposure. If the intercept of the MR-Egger regression is significantly different from zero (*P* < 0.05), it indicates the presence of horizontal pleiotropy, and we use MR radial analysis to correct the estimates by excluding outliers.

## 3 Results

### 3.1 Results of selection of instrumental variables

Following the described selection process, we identified IVs (**Supplementary Tables S7-S10**). Specifically, in studying the effects of BP and uveitis on PsO, we established strong correlation and LD for BP (*P* < 5×10^-6^, *r^2^* < 0.01) and uveitis (*P* < 5×10^-6^, *r^2^* < 0.001) to extract a sufficient number of SNPs. Furthermore, in investigating the effect of CD on PsA, we set *P* < 5×10^-9^, *r^2^* < 0.001 to eliminate horizontal pleiotropy. Ultimately, we calculated that the F value corresponding to each SNP (**Supplementary Tables S7-S10**) or to all SNPs (**Supplementary Table S11**) was greater than 10, indicating that the results were not biased by weak IVs.

### 3.2 Impact of PsO and PsA on AIDs

When considering PsO and PsA as exposures, there were no causal relationships between them and AIDs (**Supplementary Figures S1, S2 Supplementary Tables S2, S5**).

### 3.3 Impact of AIDs on PsO and PsA

**Figure 2:**
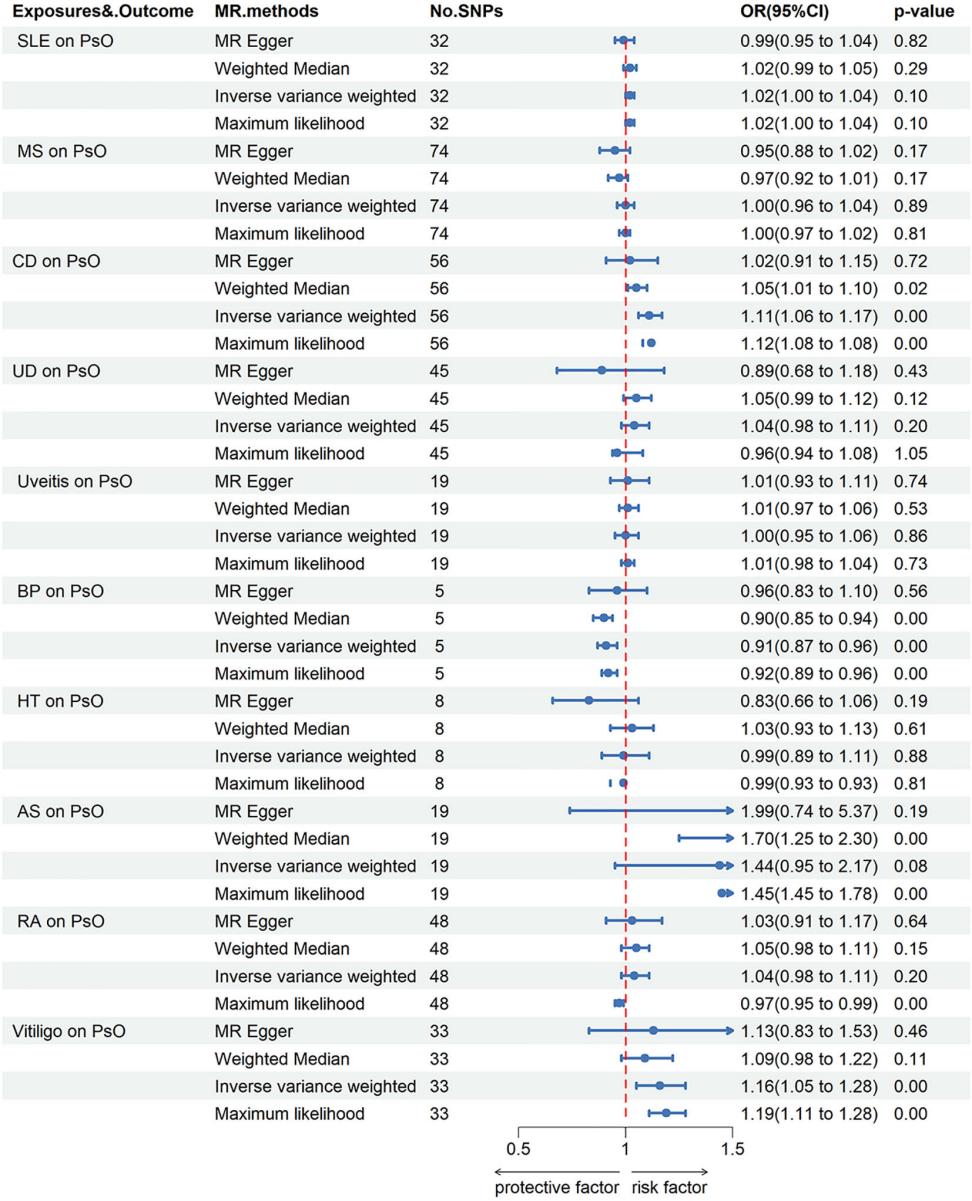
Forest plots utilized four methods to visualize the causal effects of AIDs on PsO risk. PsO, psoriasis; SLE, Systemic lupus erythematosus; MS, multiple sclerosis; RA, Rheumatoid arthritis; UD, Crohn’s disease; UC, Ulcerative colitis; Vitiligo; BP, Bullous pemphigoid; AS, Ankylosing spondylitis; HT, Hashimoto thyroiditis;AIDs, Autoimmune diseases.

**Figure 3:**
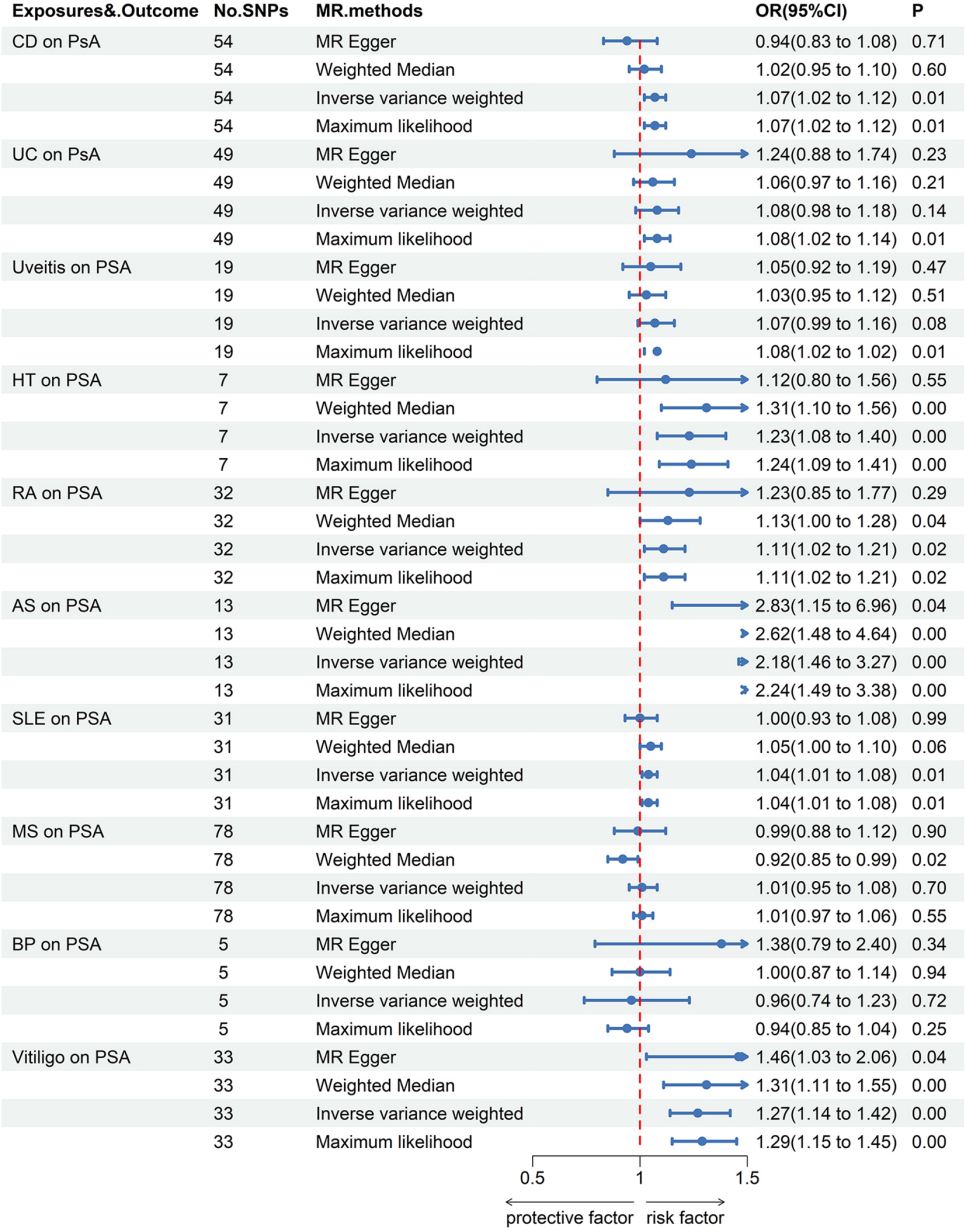
Forest plots utilized four methods to visualize the causal effects of AIDs on PsA risk. PsA, psoriasis arthritis; SLE, Systemic lupus erythematosus; MS, multiple sclerosis; RA, Rheumatoid arthritis; UD, Crohn’s disease; UC, Ulcerative colitis; Vitiligo; BP, Bullous pemphigoid; AS, Ankylosing spondylitis; HT, Hashimoto thyroiditis;AIDs, Autoimmune diseases.

When PsO and PsA were considered as outcomes, genetically predicted CD [IVW odds ratio (OR_IVW_), 1.11; 95% confidence interval (CI), 1.06-1.17; *P* = 8.40E-06], vitiligo (OR_IVW_, 1.16; 95% CI, 1.05-1.28; *P* = 2.45E-03) were risk factors for PsO, while BP may reduce the incidence of PsO (OR_IVW_, 0.91; 95% CI, 0.87-0.96; *P* = 1.26E-04). Furthermore, no causal link was found between the remaining AIDs and PsO. CD (OR_IVW_, 1.07; 95% CI, 1.02-1.12; *P* = 0.01), HT (OR_IVW_, 1.23; 95% CI, 1.08-1.40; *P* = 1.43E-03), RA (OR_IVW_, 1.11; 95% CI, 1.02-1.21; *P* = 2.05E-02), AS (OR_IVW_, 2.18; 95% CI, 1.46-3.27; *P* = 1.55E-04), SLE (OR_IVW_, 1.04; 95% CI, 1.01-1.08; *P* = 1.07E-02) and vitiligo (OR_IVW_, 1.27; 95% CI, 1.14-1.42; *P* = 2.67E-05) were risk factors for PsA. There were no causal associations found between the remaining AIDs and PsO/PsA (**Figures 1**–**3**; **Supplementary Tables S3, S4**).

### 3.4 Results of sensitivity analysis

In addition to the previously mentioned pleiotropy between CD and PsA, we also found pleiotropy between SLE and PsO (*P* = 0.038). Therefore, we utilized the MR-Radial to remove five outlier SNPs—rs389884, rs4274624, rs4388254, rs58688157, and rs58721818—which effectively mitigated pleiotropy. In studies with causal relationships, significant heterogeneity was observed when CD and vitiligo affected PsO. Therefore, we used MR-Radial to reduce heterogeneity and found that the positive results remained stable (**Supplementary Figure S19**, **Supplementary Table S6**). Furthermore, in a leave-one-out analysis, excluding each SNP individually did not result in significant variation in the estimated causal effects (**Supplementary Figures S3-S18**). In summary, our study findings are deemed reliable and valid.

## 4 Discussions

To date, this study represents the first large-scale MR analysis to explore the causal associations between both PsO and PsA and ten types of AIDs. Genetic prediction results indicate that BP might lower the risk of developing PsO, while CD and vitiligo may increase it. Additionally, certain AIDs including CD, AS, RA, HT, vitiligo, and SLE are more likely to induce PsA. This study’s findings align with those observed in an earlier cohort study, which revealed that most AIDs were diagnosed prior to PsO (9).

CD is an inflammatory bowel disorder characterized by abdominal pain, diarrhea, and bloody stool (29). A meta-analysis incorporating nine observational studies indicated a significantly elevated chance of developing CD and UC in patients with PsO (30). Nonetheless, a United States cohort study showed a significantly higher CD risk in female PsA patients, with no increased UC risk, matching our genetic predictions (31). Genetic susceptibility is a key determinant of the underlying risk of both PsO and CD, with strong associations confirmed at the IL23R, IL12B, REL, and TYK2 loci for both conditions (32). Moreover, both PsO and CD exhibit dysbiosis of the gut microbiota, which can disrupt the immune balance between effector T cells and regulatory T (Treg) cells. This imbalance could also increase intestinal permeability, allowing gut bacteria and their metabolites to enter the skin through the bloodstream, thereby triggering inflammatory skin responses (33, 34). Additionally, dysbiosis can increase the levels of hydrogen sulfide in the gut while inhibiting the production of protective metabolites such as butyrate and propionate, thus potentially triggering the onset of CD (32). Furthermore, the TNF pathway (35), IL-23/IL-17 pathway (36), JAK-STAT pathway (37), and ROR-γT/Th17 axis (38) represent common pathogenic pathways between PsO and inflammatory bowel diseases.

BP is primarily characterized by tense, non-rupturing large blisters (39). A cohort study conducted in Taiwan revealed that the risk of developing PsO among patients with BP was significantly higher than that in the healthy population (40). Ohata et al. conducted a study on patients diagnosed with both psoriasis and autoimmune blistering diseases (AIBD). The results indicated that BP was the most prevalent form of AIBD, affecting 63.4% of the patients (41). Some scholars believe that certain treatment modalities for PsO, such as corticosteroids, anti-IL-17A monoclonal antibodies, tumor necrosis factor-alpha (TNF-α) antagonists, and ultraviolet light therapy, might trigger the onset of BP (42–44). Furthermore, the release of a large amount of neutrophil chemotactic factors by PsO could initiate a cascade of reactions leading to BP (45); additionally, the disruption or even absence of laminin in the psoriatic skin lesions affects the differentiation and proliferation of keratinocytes, potentially inducing BP through antibodies (such as those targeting the basement membrane zone) (46). Lastly, the involvement of Th17 cells and IL-17 promotes the production of pro-inflammatory cytokines and matrix metalloproteinases, culminating in blister formation (47). However, our study results do not align with the findings from epidemiological research. This discrepancy may be attributed to the small sample size of the GWAS for BP and the insufficient strength of the IVs used (*P* < 5×10^-6^, *r^2^* < 0.01). Therefore, caution should be exercised when interpreting the Mendelian Randomization results between BP and PsO.

Vitiligo is an immune-mediated depigmenting disease (48). Previous literature has shown that vitiligo often precedes the onset of PsO (49, 50), aligning with our findings on the causal sequence. A retrospective study on the prevalence of comorbid conditions in vitiligo patients revealed that out of 2,441 vitiligo patients, 565 (23%) had comorbid AIDs, with thyroid diseases and psoriasis being the most common (51). A meta-analysis encompassing ten observational studies revealed a notably higher incidence of PsO among vitiligo patients compared to the general population (52). In terms of genetic susceptibility, Zhu et al. identified that rs9468925 within the HLA-C/HLA-B locus is associated with both PsO and vitiligo (53). In the pathogenesis of vitiligo, CD8+ T cells play a predominant role (54), while the Th17 pathway and IL-17 also exert multiple effects (55): firstly, IL-17 attracts CD8+ T cells into the surrounding tissue, directly causing the destruction of melanocytes. Secondly, IL-17 enhances the inflammatory response of endothelial cells and keratinocytes, promoting the migration and infiltration of inflammatory cells such as neutrophils and T cells. Neutrophils can increase the production of ROS, inducing oxidative stress and further damaging melanocytes. Moreover, IL-17 promotes melanocyte apoptosis by inhibiting the expression of MITF and downregulating BCL2 (56). NB-UVB treatment alleviates oxidative stress responses and improves the condition by reducing IL-17 levels (57).

HT, an autoimmune disorder, is characterized by hypothyroidism resulting from thyroid gland dysfunction (58). Numerous studies have identified a link between PsA and autoimmune thyroiditis (59, 60). Bianchi et al. observed that patients with PsA have significantly more thyroid involvement compared to the general population, evidenced by an increased average thyroid volume and higher prevalence rates of anti-microsomal and anti-thyroglobulin antibodies (61). Both HT and PsA are autoimmune diseases mediated by Th1 cell immunity, with Th1 cells, interferon-gamma (IFN-γ), and the chemokine CXCL10 playing pivotal roles in their pathogenesis (6, 62). In HT, Th1 cells produce IFN-γ and TNF-α, stimulating thyroid cells to emit CXCL10. This chemokine binds to receptor CXCR3, attracting Th1 cells to the target tissue, thereby triggering inflammatory responses and thyroid damage (58). Conversely, many PsA patients show increased CXCL10 levels in their serum and synovial fluid (63, 64), capable of attracting plasmacytoid dendritic cells from the blood into the synovial tissue, thereby initiating an inflammatory response (65).

SLE is an autoimmune connective tissue disease that affects multiple organs (66). A case-control study from Israel found that PsA patients exhibited a 2.3-fold higher prevalence of SLE compared to the control group (67). It has been found that PsO and SLE have shared genetic predisposition sites, including PTPN22, TRAF3IP2, and STAT4. Regarding immune mechanisms, the high expression of IL-17/IL-23 axis is crucial for the comorbidity of SLE and PsO. Patients with SLE, due to defects in cell apoptosis, release large amounts of dsDNA and ribonucleoproteins. These substances form nucleic acid immune complexes upon binding with autoantibodies, subsequently activating plasmacytoid dendritic cells (pDCs). Activated pDCs secrete IL-23, stimulating the differentiation and proliferation of Th17 cells and the production of IL-17. IL-17 promotes the proliferation and differentiation of B cells into plasma cells, producing autoantibodies, stimulating the generation of a large number of inflammatory cells, and thereby damaging target organs (68–70). Additionally, a case report detailed the treatment of SLE and PsA with the IL-17 inhibitor secukinumab (71), and a double-blind Phase II trial validated the effectiveness of an IL-23 antagonist in treating SLE. These findings underscore the critical role of the IL-17/IL-23 axis in the development of both SLE and PsA.

RA and AS are autoimmune diseases primarily characterized by bone damage and pain (72). AS predominantly targets the sacroiliac joints, spine, and peripheral joints, whereas RA mainly leads to synovitis, bone erosion, and cartilage damage (72, 73). Prior research has indicated a higher prevalence of AS and RA in patients with PsA compared to those with PsO alone (74, 75). A cohort study from the UK discovered that AS patients experienced a higher risk of comorbidities with PsO, with 4.4% of individuals initially diagnosed with AS also presenting with concurrent PsO (76). There has been ongoing debate regarding whether AS with PsO manifestations inherently constitutes a form of axial PsA (77). This study suggests a causal link between AS and PsA, inferring that AS with PsO manifestations might indeed be an expression of co-occurring PsA and AS. And HLA-B*27 is a shared genetic susceptibility factor for both conditions (78, 79). Moreover, the IL-23/Th17 is also involved in the pathogenesis of AS, with heightened expression of inflammatory cytokines like IL-17 and IL-22 influencing pertinent signaling pathways (80). This leads to enhanced activity of osteoclasts and inhibited function of osteoblasts, resulting in bone damage. Consequently, the IL-17A inhibitor—secukinumab—has shown effectiveness in treating PsA and AS in a phase III randomized clinical trial, further evidencing their overlapping mechanisms of action (81). Previous studies have demonstrated comorbidity of PsA with RA, primarily affecting peripheral joints and manifesting as symmetrical polyarthritis with positive rheumatoid serology. Th17 cells have emerged as research targets in clinical trials for RA (82), with the IL-17A produced by Th17 cells acting synergistically with TNF-α to promote the activation of fibroblasts and chondrocytes (73). Moreover, AS and RA can cause bone and joint damage, which is independently linked to a higher risk of developing PsA (83). This may relate to physical trauma activating innate immunity, leading to the influx of pro-inflammatory molecules into the synovium (84), or activating nerve endings to release neuropeptides, triggering an inflammatory response (85).

In addition, multiple epidemiological studies suggest that PsO and PsA are comorbid with uveitis and MS. 10% of PsO patients and 30% of PsA patients may experience ocular diseases, with uveitis being the most common (86–88). A cohort study from Korea indicated that individuals with severe PsO or PsA exhibit an elevated incidence and recurrence rate of uveitis compared to controls (89). Furthermore, a comprehensive cohort study in Denmark showed that the higher the severity of PsO, the greater the risk of developing MS (90). However, this study did not find a causal link, suggesting that potential confounders or a shared genetic architecture might be the reason behind the epidemiological associations.

In summary, our study has three important implications: (1) It provides evidence of potential causal relationships between certain AIDs and both PsO and PsA, suggesting that increased surveillance of these conditions should be considered in clinical practice; (2) Given that AIDs are more likely to precipitate the occurrence of PsA and most PsA patients initially present only with skin lesions, it can be inferred that patients with PsO and comorbid AIDs are more likely to develop PsA. It indicates that AIDs could be potential risk factors for progressing from PsO to PsA, underscoring the importance of early monitoring for PsA; (3) This study also underscores the critical importance of establishing GWAS database for PsO subtypes, which is vital for future etiological research into PsO. However, our study also presents several limitations. Firstly, the lack of pertinent GWAS data prevents us from exploring the causal links between different subtypes. Secondly, the GWAS database we used primarily targeted European populations, potentially limiting its applicability to other ethnicities. Thirdly, the small GWAS dataset for BP in our study necessitates larger future datasets for validating our findings. Fourthly, although using multiple methods to control confounders, potential horizontal pleiotropy may still exist. Lastly, this study may have overlooked other AIDs are causally linked to PsO and PsA.

## 5 Conclusions

Certain AIDs are causally associated with PsO and PsA. Furthermore, PsO patients who have certain AIDs are more prone to developing PsA than those without AIDs. Additionally, focusing more on PsO subtypes could enhance our understanding of the condition.

## Data availability statement

The detailed data and GWAS data sources referenced in this study are available in the article/**Supplementary Materials**. Should you require further data, please contact the author directly.

## Ethics statement

All data utilized herein were derived from the public genome-wide association studies, thus obviating the need for further ethical consent.

## Author contributions

KD: Writing – review & editing, Writing – original draft, Software, Methodology, Formal analysis, Data curation. JRW: Writing – original draft, Data curation, Conceptualization. SC: Writing – original draft, Methodology, Data curation. TC: Writing – original draft, Methodology. JJW: Writing – review & editing, Data curation. SW: Writing – review & editing, Data curation. XC: Writing – review & editing, Supervision, Funding acquisition, Conceptualization.
