# Supplementary material for: Causal associations between both psoriasis and psoriatic arthritis and multiple autoimmune diseases: a bidirectional two-sample Mendelian randomization study
Source: Front Immunol. 2024 Jul 25;15:1422626. doi: 10.3389/fimmu.2024.1422626 (PMC11306030; doi:10.3389/fimmu.2024.1422626)
Supplement: Supplementary file 1 [file DataSheet_1.pdf]

Causal Evidence between Mutiple Autoimmune Diseases and Both Psoriasis and Psoriatic Arthritis:

A Bidirectional Two-Sample Mendelian Randomization Study

Kexin Duan[1], Jingrui Wang[2], Shaomin Chen[1], Tong Chen[1], Jiajue Wang[1], Shujing Wang[2], Xinsheng Chen[3]*

[1] The Second Clinical Medical College, Guangzhou University of Chinese Medicine, Guangzhou, Guangdong, China

[2] The First Clinical Medical College, Guangzhou University of Chinese Medicine, Guangzhou, Guangdong, China

[3] Department of Dermatology, Guangdong Provincial Hospital of Traditional Chinese Medicine, Guangzhou, Guangdong, China

* Xinsheng Chen is the corresponding author. Email: xinshengchen@sihu.com

Supplements

Figure S1. Forest plots utilized four methods to visualize the causal effects of PsO on AIDs risk 3

Figure S2. Forest plots utilized four methods to visualize the causal effects of PsA on AIDs risk 4

Figure S3. Visualization of the MR analysis of the effect of PsO on vitiligo, BP, and MS 6

Figure S4. Visualization of the MR analysis of the effect of PsO on CD, UC, and RA 8

Figure S5. Visualization of the MR analysis of the effect of PsO on Uveitis, AS, and HT 10

Figure S6. Visualization of the MR analysis of the effect of PsO on SLE 11

Figure S7. Visualization of the MR analysis of the effect of PsA on vitiligo, MS, and CD 13

Figure S8. Visualization of the MR analysis of the effect of PsA on UC, RA, and Uveitis 16

Figure S9. Visualization of the MR analysis of the effect of PsA on AS, BP, and HT 18

Figure S10. Visualization of the MR analysis of the effect of PsA on SLE 19

Figure S11. Visualization of the MR analysis of the effect of MS, CD, and UC on PsO 21

Figure S12. Visualization of the MR analysis of the effect of RA, BP, and Uveitis on PsO 23

Figure S13. Visualization of the MR analysis of the effect of AS, HT, and SLE on PsO 25

Figure S14. Visualization of the MR analysis of the effect of Vitiligo on PsO 26

Figure S15. Visualization of the MR analysis of the effect of SLE, HT, and AS on PsO 28

Figure S16. Visualization of the MR analysis of the effect of Uveitis, BP, and RA on PsA 30

Figure S17. Visualization of the MR analysis of the effect of UC, CD, and MS on PsA 32

Figure S18. Visualization of the MR analysis of the effect of Vitiligo on PsA 33

Figure S19. Visualization of the MR analysis of the effect of Vitiligo and CD on PsO after reducing heterogeneiy 34

Table S1. Detail information of traits involved in this study. 35

Table S2. The causal effects of PsO on AIDs with heterogeneity and pleiotropy test results. 36

Table S3. The causal effects of AIDs on PsO with heterogeneity and pleiotropy test results. 38

Table S4. The causal effects of AIDs on PsA with heterogeneity and pleiotropy test results. 41

Table S5. The causal effects of PsA on AIDs with heterogeneity and pleiotropy test results. 44

Table S6. The causal effects of CD and vitiligo on on PsO with heterogeneity and pleiotropy test resultss after reduce heterogeneiy 46

Table S7. Detailed information of instrumental variables utilized in the Mendelian randomization analysis of PsO on AIDs. 47

Table S8. Detailed information of instrumental variables utilized in the Mendelian randomization analysis of PsA on AIDs. 57

Table S9. Detailed information of instrumental variables utilized in the Mendelian randomization analysis of AIDs on PsA. 62

Table S10. Detailed information of instrumental variables utilized in the Mendelian randomization analysis of AIDs on PsO. 78

Table S11. F-statistics for all MR analyses. 96

Exposures&.Outcome	MR.methods	No.SNPs	OR(95%CI)	p-value
PsO on SLE	MR Egger	21	1.03(0.56 to 1.90)	0.94
	Weighted Median	21	0.94(0.78 to 1.14)	0.52
	Inverse variance weighted	21	0.91(0.74 to 1.11)	0.35
	Maximum likelihood	21	0.91(0.81 to 1.01)	0.08
PsO on MS	MR Egger	19	1.14(0.56 to 2.31)	0.72
	Weighted Median	19	0.99(0.87 to 1.13)	0.93
	Inverse variance weighted	19	1.06(0.87 to 1.29)	0.56
	Maximum likelihood	19	1.07(0.99 to 1.15)	0.11
PsO on CD	MR Egger	17	1.25(0.80 to 1.96)	0.34
	Weighted Median	17	1.03(0.90 to 1.17)	0.69
	Inverse variance weighted	17	1.06(0.90 to 1.25)	0.47
	Maximum likelihood	17	1.07(0.99 to 1.15)	0.08
PsO on UD	MR Egger	20	1.10(0.66 to 1.83)	0.73
	Weighted Median	20	1.15(1.00 to 1.32)	0.04
	Inverse variance weighted	20	1.14(0.96 to 1.35)	0.14
	Maximum likelihood	20	1.15(1.07 to 1.23)	0.00
PsO on Uveitis	MR Egger	28	0.88(0.66 to 1.17)	0.38
	Weighted Median	28	0.81(0.70 to 0.93)	0.00
	Inverse variance weighted	28	1.01(0.86 to 1.18)	0.89
	Maximum likelihood	28	1.01(0.93 to 1.10)	0.78
PsO on BP	MR Egger	44	0.95(0.67 to 1.34)	0.76
	Weighted Median	44	1.06(0.86 to 1.31)	0.56
	Inverse variance weighted	44	1.15(1.00 to 1.33)	0.06
	Maximum likelihood	44	1.15(1.00 to 1.32)	0.05
PsO on HT	MR Egger	28	0.94(0.79 to 1.12)	0.50
	Weighted Median	28	0.93(0.86 to 1.00)	0.05
	Inverse variance weighted	28	0.97(0.88 to 1.06)	0.46
	Maximum likelihood	28	0.97(0.92 to 1.01)	0.16
PsO on AS	MR Egger	11	1.06(0.86 to 1.31)	0.57
	Weighted Median	11	1.04(1.00 to 1.09)	0.04
	Inverse variance weighted	11	1.04(1.00 to 1.08)	0.08
	Maximum likelihood	11	1.04(1.01 to 1.07)	0.00
PsO on RA	MR Egger	22	1.11(0.76 to 1.61)	0.61
	Weighted Median	22	0.95(0.87 to 1.04)	0.29
	Inverse variance weighted	22	0.97(0.86 to 1.10)	0.66
	Maximum likelihood	22	0.97(0.97 to 1.03)	0.35
PsO on Vitiligo	MR Egger	13	0.88(0.56 to 1.37)	0.57
	Weighted Median	13	0.98(0.82 to 1.17)	0.85
	Inverse variance weighted	13	1.02(0.89 to 1.18)	0.75
	Maximum likelihood	13	1.02(0.89 to 1.18)	0.74

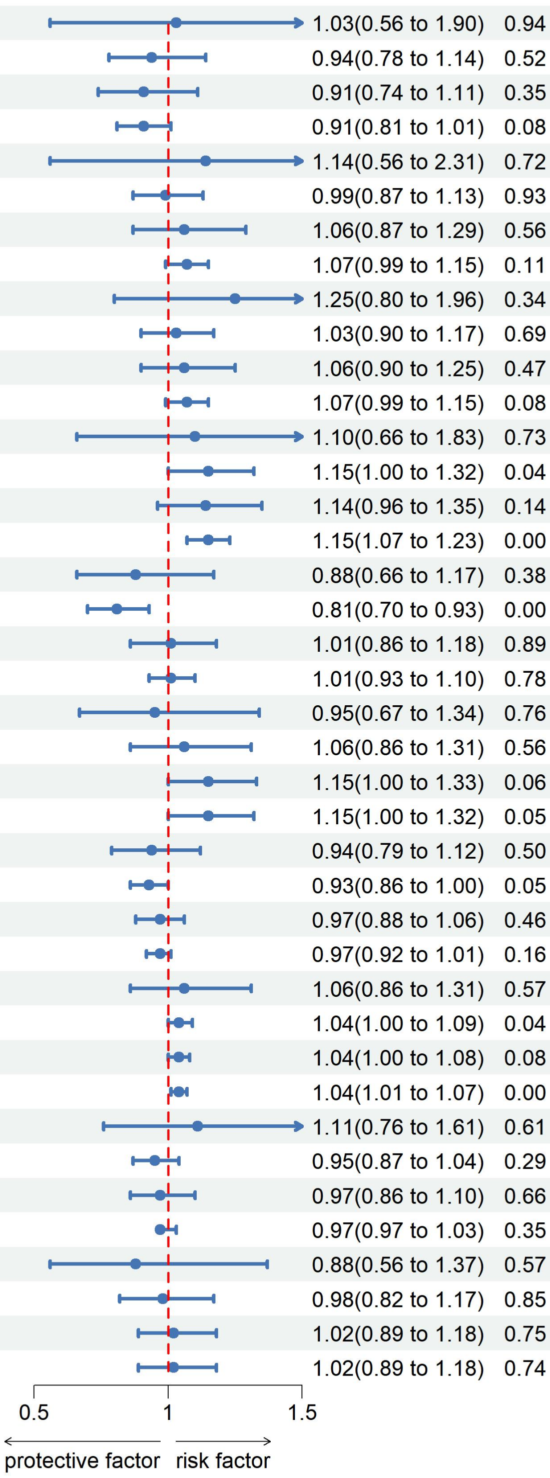

Figure S1. Forest plots utilized four methods to visualize the causal effects of PsO on AIDs risk

Abbreviations: PsO, psoriasis; SLE, Systemic lupus erythematosus; MS, multiple sclerosis; RA, Rheumatoid arthritis; UD, Crohn's disease; UC, Ulcerative colitis; Vitiligo; BP, Bullous pemphigoid; AS, Ankylosing spondylitis; HT, Hashimoto thyroiditis;AIDs, Autoimmune diseases.

Exposures&.Outcome	No.SNPs	MR.methods	OR(95%CI)	P
PsA on CD	9	MR Egger	0.89(0.37 to 2.16)	0.81
	9	Weighted Median	1.05(0.94 to 1.16)	0.40
	9	Inverse variance weighted	1.02(0.87 to 1.19)	0.80
	9	Maximum likelihood	1.02(1.02 to 1.09)	0.49
PsA on UD	9	MR Egger	1.07(0.92 to 1.25)	0.42
	9	Weighted Median	0.99(0.94 to 1.05)	0.86
	9	Inverse variance weighted	0.98(0.90 to 1.05)	0.53
	9	Maximum likelihood	0.98(0.93 to 1.02)	0.31
PsA on Uveitis	11	MR Egger	0.75(0.39 to 1.42)	0.40
	11	Weighted Median	0.90(0.77 to 1.05)	0.18
	11	Inverse variance weighted	0.94(0.75 to 1.19)	0.62
	11	Maximum likelihood	0.94(0.86 to 1.03)	0.19
PsA on HT	13	MR Egger	0.88(0.79 to 0.99)	0.06
	13	Weighted Median	0.95(0.89 to 1.01)	0.08
	13	Inverse variance weighted	0.96(0.91 to 1.02)	0.15
	13	Maximum likelihood	0.96(0.92 to 1.00)	0.03
PsA on RA	9	MR Egger	1.18(0.57 to 2.46)	0.67
	9	Weighted Median	0.95(0.85 to 1.06)	0.33
	9	Inverse variance weighted	0.96(0.84 to 1.09)	0.49
	9	Maximum likelihood	0.95(0.91 to 1.00)	0.03
PsA on AS	6	MR Egger	0.96(0.57 to 1.64)	0.90
	6	Weighted Median	1.00(0.97 to 1.03)	0.91
	6	Inverse variance weighted	1.00(0.95 to 1.06)	0.94
	6	Maximum likelihood	1.00(0.98 to 1.02)	0.82
PsA on SLE	7	MR Egger	0.68(0.18 to 2.64)	0.61
	7	Weighted Median	0.92(0.79 to 1.06)	0.25
	7	Inverse variance weighted	1.01(0.83 to 1.23)	0.89
	7	Maximum likelihood	1.02(0.93 to 1.11)	0.71
PsA on MS	9	MR Egger	0.68(0.46 to 1.00)	0.09
	9	Weighted Median	0.77(0.69 to 0.87)	0.00
	9	Inverse variance weighted	0.89(0.72 to 1.10)	0.30
	9	Maximum likelihood	0.89(0.82 to 0.96)	0.00
PsA on BP	15	MR Egger	1.06(0.78 to 1.42)	0.72
	15	Weighted Median	1.04(0.86 to 1.26)	0.66
	15	Inverse variance weighted	1.02(0.89 to 1.18)	0.74
	15	Maximum likelihood	1.02(0.90 to 1.17)	0.73
PsA on Vitiligo	5	MR Egger	1.25(0.64 to 2.44)	0.55
	5	Weighted Median	1.01(0.85 to 1.20)	0.89
	5	Inverse variance weighted	1.04(0.90 to 1.20)	0.60
	5	Maximum likelihood	1.04(0.90 to 1.20)	0.60

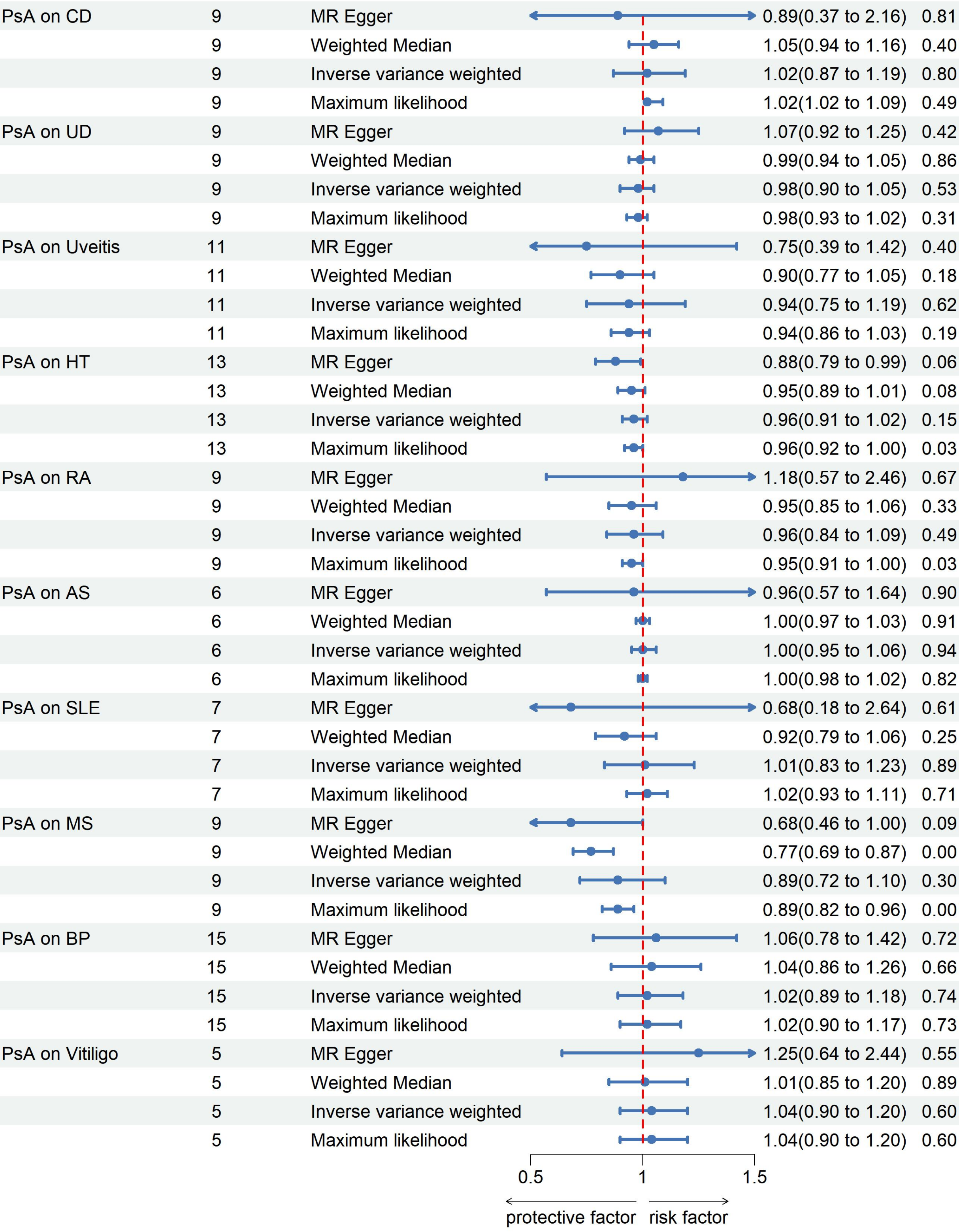

Figure S2. Forest plots utilized four methods to visualize the causal effects of PsA on AIDs risk.

Abbreviations: PsA, psoriatic arthritis; SLE, Systemic lupus erythematosus; MS, multiple sclerosis; RA, Rheumatoid arthritis; CD, Crohn's disease; UC, Ulcerative colitis; Vitiligo; BP, Bullous pemphigoid; AS, Ankylosing spondylitis; HT, Hashimoto thyroiditis; AIDs, Autoimmune diseases.

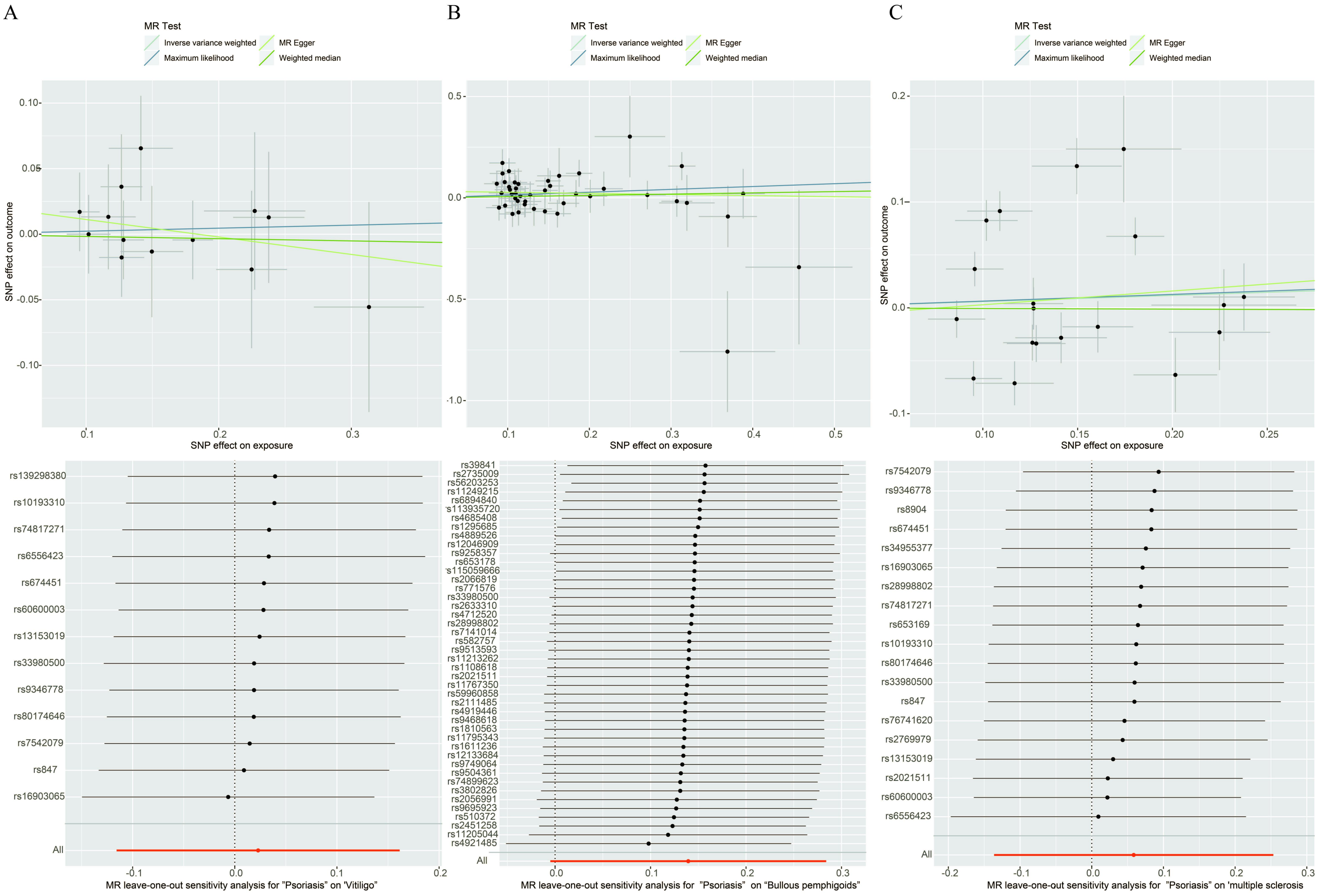
A
MR Test
Inverse variance weighted
MR Egger
Maximum likelihood
Weighted median
SNP effect on outcome
SNP effect on exposure
rs139298380
rs10193310
rs74817271
rs6556423
rs674451
rs60600003
rs13153019
rs33980500
rs9346778
rs80174646
rs7542079
rs847
rs16903065
All
MR leave-one-out sensitivity analysis for "Psoriasis" on 'Vitiligo"
B
MR Test
Inverse variance weighted
MR Egger
Maximum likelihood
Weighted median
SNP effect on outcome
SNP effect on exposure
rs39841
rs2735009
rs56203253
rs11249215
rs6894840
s113935720
rs4685408
rs1295685
rs4889526
rs12046909
rs9258357
rs653178
s115059666
rs2066819
rs771576
rs33980500
rs2633310
rs4712520
rs28998802
rs7141014
rs582757
rs9513593
rs11213262
rs1108618
rs2021511
rs11767350
rs59960858
rs2111485
rs4919446
rs9468618
rs1810563
rs11795343
rs1611236
rs12133684
rs9749064
rs9504361
rs74899623
rs3802826
rs2056991
rs9695923
rs510372
rs2451258
rs11205044
rs4921485
All
MR leave-one-out sensitivity analysis for "Psoriasis" on "Bullous pemphigoids"
C
MR Test
Inverse variance weighted
MR Egger
Maximum likelihood
Weighted median
SNP effect on outcome
SNP effect on exposure
rs7542079
rs9346778
rs8904
rs674451
rs34955377
rs16903065
rs28998802
rs74817271
rs653169
rs10193310
rs80174646
rs33980500
rs847
rs76741620
rs2769979
rs13153019
rs2021511
rs60600003
rs6556423
All
MR leave-one-out sensitivity analysis for "Psoriasis" on 'multiple sclerosis

Figure S3. Visualization of the MR analysis of the effect of PsO on vitiligo, BP, and MS. (A) Scatter plot of the MR analysis and Leave-one-out sensitivity analysis of the effect psoriasis on vitiligo; (B) Scatter plot of the MR analysis and Leave-one-out sensitivity analysis of the effect of psoriasis on BP. (C) Scatter plot of the MR analysis and Leave-one-out sensitivity analysis of the effect of psoriasis on MS.

Abbreviations: MR, Mendelian randomization; BP, Bullous pemphigoid; MS, Multiple sclerosis.

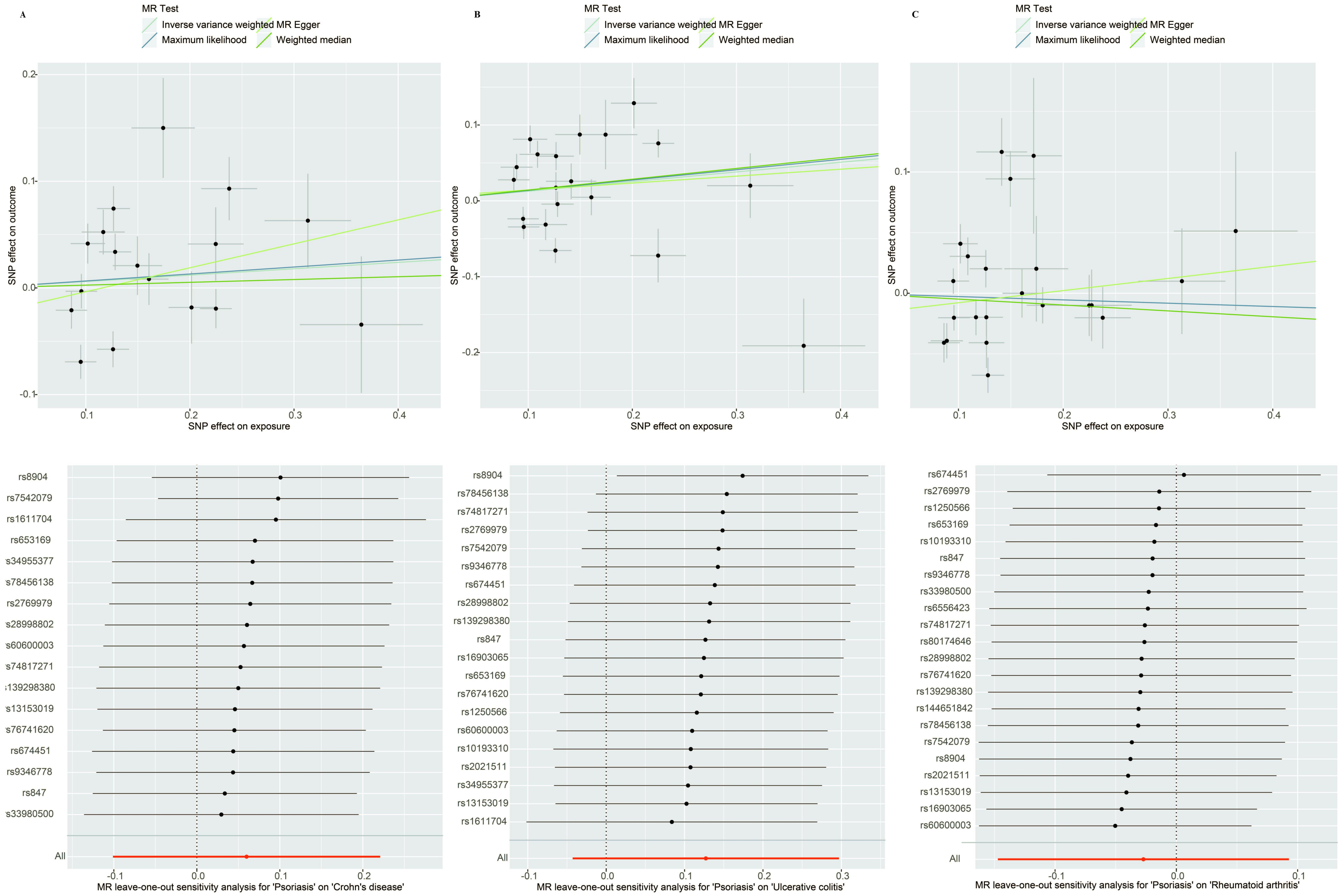

A
B
C
MR Test
Inverse variance weighted
MR Egger
Maximum likelihood
Weighted median
SNP effect on outcome
SNP effect on exposure
rs8904
rs7542079
rs1611704
rs653169
s34955377
s78456138
rs2769979
s28998802
s60600003
s74817271
:139298380
s13153019
s76741620
rs674451
rs9346778
rs847
s33980500
All
MR leave-one-out sensitivity analysis for 'Psoriasis' on 'Crohn's disease'
rs8904
rs78456138
rs74817271
rs2769979
rs7542079
rs9346778
rs674451
rs28998802
rs139298380
rs847
rs16903065
rs653169
rs76741620
rs1250566
rs60600003
rs10193310
rs2021511
rs34955377
rs13153019
rs1611704
All
MR leave-one-out sensitivity analysis for 'Psoriasis' on 'Ulcerative colitis'
rs674451
rs2769979
rs1250566
rs653169
rs10193310
rs847
rs9346778
rs33980500
rs6556423
rs74817271
rs80174646
rs28998802
rs76741620
rs139298380
rs144651842
rs78456138
rs7542079
rs8904
rs2021511
rs13153019
rs16903065
rs60600003
All
MR leave-one-out sensitivity analysis for 'Psoriasis' on 'Rheumatoid arthritis'

Figure S4. Visualization of the MR analysis of the effect of PsO on CD, UC, and RA. (A) Scatter plot of the MR analysis and Leave-one-out sensitivity analysis of the effect psoriasis on CD; (B) Scatter plot of the MR analysis and Leave-one-out sensitivity analysis of the effect of psoriasis on UC. (C) Scatter plot of the MR analysis and Leave-one-out sensitivity analysis of the effect of psoriasis on RA.

Abbreviations: MR, Mendelian randomization; PsO, psoriasis; CD, Crohn's disease; UC, Ulcerative colitis; RA, Rheumatoid arthritis.

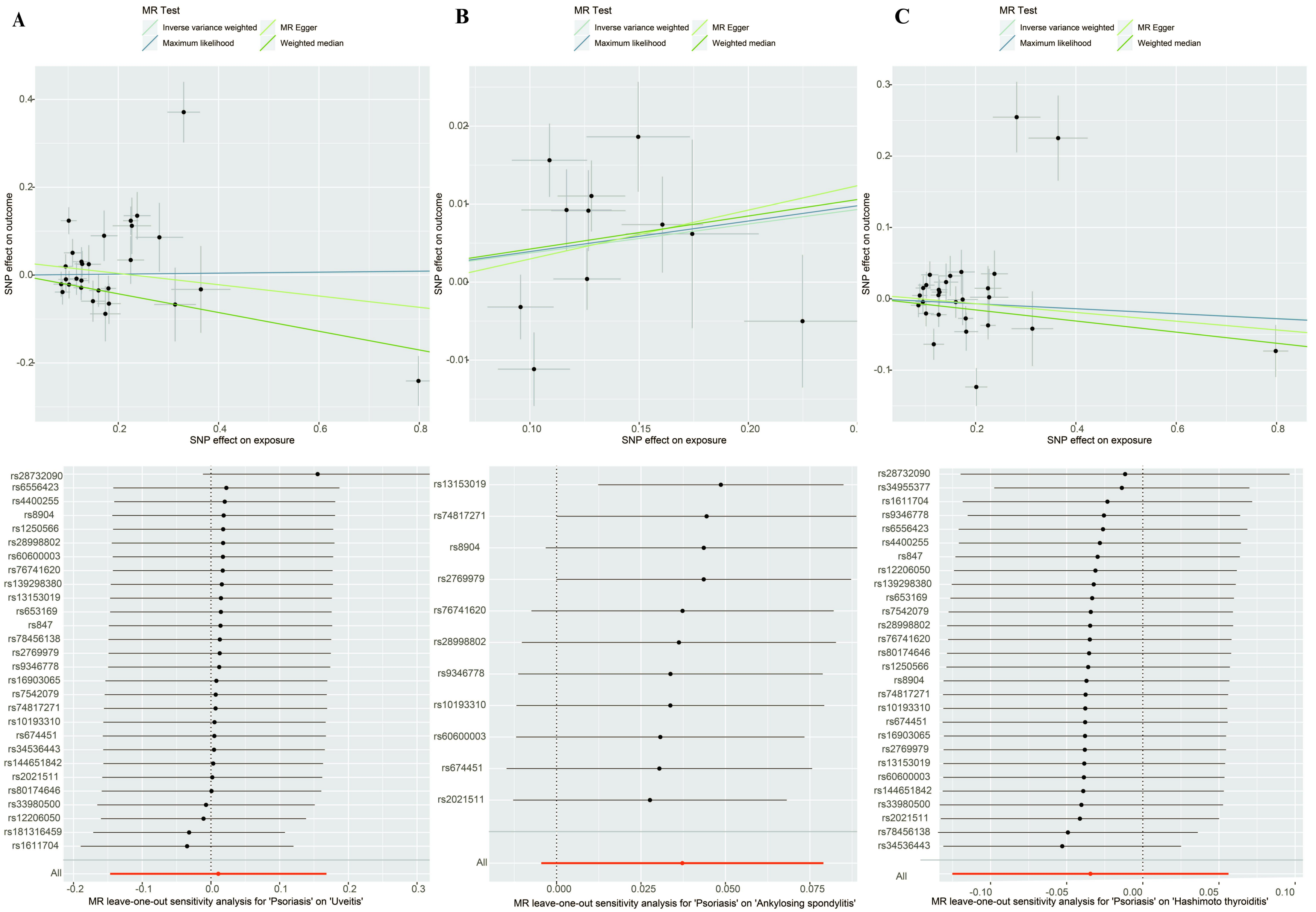
A
B
C
MR Test
Inverse variance weighted
MR Egger
Maximum likelihood
Weighted median
SNP effect on outcome
SNP effect on exposure
MR leave-one-out sensitivity analysis for 'Psoriasis' on 'Uveitis'
MR leave-one-out sensitivity analysis for 'Psoriasis' on 'Ankylosing spondylitis'
MR leave-one-out sensitivity analysis for 'Psoriasis' on 'Hashimoto thyroiditis'
rs28732090
rs6556423
rs4400255
rs8904
rs1250566
rs28998802
rs60600003
rs76741620
rs139298380
rs13153019
rs653169
rs847
rs78456138
rs2769979
rs9346778
rs16903065
rs7542079
rs74817271
rs10193310
rs674451
rs34536443
rs144651842
rs2021511
rs80174646
rs33980500
rs12206050
rs181316459
rs1611704
All
rs34955377
All

Figure S5. Visualization of the MR analysis of the effect of PsO on Uveitis, AS, and HT. (A) Scatter plot of the MR analysis and Leave-one-out sensitivity analysis of the effect psoriasis on Uveitis; (B) Scatter plot of the MR analysis and Leave-one-out sensitivity analysis of the effect of psoriasis on AS. (C) Scatter plot of the MR analysis and Leave-one-out sensitivity analysis of the effect of psoriasis on HT.

Abbreviations: MR, Mendelian randomization; PsO, psoriasis; AS, Ankylosing spondylitis; HT, Hashimoto thyroiditis.

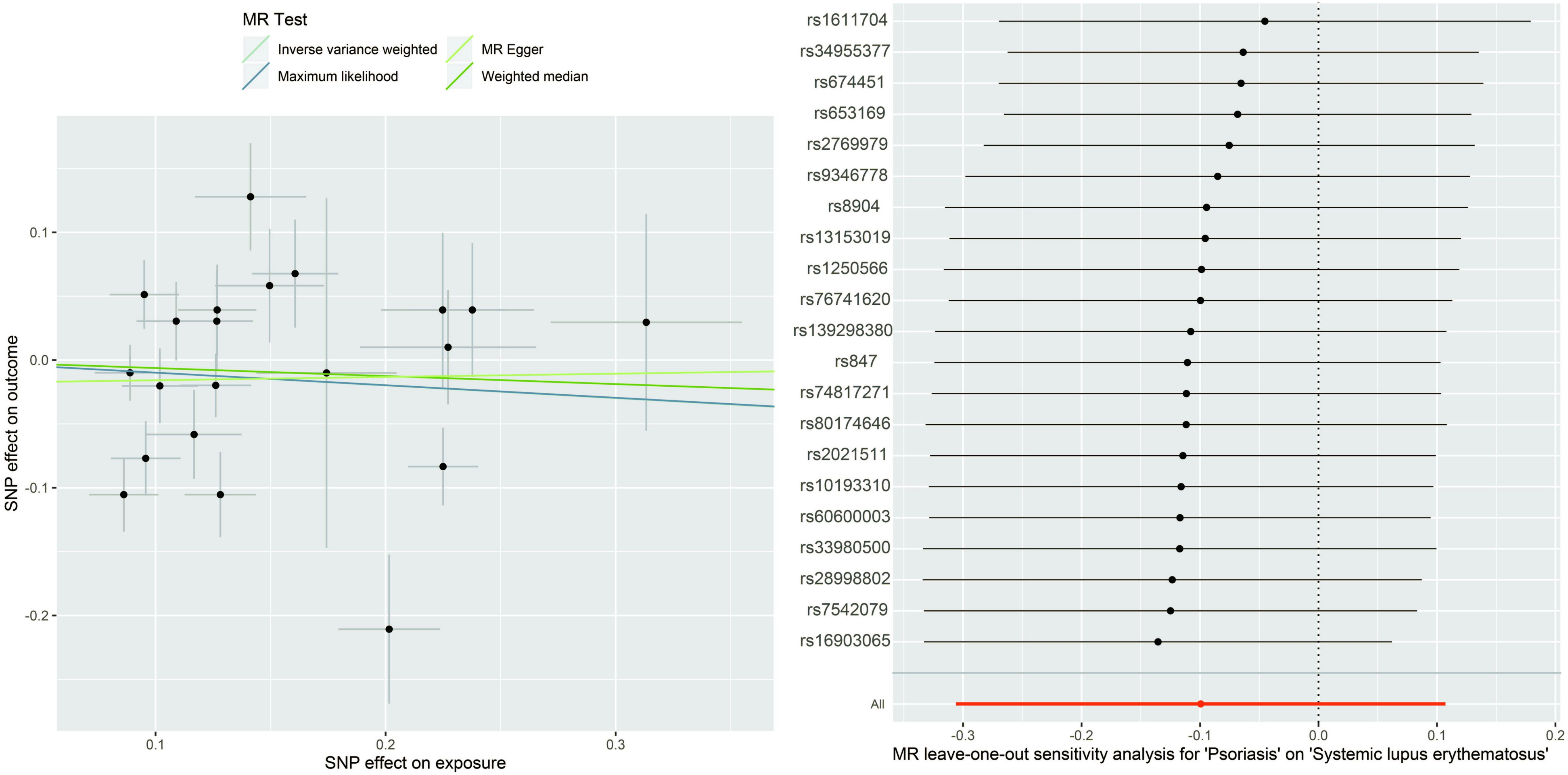

Figure S6. Visualization of the MR analysis of the effect of PsO on SLE. Scatter plot of the MR analysis and Leave-one-out sensitivity analysis of the effect psoriasis on SLE.

Abbreviations: MR, Mendelian randomization; PsO, psoriasis; SLE, Systemic lupus erythematosus.

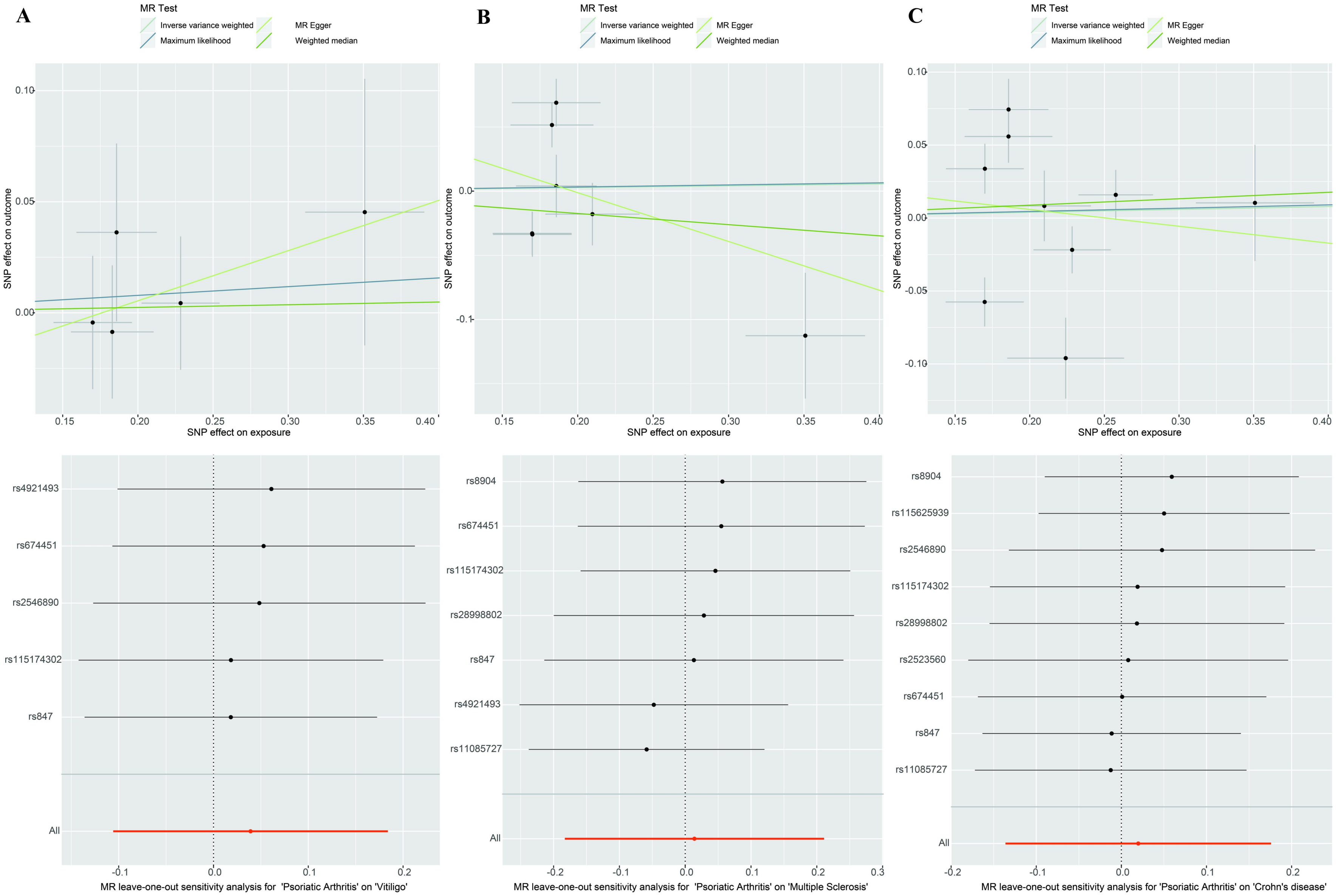
A
B
C
MR Test
Inverse variance weighted
Maximum likelihood
MR Egger
Weighted median
SNP effect on outcome
SNP effect on exposure
rs4921493
rs674451
rs2546890
rs115174302
rs847
All
MR leave-one-out sensitivity analysis for 'Psoriatic Arthritis' on 'Vitiligo'
rs8904
rs28998802
rs11085727
MR leave-one-out sensitivity analysis for 'Psoriatic Arthritis' on 'Multiple Sclerosis'
rs115625939
rs2523560
MR leave-one-out sensitivity analysis for 'Psoriatic Arthritis' on 'Crohn's disease'

Figure S7. Visualization of the MR analysis of the effect of PsA on Vitiligo, MS, and CD. (A) Scatter plot of the MR analysis and Leave-one-out sensitivity analysis of the effect PsA on vitiligo; (B) Scatter plot of the MR analysis and Leave-one-out sensitivity analysis of the effect of PsA on MS. (C) Scatter plot of the MR analysis and Leave-one-out sensitivity analysis of the effect of psoriasis on CD.

Abbreviations: MR, Mendelian randomization; PsA, Psoriatic arthritis; MS, Multiple sclerosis; CD, Crohn's disease.

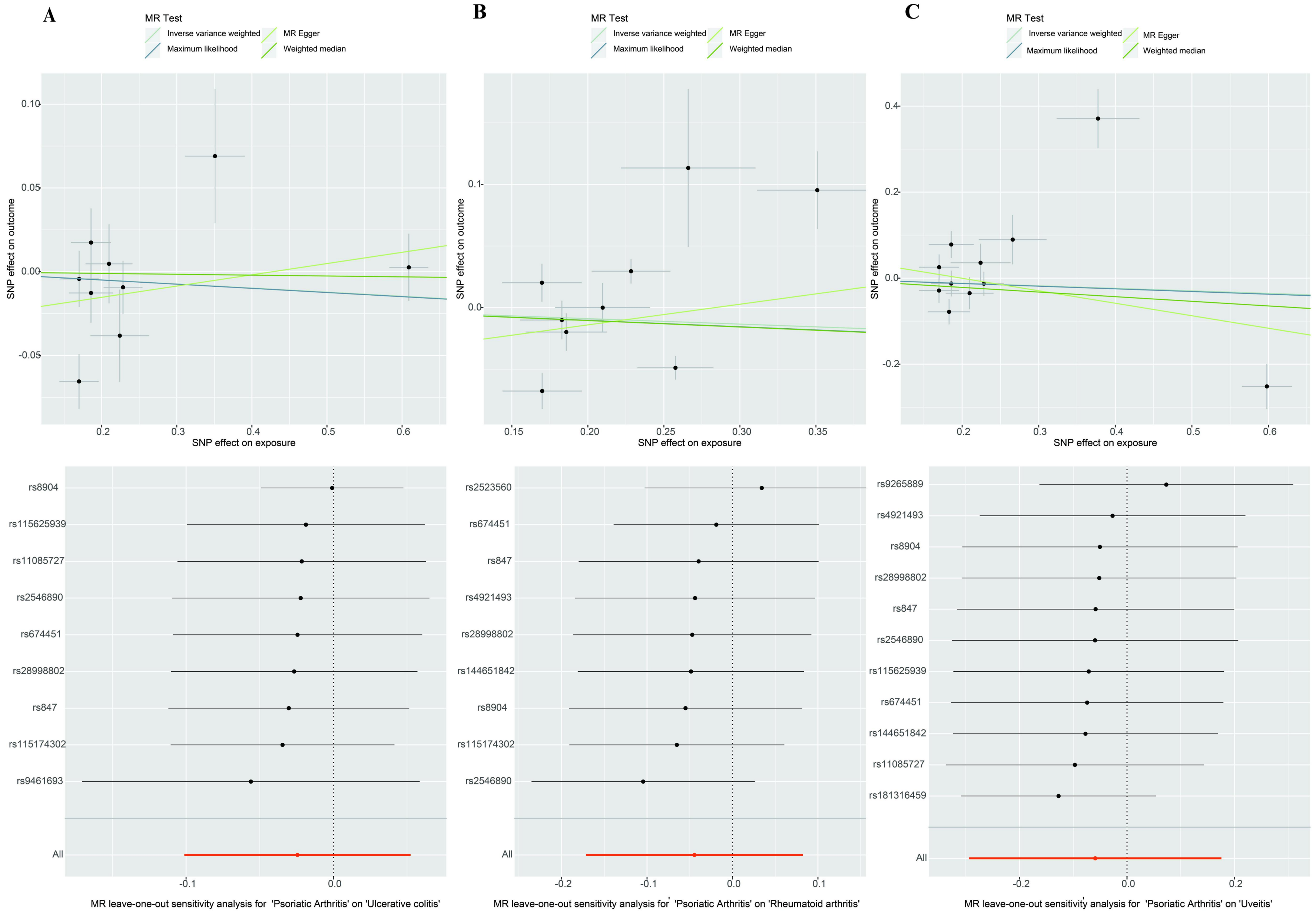
A
B
C
MR Test
Inverse variance weighted
MR Egger
Maximum likelihood
Weighted median
SNP effect on outcome
SNP effect on exposure
rs8904
rs115625939
rs11085727
rs2546890
rs674451
rs28998802
rs847
rs115174302
rs9461693
All
rs2523560
rs4921493
rs144651842
rs9265889
rs181316459
MR leave-one-out sensitivity analysis for 'Psoriatic Arthritis' on 'Ulcerative colitis'
MR leave-one-out sensitivity analysis for 'Psoriatic Arthritis' on 'Rheumatoid arthritis'
MR leave-one-out sensitivity analysis for 'Psoriatic Arthritis' on 'Uveitis'

Figure S8. Visualization of the MR analysis of the effect of PsA on UC, RA, and Uveitis. (A) Scatter plot of the MR analysis and Leave-one-out sensitivity analysis of the effect PsA on UC; (B) Scatter plot of the MR analysis and Leave-one-out sensitivity analysis of the effect of PsA on RA. (C) Scatter plot of the MR analysis and Leave-one-out sensitivity analysis of the effect of PsA on Uveitis.

Abbreviations: MR, Mendelian randomization; PsA, Psoriatic arthritis; UC, Ulcerative colitis; RA, Rheumatoid arthritis.

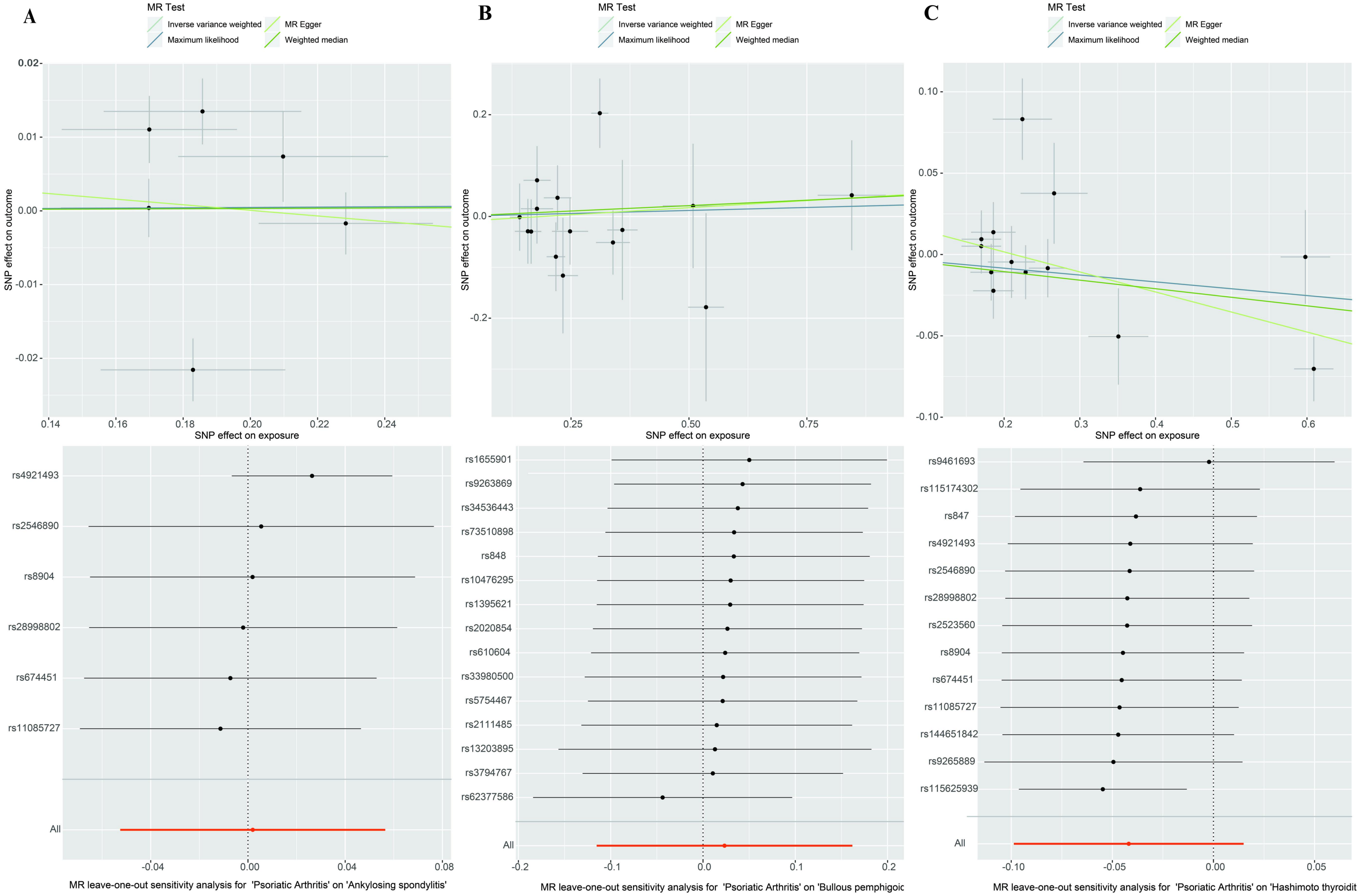

A
B
C
MR Test
Inverse variance weighted
MR Egger
Maximum likelihood
Weighted median
SNP effect on outcome
SNP effect on exposure
rs4921493
rs2546890
rs8904
rs28998802
rs674451
rs11085727
All
MR leave-one-out sensitivity analysis for 'Psoriatic Arthritis' on 'Ankylosing spondylitis'
rs1655901
rs9263869
rs34536443
rs73510898
rs848
rs10476295
rs1395621
rs2020854
rs610604
rs33980500
rs5754467
rs2111485
rs13203895
rs3794767
rs62377586
All
MR leave-one-out sensitivity analysis for 'Psoriatic Arthritis' on 'Bullous pemphigoic
rs9461693
rs115174302
rs847
rs4921493
rs2546890
rs28998802
rs2523560
rs8904
rs674451
rs11085727
rs144651842
rs9265889
rs115625939
All
MR leave-one-out sensitivity analysis for 'Psoriatic Arthritis' on 'Hashimoto thyroidit

Figure S9. Visualization of the MR analysis of the effect of PsA on AS, BP, and HT. (A) Scatter plot of the MR analysis and Leave-one-out sensitivity analysis of the effect PsA on AS; (B) Scatter plot of the MR analysis and Leave-one-out sensitivity analysis of the effect of PsA on BP. (C) Scatter plot of the MR analysis and Leave-one-out sensitivity analysis of the effect of PsA on HT.

Abbreviations: MR, Mendelian randomization; PsA, Psoriatic arthritis; AS, Ankylosing spondylitis; BP, Bullous pemphigoid; HT, Hashimoto thyroiditis.

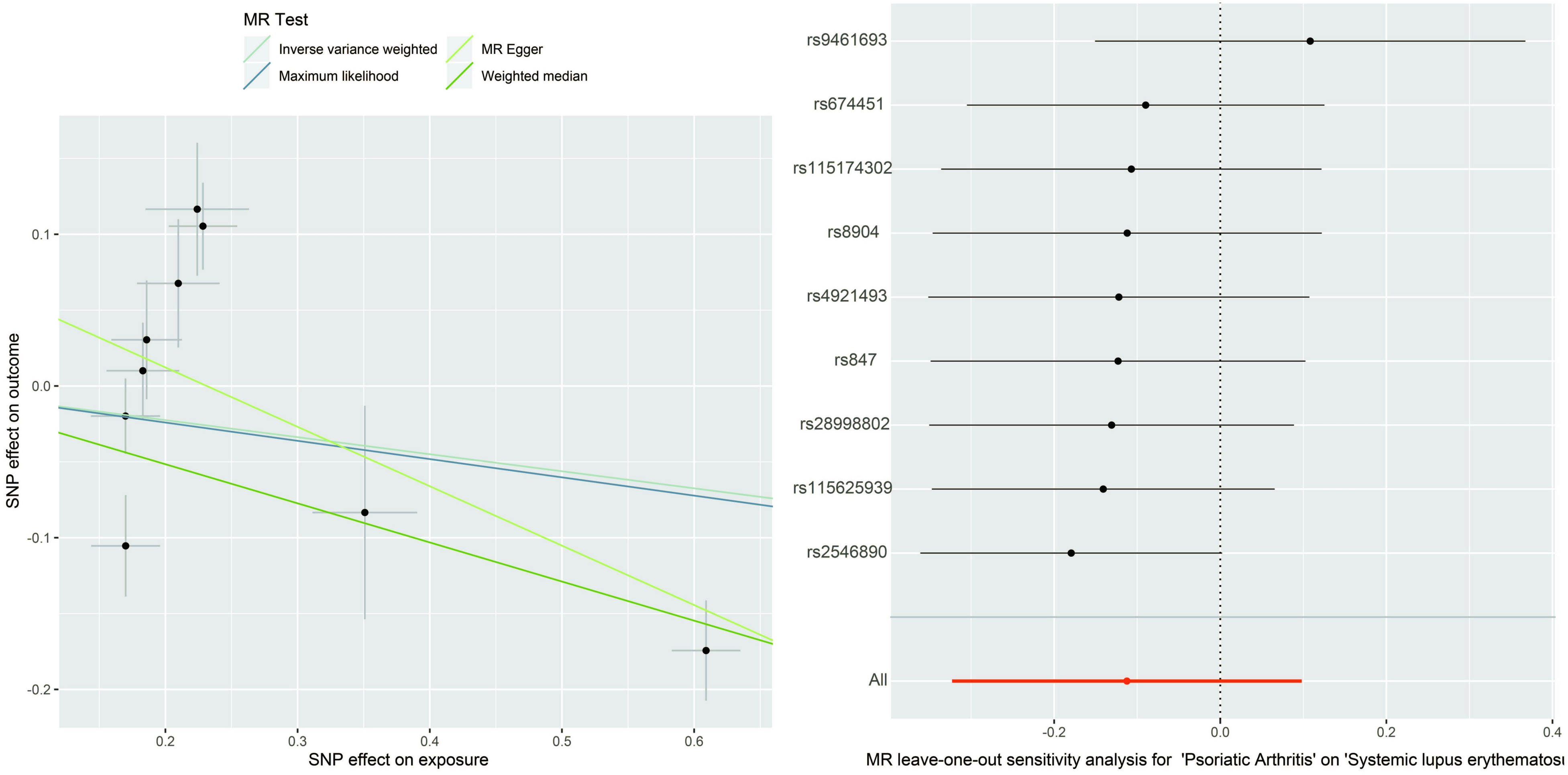

Figure S10. Visualization of the MR analysis of the effect of PsA on SLE. Scatter plot of the MR analysis and Leave-one-out sensitivity analysis of the effect PsA on SLE;

Abbreviations: MR, Mendelian randomization; PsA, Psoriatic arthritis; SLE, Systemic lupus erythematosus.

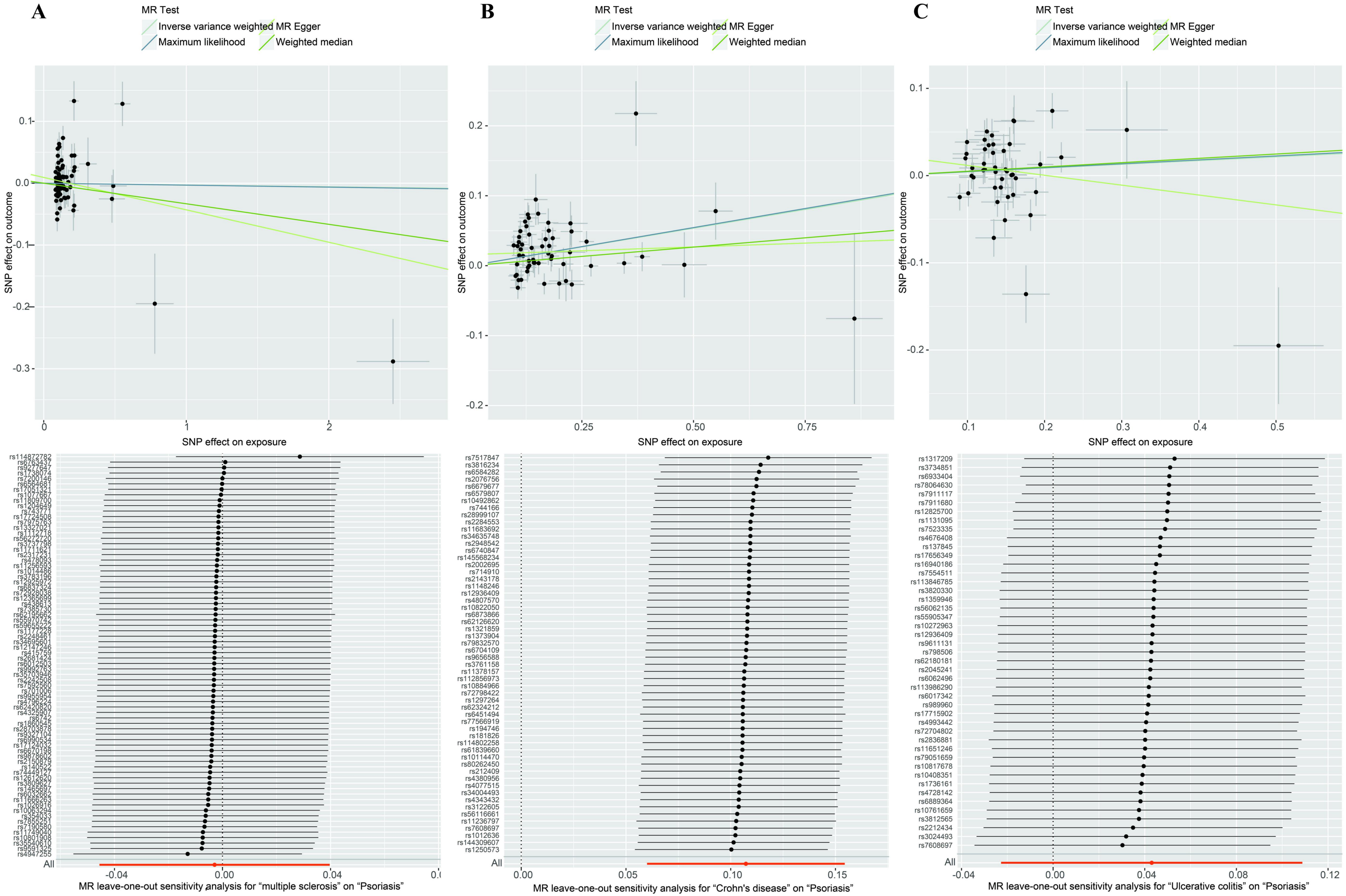
A
B
C
MR Test
Inverse variance weighted
MR Egger
Maximum likelihood
Weighted median
SNP effect on outcome
SNP effect on exposure
MR leave-one-out sensitivity analysis for “multiple sclerosis” on “Psoriasis”
MR leave-one-out sensitivity analysis for “Crohn's disease” on “Psoriasis”
MR leave-one-out sensitivity analysis for “Ulcerative colitis” on “Psoriasis”
All

Figure S11. Visualization of the MR analysis of the effect of MS, CD, and UC on PsO . (A) Scatter plot of the MR analysis and Leave-one-out sensitivity analysis of the effect MS on PsO; (B) Scatter plot of the MR analysis and Leave-one-out sensitivity analysis of the effect of CD on PsO; (C) Scatter plot of the MR analysis and Leave-one-out sensitivity analysis of the effect of UC on PsO.

Abbreviations: MR, Mendelian randomization; PsO, psoriasis; MS, multiple sclerosis; CD, Crohn's disease; UC, Ulcerative colitis.

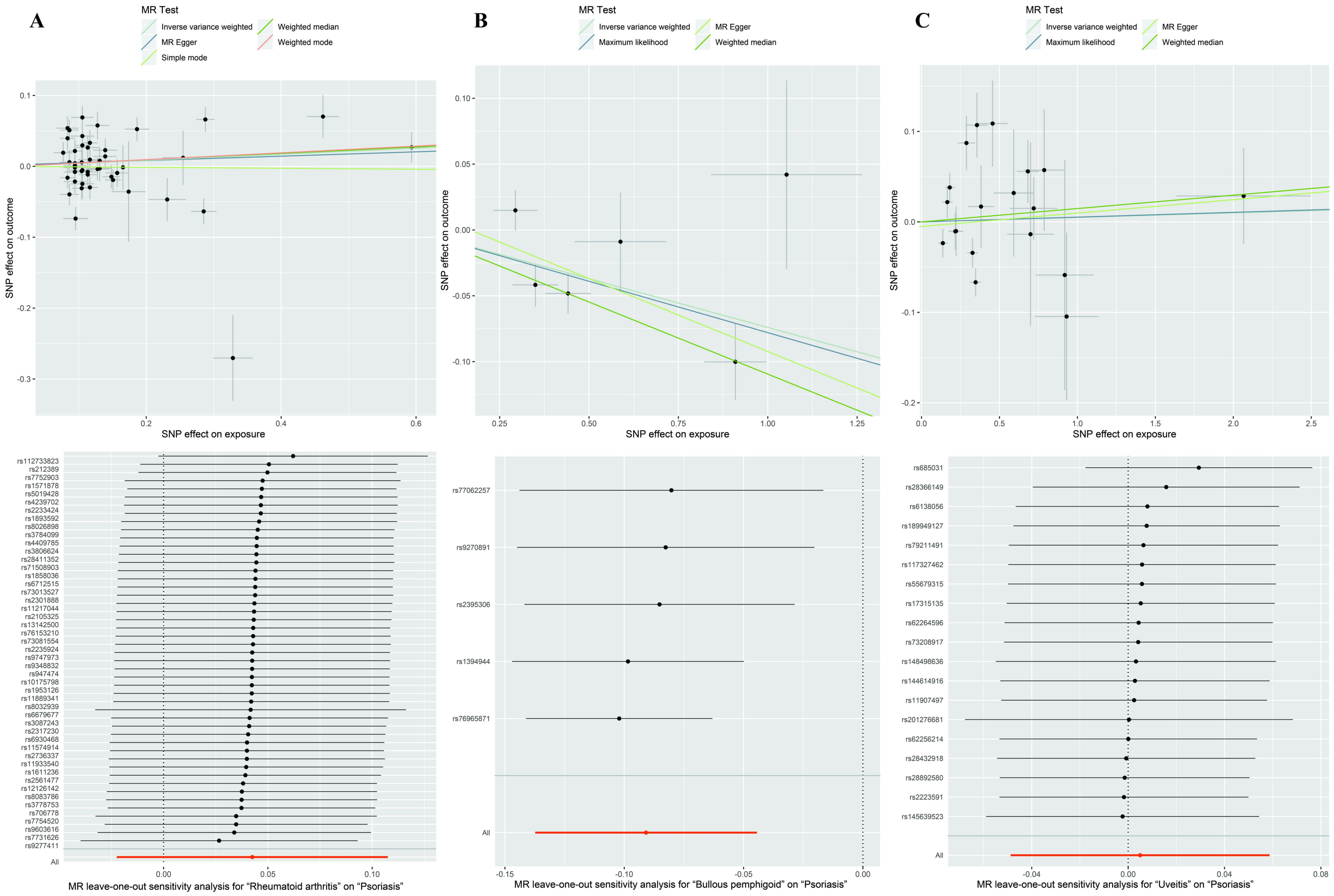

A
MR Test
Inverse variance weighted
MR Egger
Simple mode
Weighted median
Weighted mode
SNP effect on outcome
SNP effect on exposure
0.1
0.0
-0.1
-0.2
-0.3
0.2
0.4
0.6
rs112733823
rs212389
rs7752903
rs1571878
rs5019428
rs4239702
rs2233424
rs1893592
rs8026898
rs3784099
rs4409785
rs3806624
rs28411352
rs71508903
rs1858036
rs6712515
rs73013527
rs2301888
rs11217044
rs2105325
rs13142500
rs76153210
rs73081554
rs2235924
rs9747973
rs9348832
rs947474
rs10175798
rs1953126
rs11889341
rs8032939
rs6679677
rs3087243
rs2317230
rs6930468
rs11574914
rs2736337
rs11933540
rs1611236
rs2561477
rs12126142
rs8083786
rs3778753
rs706778
rs7754520
rs9603616
rs7731626
rs9277411
All
0.00
0.05
0.10
MR leave-one-out sensitivity analysis for “Rheumatoid arthritis” on “Psoriasis”
B
MR Test
Inverse variance weighted
Maximum likelihood
MR Egger
Weighted median
SNP effect on outcome
SNP effect on exposure
0.10
0.05
0.00
-0.05
-0.10
0.25
0.50
0.75
1.00
1.25
rs77062257
rs9270891
rs2395306
rs1394944
rs76965871
All
-0.15
-0.10
-0.05
0.00
MR leave-one-out sensitivity analysis for “Bullous pemphigoid” on “Psoriasis”
C
MR Test
Inverse variance weighted
Maximum likelihood
MR Egger
Weighted median
SNP effect on outcome
SNP effect on exposure
0.1
0.0
-0.1
-0.2
0.0
0.5
1.0
1.5
2.0
2.5
rs685031
rs28366149
rs6138056
rs189949127
rs79211491
rs117327462
rs55679315
rs17315135
rs62264596
rs73208917
rs148498636
rs144614916
rs11907497
rs201276681
rs62256214
rs28432918
rs28892580
rs2223591
rs145639523
All
-0.04
0.00
0.04
0.08
MR leave-one-out sensitivity analysis for “Uveitis” on “Psoriasis”

Figure S12. Visualization of the MR analysis of the effect of RA, BP, and Uveitis on PsO . (A) Scatter plot of the MR analysis and Leave-one-out sensitivity analysis of the effect RA on PsO; (B) Scatter plot of the MR analysis and Leave-one-out sensitivity analysis of the effect of BP on PsO; (C) Scatter plot of the MR analysis and Leave-one-out sensitivity analysis of the effect of Uveitis on PsO.

Abbreviations: MR, Mendelian randomization; PsO, psoriasis; RA, Rheumatoid arthritis; BP, Bullous pemphigoid.

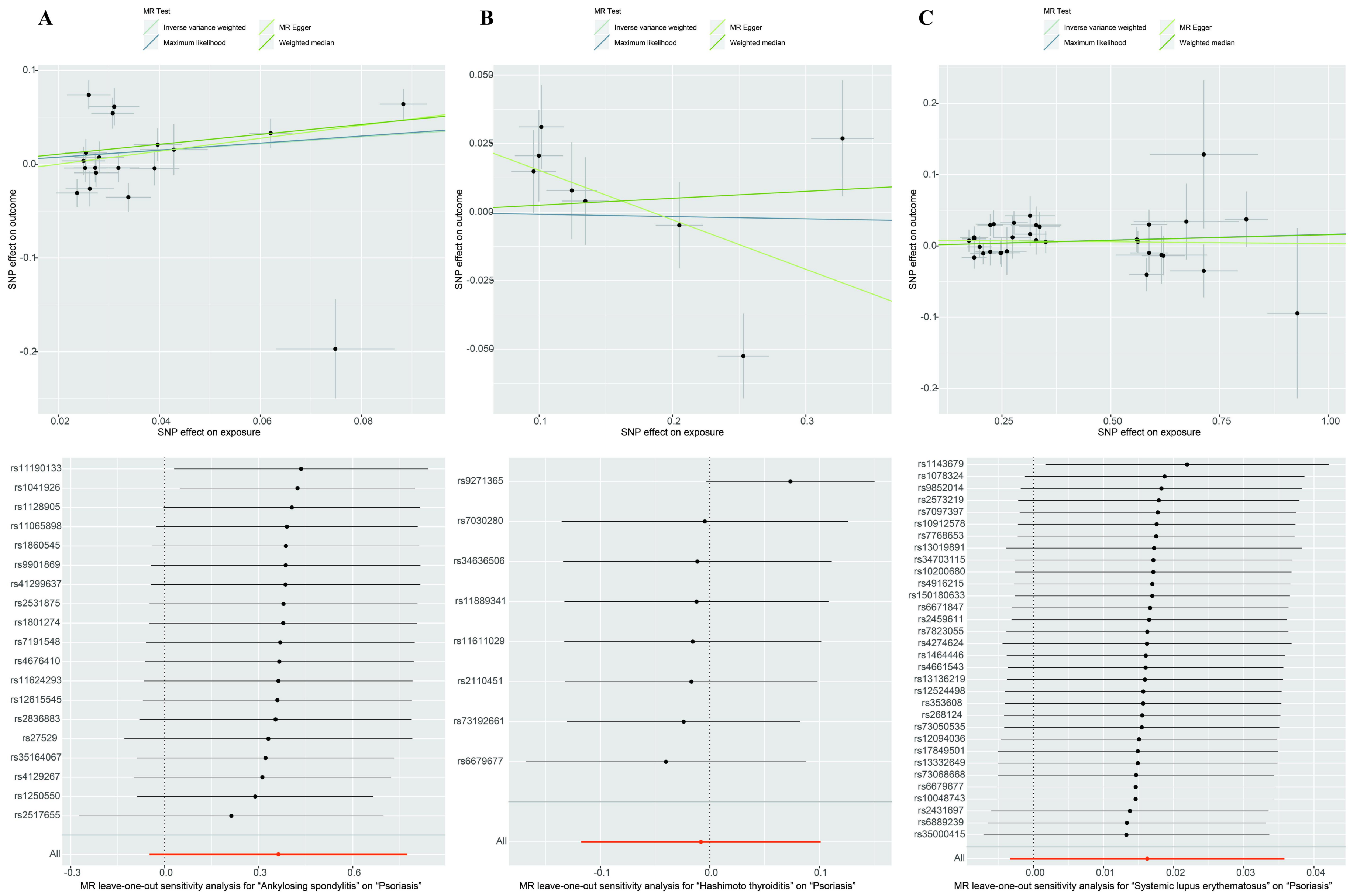
A
MR Test
Inverse variance weighted
Maximum likelihood
MR Egger
Weighted median
SNP effect on outcome
SNP effect on exposure
B
MR Test
Inverse variance weighted
Maximum likelihood
MR Egger
Weighted median
SNP effect on outcome
SNP effect on exposure
C
MR Test
Inverse variance weighted
Maximum likelihood
MR Egger
Weighted median
SNP effect on outcome
SNP effect on exposure
rs11190133
rs1041926
rs1128905
rs11065898
rs1860545
rs9901869
rs41299637
rs2531875
rs1801274
rs7191548
rs4676410
rs11624293
rs12615545
rs2836883
rs27529
rs35164067
rs4129267
rs1250550
rs2517655
All
MR leave-one-out sensitivity analysis for "Ankylosing spondylitis" on "Psoriasis"
rs9271365
rs7030280
rs34636506
rs11889341
rs11611029
rs2110451
rs73192661
rs6679677
All
MR leave-one-out sensitivity analysis for "Hashimoto thyroiditis" on "Psoriasis"
rs1143679
rs1078324
rs9852014
rs2573219
rs7097397
rs10912578
rs7768653
rs13019891
rs34703115
rs10200680
rs4916215
rs150180633
rs6671847
rs2459611
rs7823055
rs4274624
rs1464446
rs4661543
rs13136219
rs12524498
rs353608
rs268124
rs73050535
rs12094036
rs17849501
rs13332649
rs73068668
rs6679677
rs10048743
rs2431697
rs6889239
rs35000415
All
MR leave-one-out sensitivity analysis for "Systemic lupus erythematosus" on "Psoriasis"

Figure S13. Visualization of the MR analysis of the effect of AS, HT, and SLE on PsO . (A) Scatter plot of the MR analysis and Leave-one-out sensitivity analysis of the effect AS on PsO; (B) Scatter plot of the MR analysis and Leave-one-out sensitivity analysis of the effect of HT on PsO; (C) Scatter plot of the MR analysis and Leave-one-out sensitivity analysis of the effect of SLE on PsO.

Abbreviations: MR, Mendelian randomization; PsO, psoriasis; AS, Ankylosing spondylitis; HT, Hashimoto thyroiditis, SLE, Systemic lupus erythematosus.

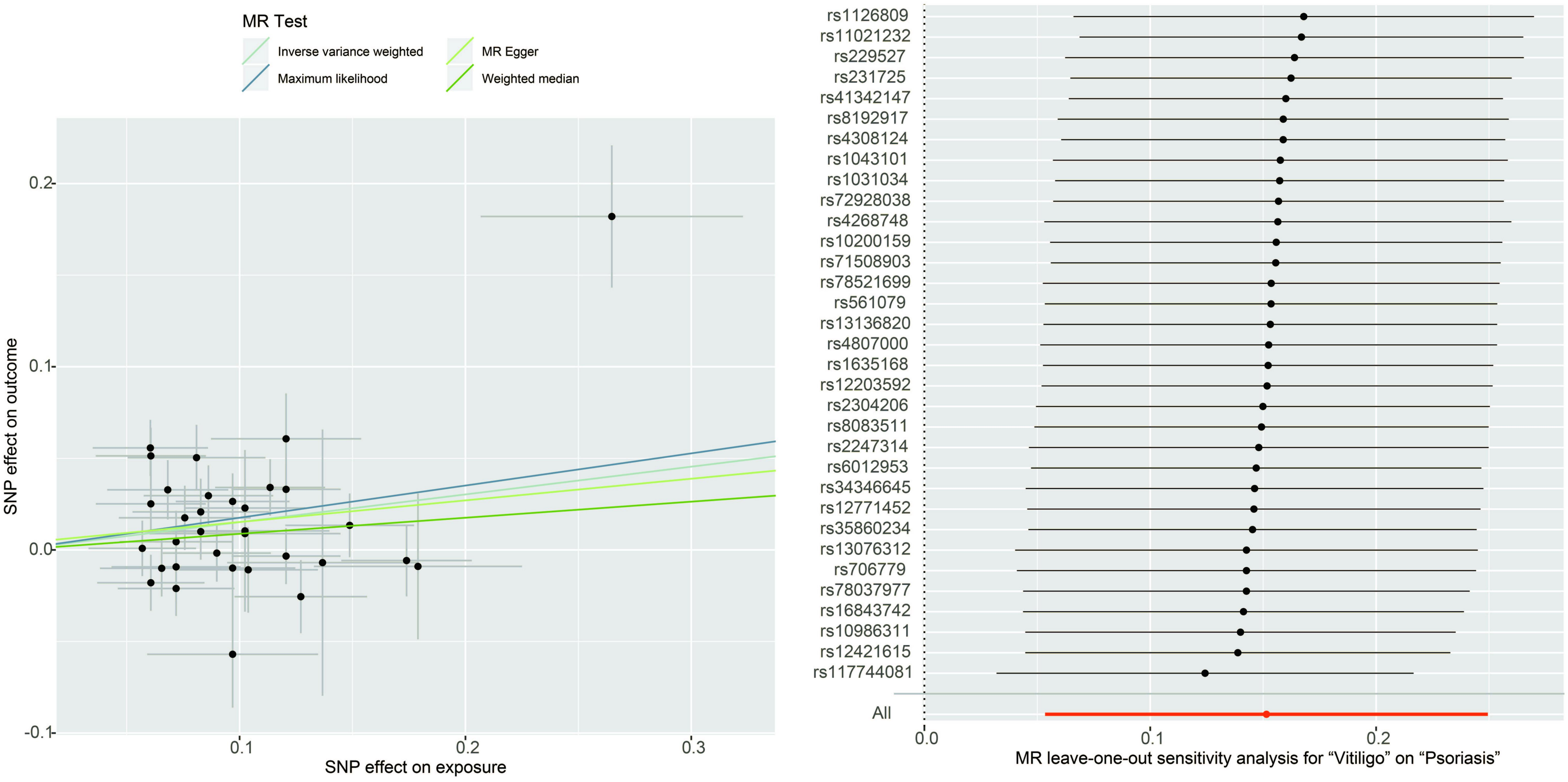

Figure S14. Visualization of the MR analysis of the effect of Vitiligo on PsO . Scatter plot of the MR analysis and Leave-one-out sensitivity analysis of the effect Vitiligo on PsO.

Abbreviations: MR, Mendelian randomization; PsO, psoriasis.

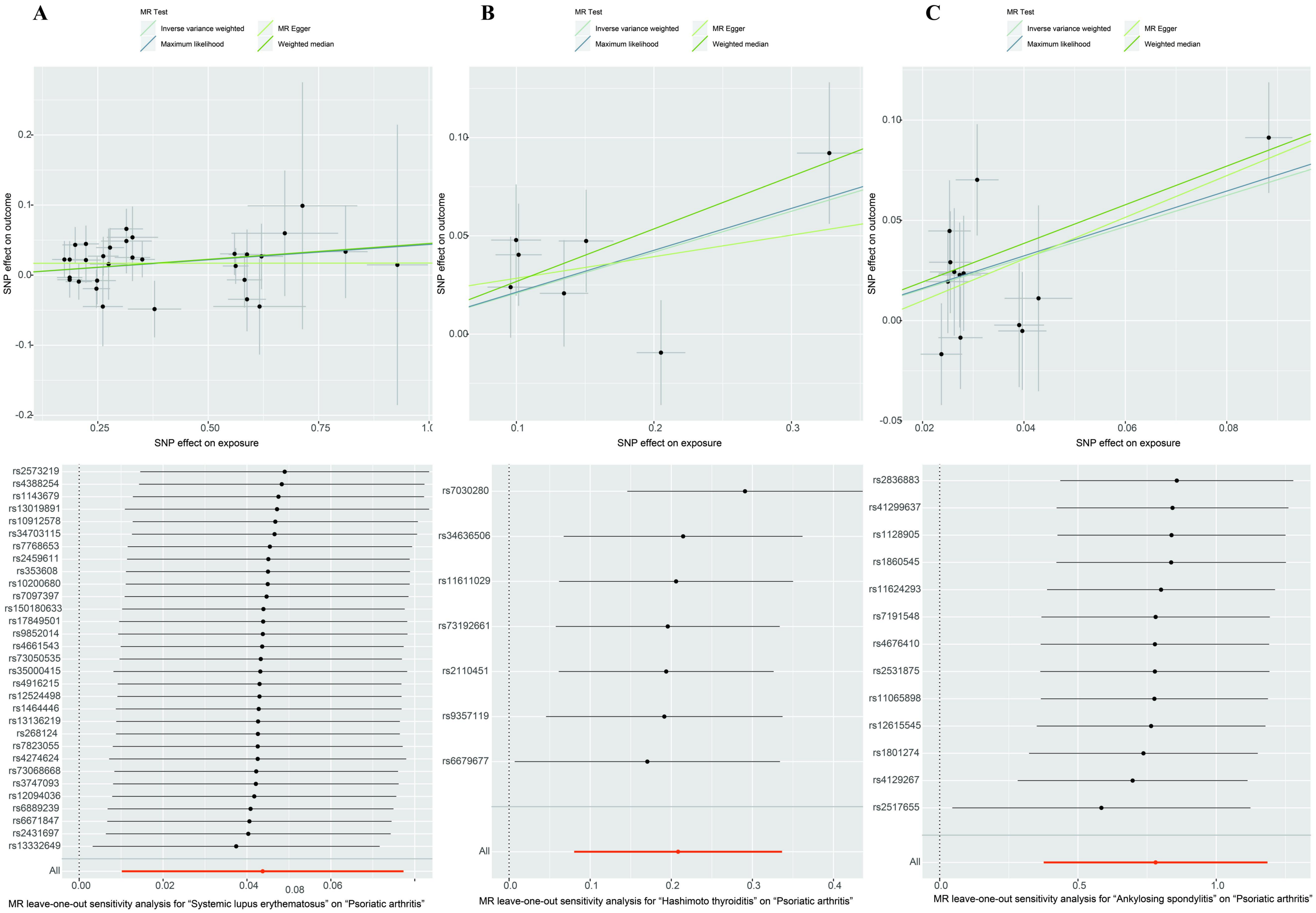
A
MR Test
Inverse variance weighted
Maximum likelihood
MR Egger
Weighted median
SNP effect on outcome
SNP effect on exposure
MR leave-one-out sensitivity analysis for "Systemic lupus erythematosus" on "Psoriatic arthritis"
rs2573219
rs4388254
rs1143679
rs13019891
rs10912578
rs34703115
rs7768653
rs2459611
rs353608
rs10200680
rs7097397
rs150180633
rs17849501
rs9852014
rs4661543
rs73050535
rs35000415
rs4916215
rs12524498
rs1464446
rs13136219
rs268124
rs7823055
rs4274624
rs73068668
rs3747093
rs12094036
rs6889239
rs6671847
rs2431697
rs13332649
All
B
MR Test
Inverse variance weighted
Maximum likelihood
MR Egger
Weighted median
SNP effect on outcome
SNP effect on exposure
MR leave-one-out sensitivity analysis for "Hashimoto thyroiditis" on "Psoriatic arthritis"
rs7030280
rs34636506
rs11611029
rs73192661
rs2110451
rs9357119
rs6679677
All
C
MR Test
Inverse variance weighted
Maximum likelihood
MR Egger
Weighted median
SNP effect on outcome
SNP effect on exposure
MR leave-one-out sensitivity analysis for "Ankylosing spondylitis" on "Psoriatic arthritis"
rs2836883
rs41299637
rs1128905
rs1860545
rs11624293
rs7191548
rs4676410
rs2531875
rs11065898
rs12615545
rs1801274
rs4129267
rs2517655
All

Figure S15. Visualization of the MR analysis of the effect of SLE, HT, and AS on PsA . (A) Scatter plot of the MR analysis and Leave-one-out sensitivity analysis of the effect SLE on PsA; (B) Scatter plot of the MR analysis and Leave-one-out sensitivity analysis of the effect of HT on PsA; (C) Scatter plot of the MR analysis and Leave-one-out sensitivity analysis of the effect of AS on PsA.

Abbreviations: MR, Mendelian randomization; PsA, psoriatic arthritis; SLE, Systemic lupus erythematosus; HT, Hashimoto thyroiditis; AS, Ankylosing spondylitis.

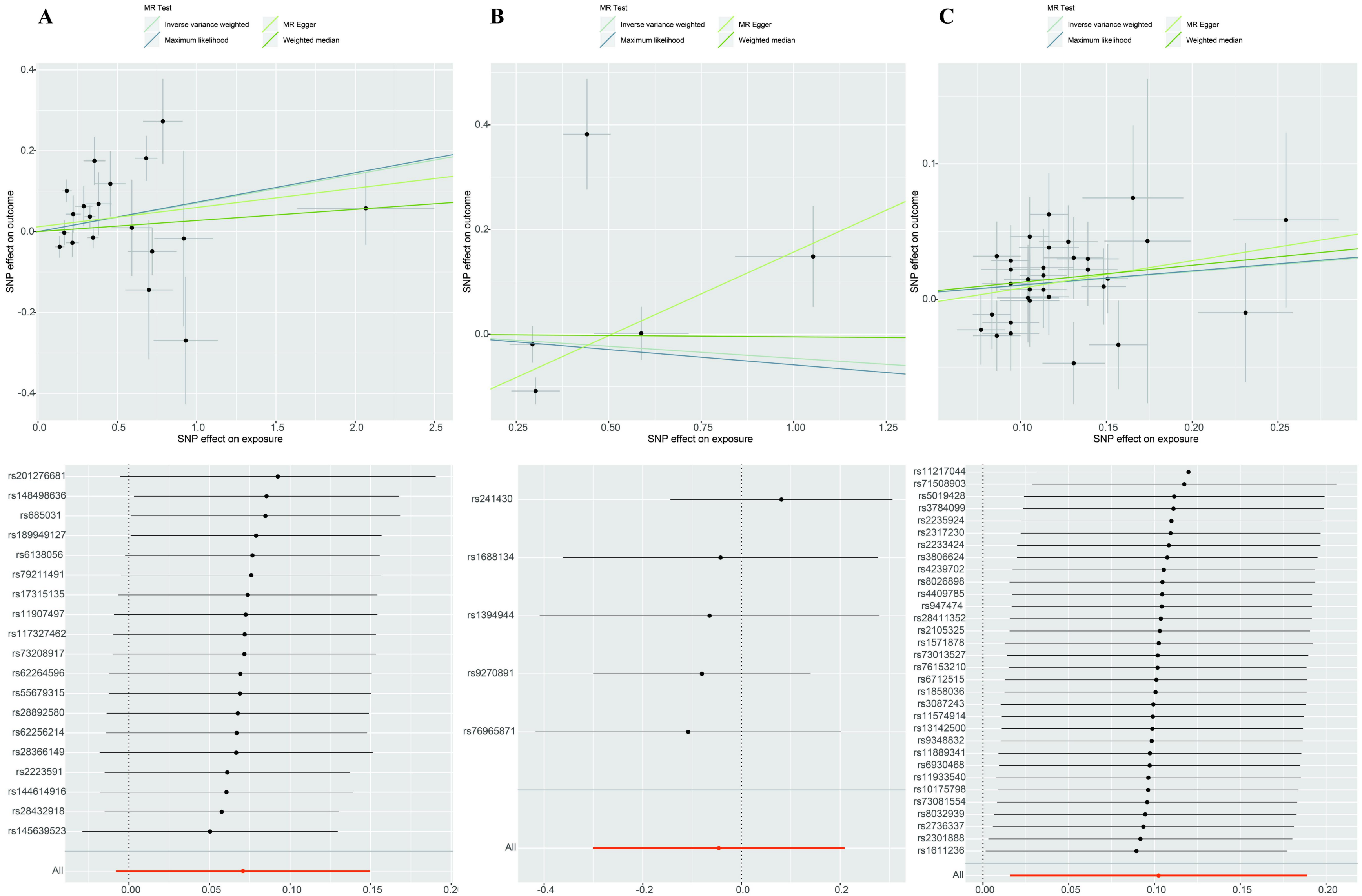

MR leave-one-out sensitivity analysis for “Uveitis” on “Psoriatic arthritis”

MR leave-one-out sensitivity analysis for “Bullous pemphigoid” on “Psoriatic arthritis”

MR leave-one-out sensitivity analysis for “Rheumatoid arthritis” on “Psoriatic arthritis”

Figure S16. Visualization of the MR analysis of the effect of Uveitis, BP, and RA on PsA . (A) Scatter plot of the MR analysis and Leave-one-out sensitivity analysis of the effect Uveitis on PsA; (B) Scatter plot of the MR analysis and Leave-one-out sensitivity analysis of the effect of BP on PsA; (C) Scatter plot of the MR analysis and Leave-one-out sensitivity analysis of the effect of RA on PsA.

Abbreviations: MR, Mendelian randomization; PsA, psoriatic arthritis; BP, Bullous pemphigoid; RA, Rheumatoid arthritis.

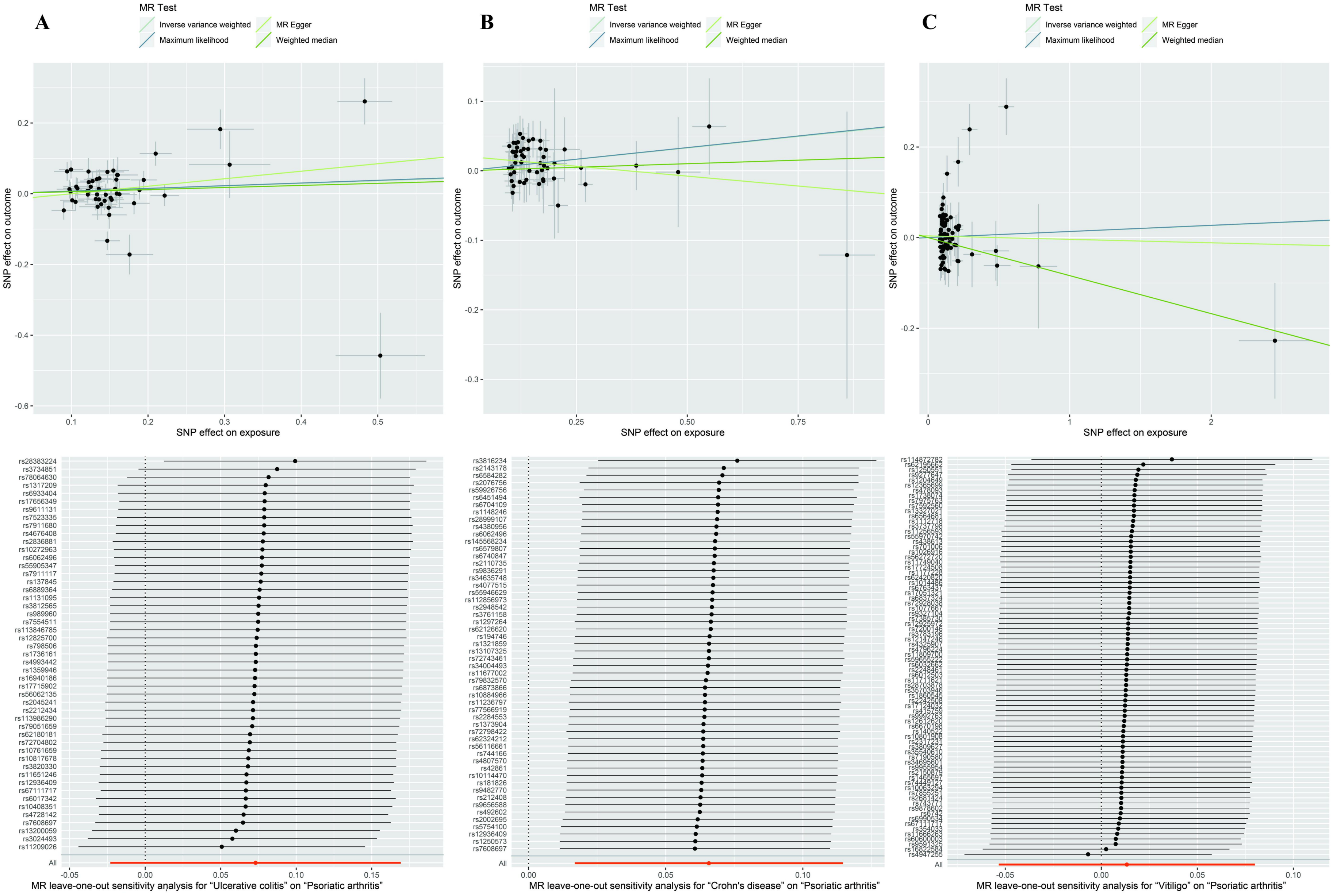

A
B
C
MR Test
Inverse variance weighted
MR Egger
Maximum likelihood
Weighted median
SNP effect on outcome
SNP effect on exposure
MR leave-one-out sensitivity analysis for "Ulcerative colitis" on "Psoriatic arthritis"
MR leave-one-out sensitivity analysis for "Crohn's disease" on "Psoriatic arthritis"
MR leave-one-out sensitivity analysis for "Vitiligo" on "Psoriatic arthritis"
All

Figure S17. Visualization of the MR analysis of the effect of UC, CD, and MS on PsA . (A) Scatter plot of the MR analysis and Leave-one-out sensitivity analysis of the effect UC on PsA; (B) Scatter plot of the MR analysis and Leave-one-out sensitivity analysis of the effect of CD on PsA; (C) Scatter plot of the MR analysis and Leave-one-out sensitivity analysis of the effect of MS on PsA.

Abbreviations: MR, Mendelian randomization; PsA, psoriatic arthritis; CD, Crohn's disease; UC, Ulcerative colitis; MS: multiple sclerosis.

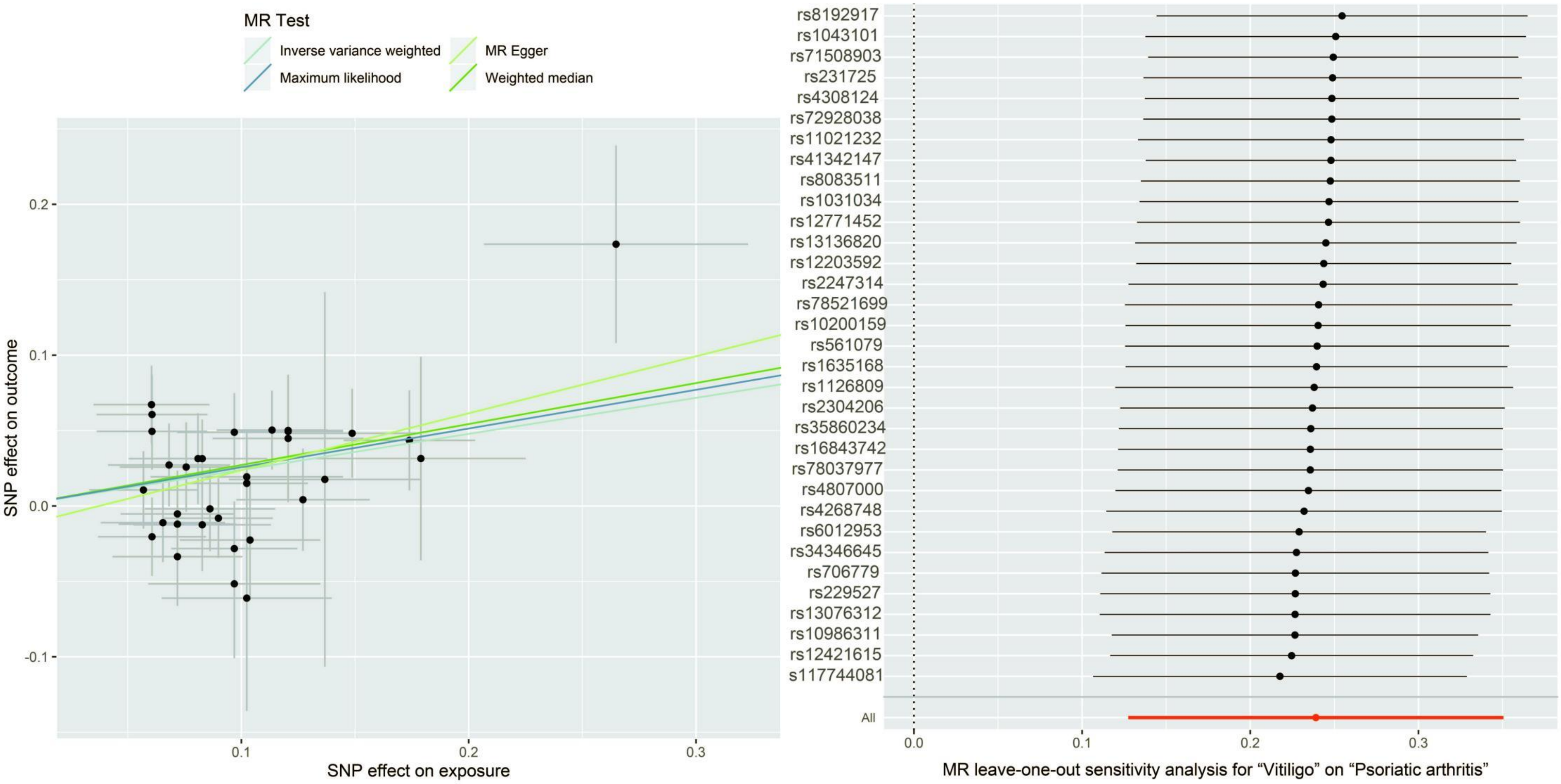

Figure S18. Visualization of the MR analysis of the effect of Vitiligo on PsA . Scatter plot of the MR analysis and Leave-one-out sensitivity analysis of the effect Vitiligo on PsA;

Abbreviations: MR, Mendelian randomization; PsA, psoriatic arthritis.

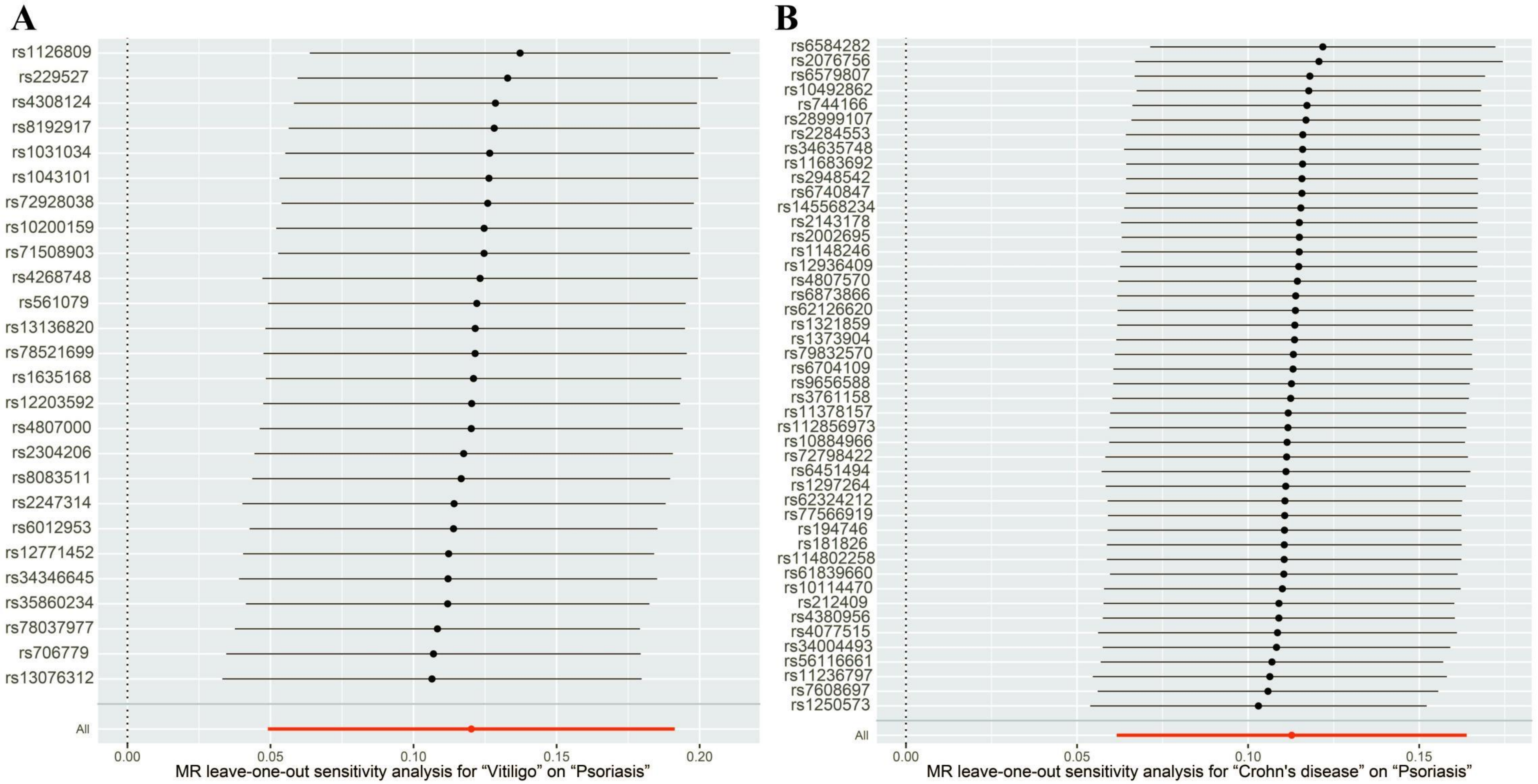

Figure S19. Visualization of the MR analysis of the effect of Vitiligo and CD on PsO after reducing heterogeneiy. (A) Leave-one-out sensitivity analysis of the effect Vitiligo on PsO. (B) Leave-one-out sensitivity analysis of the effect CD on PsO.

Abbreviations: MR, Mendelian randomization; PsO, psoriasis; CD, Crohn's disease.

Table S1. Detail information of traits involved in this study.

Phenotype	Study	Ancestry	nCase	nControl	Sample size	GWAS ID
PsO	FinnGen data	European	9,267	364,071	373,338	/
	Philip E Stuart et al PubMed ID:34927100	European	15,967	28,194	44,161	ebi-a-GCST90019017
PsA	FinnGen data	European	3,186	240,862	244,048	/
	Soomro M et al	European	5,065	21,286	26,351	/
SLE	James Bentham et al PubMed ID:26502338	European	5,201	9,066	14,267	ebi-a-GCST003156
MS	Nikolaos A. Patsopoulos et al PubMed ID: 31604244	European	47,429	68,374	115,803	ieu-b-18
RA	Okada Y et al PubMed ID: 24390342	European	14,361	42,923	57,284	ebi-a-GCST002318
Uveitis	Sakaue S et al PubMed ID: 34594039	European	2,616	478,126	480,742	ebi-a-GCST90018938
CD	Katrina M de Lange et al PubMed ID: 28067908	European	12,194	28,072	40,266	ebi-a-GCST004132
UC	Katrina M de Lange et al PubMed ID:28067908	European	12,366	33,609	45,975	ebi-a-GCST004133
Vitiligo	Jin Y et al	European	4,680	39,586	44,266	/
BP	FinnGen data	European	507	375,767	376,274	/
AS	Adrian Cortes et al PubMed ID: 23749187	European	9,069	13,578	22,647	ebi-a-GCST005529
HT	Saori Sakaue et al PubMed ID: 34594039	European	15,654	379,986	395,640	ebi-a-GCST90018855

Abbreviations: PsO, psoriasis; PsA, psoriatic arthritis; SLE, Systemic lupus erythematosus; MS, multiple sclerosis; RA, Rheumatoid arthritis; CD, Crohn's disease; UC, Ulcerative colitis; BP, Bullous pemphigoid; AS, Ankylosing spondylitis; HT, Hashimoto thyroiditis.

Table S2. The causal effects of PsO on AIDs with heterogeneity and pleiotropy test results.

Exposure	Outcomes	No.SNPs	MR methods	P	OR	95% LCI	95%UCI	Heterogeneiyt(I^2)	Egger-intercept P-value
PsO	SLE	21	MR Egger	0.935	1.03	0.56	1.90	72.77	0.68
			Weighted Median	0.519	0.94	0.78	1.14		
			Inverse variance weighted	0.346	0.91	0.74	1.11		
			Maximum likelihood	0.085	0.91	0.81	1.01		
	MS	19	MR Egger	0.722	1.14	0.56	2.31	135.21	0.84
			Weighted Median	0.927	0.99	0.87	1.13		
			Inverse variance weighted	0.556	1.06	0.87	1.29		
			Maximum likelihood	0.105	1.07	0.99	1.15		
	CD	17	MR Egger	0.340	1.25	0.80	1.96	82.89	0.45
			Weighted Median	0.694	1.03	0.90	1.17		
			Inverse variance weighted	0.466	1.06	0.90	1.25		
			Maximum likelihood	0.083	1.07	0.99	1.15		
	UC	20	MR Egger	0.732	1.10	0.66	1.83	124.65	0.89
			Weighted Median	0.042	1.15	1.00	1.32		
			Inverse variance weighted	0.143	1.14	0.96	1.35		
			Maximum likelihood	0.000	1.15	1.07	1.23		
	Uveitis	28	MR Egger	0.384	0.88	0.66	1.17	108.73	0.26
			Weighted Median	0.003	0.81	0.70	0.93		

		Inverse variance weighted	0.894	1.01	0.86	1.18		
		Maximum likelihood	0.784	1.01	0.93	1.10		
BP	44	MR Egger	0.761	0.95	0.67	1.34	46.48	0.24
		Weighted Median	0.564	1.06	0.86	1.31		
		Inverse variance weighted	0.058	1.15	1.00	1.33		
		Maximum likelihood	0.050	1.15	1.00	1.32		
HT	28	MR Egger	0.500	0.94	0.79	1.12	97.17	0.73
		Weighted Median	0.048	0.93	0.86	1.00		
		Inverse variance weighted	0.457	0.97	0.88	1.06		
		Maximum likelihood	0.163	0.97	0.92	1.01		
AS	11	MR Egger	0.570	1.06	0.86	1.31	29.45	0.81
		Weighted Median	0.040	1.04	1.00	1.09		
		Inverse variance weighted	0.080	1.04	1.00	1.08		
		Maximum likelihood	0.002	1.04	1.01	1.07		
RA	22	MR Egger	0.606	1.11	0.76	1.61	98.08	0.49
		Weighted Median	0.295	0.95	0.87	1.04		
		Inverse variance weighted	0.657	0.97	0.86	1.10		
		Maximum likelihood	0.354	0.97	0.97	1.03		
Vitiligo	13	MR Egger	0.573	0.88	0.56	1.37	5.13	0.49
		Weighted Median	0.850	0.98	0.82	1.17		

			Inverse variance weighted	0.747	1.02	0.89	1.18		
			Maximum likelihood	0.745	1.02	0.89	1.18		

Abbreviations: PsO, psoriasis; SLE, Systemic lupus erythematosus; MS, multiple sclerosis; RA, Rheumatoid arthritis; CD, Crohn's disease; UC, Ulcerative colitis; BP, Bullous pemphigoid; AS, Ankylosing spondylitis; HT, Hashimoto thyroiditis;

Table S3. The causal effects of AIDs on PsO with heterogeneity and pleiotropy test results.

Exposures	Outcomes	No.SNPs	MR methods	P	OR	95% LCI	95%UCI	Heterogeneiyt(I^2)	Egger-intercept P-value
SLE	PsO	32	MR Egger	0.822	0.99	0.95	1.04	28.09	0.30
			Weighted Median	0.288	1.02	0.99	1.05		
			Inverse variance weighted	0.104	1.02	1.00	1.04		
			Maximum likelihood	0.104	1.02	1.00	1.04		
MS		74	MR Egger	0.169	0.95	0.88	1.02	203.15	0.11
			Weighted Median	0.175	0.97	0.92	1.01		
			Inverse variance weighted	0.893	1.00	0.96	1.04		
			Maximum likelihood	0.815	1.00	0.97	1.02		
CD		56	MR Egger	0.720	1.02	0.91	1.15	169.50	0.13
			Weighted Median	0.018	1.05	1.01	1.10		
			Inverse variance weighted	0.000	1.11	1.06	1.17		
			Maximum likelihood	0.000	1.12	1.08	1.08		
UC		45	MR Egger	0.430	0.89	0.68	1.18	153.08	0.26

		Weighted Median	0.118	1.05	0.99	1.12		
		Inverse variance weighted	0.200	1.04	0.98	1.11		
		Maximum likelihood	1.046	0.96	0.94	1.08		
		MR Egger	0.742	1.01	0.93	1.11		
Uveitis	19	Weighted Median	0.534	1.01	0.97	1.06	60.32	0.78
		Inverse variance weighted	0.856	1.00	0.95	1.06		
		Maximum likelihood	0.732	1.01	0.98	1.04		
		MR Egger	0.565	0.96	0.83	1.10		
BP	5	Weighted Median	0.000	0.90	0.85	0.94	6.14	0.54
		Inverse variance weighted	0.000	0.91	0.87	0.96		
		Maximum likelihood	0.000	0.92	0.89	0.96		
		MR Egger	0.190	0.83	0.66	1.06		
HT	8	Weighted Median	0.612	1.03	0.93	1.13	19.99	0.17
		Inverse variance weighted	0.884	0.99	0.89	1.11		
		Maximum likelihood	0.806	0.99	0.93	0.93		
		MR Egger	0.191	1.99	0.74	5.37		
AS	19	Weighted Median	0.001	1.70	1.25	2.30	79.03	0.49
		Inverse variance weighted	0.084	1.44	0.95	2.17		
		Maximum likelihood	0.000	1.45	1.45	1.78		
RA	48	MR Egger	0.639	1.03	0.91	1.17	182.71	0.83

		Weighted Median	0.155	1.05	0.98	1.11		
		Inverse variance weighted	0.200	1.04	0.98	1.11		
		Maximum likelihood	0.004	0.97	0.95	0.99		
		MR Egger	0.455	1.13	0.83	1.53		
		Weighted Median	0.108	1.09	0.98	1.22		
Vitiligo	33	Inverse variance weighted	0.002	1.16	1.05	1.28	77.10	0.82
		Maximum likelihood	0.000	1.19	1.11	1.28		

Abbreviations: PsO, psoriasis; SLE, Systemic lupus erythematosus; MS, multiple sclerosis; RA, Rheumatoid arthritis; CD, Crohn's disease; UC, Ulcerative colitis; BP, Bullous pemphigoid; AS, Ankylosing spondylitis; HT, Hashimoto thyroiditis.

Table S4. The causal effects of AIDs on PsA with heterogeneity and pleiotropy test results.

Exposures	Outcomes	No.SNPs	MR methods	P	OR	95% LCI	95%UCI	Heterogeneiyt(I^2)	Egger-intercept P-value
CD	PsA	54	MR Egger	0.71	0.94	0.83	1.08	39.01	0.06
			Weighted Median	0.60	1.02	0.95	1.10		
			Inverse variance weighted	0.01	1.07	1.02	1.12		
			Maximum likelihood	0.01	1.07	1.02	1.12		
UC		49	MR Egger	0.23	1.24	0.88	1.74	138.60	0.41
			Weighted Median	0.21	1.06	0.97	1.16		
			Inverse variance weighted	0.14	1.08	0.98	1.18		
			Maximum likelihood	0.01	1.08	1.02	1.14		
Uveitis		19	MR Egger	0.47	1.05	0.92	1.19	45.87	0.65
			Weighted Median	0.51	1.03	0.95	1.12		
			Inverse variance weighted	0.08	1.07	0.99	1.16		
			Maximum likelihood	0.01	1.08	1.02	1.02		
HT		7	MR Egger	0.55	1.12	0.80	1.56	6.20	0.55
			Weighted Median	0.00	1.31	1.10	1.56		
			Inverse variance weighted	0.00	1.23	1.08	1.40		
			Maximum likelihood	0.00	1.24	1.09	1.41		
RA		32	MR Egger	0.29	1.23	0.85	1.77	24.06	0.59
			Weighted Median	0.04	1.13	1.00	1.28		

		Inverse variance weighted	0.02	1.11	1.02	1.21		
		Maximum likelihood	0.02	1.11	1.02	1.21		
AS	13	MR Egger	0.04	2.83	1.15	6.96	10.72	0.54
		Weighted Median	0.00	2.62	1.48	4.64		
		Inverse variance weighted	0.00	2.18	1.46	3.27		
		Maximum likelihood	0.00	2.24	1.49	3.38		
SLE	31	MR Egger	0.99	1.00	0.93	1.08	20.80	0.24
		Weighted Median	0.06	1.05	1.00	1.10		
		Inverse variance weighted	0.01	1.04	1.01	1.08		
		Maximum likelihood	0.01	1.04	1.01	1.08		
MS	78	MR Egger	0.90	0.99	0.88	1.12	181.34	0.69
		Weighted Median	0.02	0.92	0.85	0.99		
		Inverse variance weighted	0.70	1.01	0.95	1.08		
		Maximum likelihood	0.55	1.01	0.97	1.06		
BP	5	MR Egger	0.34	1.38	0.79	2.40	32.56	0.25
		Weighted Median	0.94	1.00	0.87	1.14		
		Inverse variance weighted	0.72	0.96	0.74	1.23		
		Maximum likelihood	0.25	0.94	0.85	1.04		
Vitiligo	33	MR Egger	0.04	1.46	1.03	2.06	34.77	0.41
		Weighted Median	0.00	1.31	1.11	1.55		

Inverse variance weighted	0.00	1.27	1.14	1.42
Maximum likelihood	0.00	1.29	1.15	1.45

Abbreviations: PsA, psoriatic arthritis; SLE, Systemic lupus erythematosus; MS, multiple sclerosis; RA, Rheumatoid arthritis; CD, Crohn's disease; UC, Ulcerative colitis; BP, Bullous pemphigoid; AS, Ankylosing spondylitis; HT, Hashimoto thyroiditis.

Table S5. The causal effects of PsA on AIDs with heterogeneity and pleiotropy test results.

Exposure	Outcome	No.SNPs	MR methods	P	OR	95% LCI	95%UCI	Heterogeneiyt(I^2)	Egger-intercept P-value
PsA	CD	9	MR Egger	0.806	0.89	0.37	2.16	52.31	0.77
			Weighted Median	0.397	1.05	0.94	1.16		
			Inverse variance weighted	0.803	1.02	0.87	1.19		
			Maximum likelihood	0.495	1.02	1.02	1.09		
	UC	9	MR Egger	0.420	1.07	0.92	1.25	21.50	0.23
			Weighted Median	0.864	0.99	0.94	1.05		
			Inverse variance weighted	0.534	0.98	0.90	1.05		
			Maximum likelihood	0.305	0.98	0.93	1.02		
	Uveitis	11	MR Egger	0.399	0.75	0.39	1.42	70.08	0.47
			Weighted Median	0.182	0.90	0.77	1.05		
			Inverse variance weighted	0.622	0.94	0.75	1.19		
			Maximum likelihood	0.191	0.94	0.86	1.03		
	HT	13	MR Egger	0.062	0.88	0.79	0.99	26.93	0.15
			Weighted Median	0.077	0.95	0.89	1.01		
			Inverse variance weighted	0.148	0.96	0.91	1.02		
			Maximum likelihood	0.032	0.96	0.92	1.00		
	RA	9	MR Egger	0.667	1.18	0.57	2.46	68.25	0.58
			Weighted Median	0.333	0.95	0.85	1.06		

		Inverse variance weighted	0.492	0.96	0.84	1.09		
		Maximum likelihood	0.031	0.95	0.91	1.00		
		MR Egger	0.895	0.96	0.57	1.64		
		Weighted Median	0.913	1.00	0.97	1.03		
AS	6	Inverse variance weighted	0.943	1.00	0.95	1.06	42.22	0.89
		Maximum likelihood	0.822	1.00	0.98	1.02		
		MR Egger	0.605	0.68	0.18	2.64		
		Weighted Median	0.249	0.92	0.79	1.06		
MS	7	Inverse variance weighted	0.889	1.01	0.83	1.23	35.71	0.59
		Maximum likelihood	0.714	1.02	0.93	1.11		
		MR Egger	0.093	0.68	0.46	1.00		
		Weighted Median	0.000	0.77	0.69	0.87		
SLE	9	Inverse variance weighted	0.295	0.89	0.72	1.10	56.02	0.16
		Maximum likelihood	0.004	0.89	0.82	0.96		
		MR Egger	0.721	1.06	0.78	1.42		
		Weighted Median	0.663	1.04	0.86	1.26		
BP	15	Inverse variance weighted	0.742	1.02	0.89	1.18	15.09	0.81
		Maximum likelihood	0.730	1.02	0.90	1.17		
		MR Egger	0.554	1.25	0.64	2.44		
		Weighted Median	0.889	1.01	0.85	1.20		

Vitiligo			Inverse variance weighted	0.597	1.04	0.90	1.20	1.23	0.61
		5	Maximum likelihood	0.596	1.04	0.90	1.20		

Abbreviations: PsA, psoriatic arthritis; SLE, Systemic lupus erythematosus; MS, multiple sclerosis; RA, Rheumatoid arthritis; CD, Crohn's disease; UC, Ulcerative colitis; BP, Bullous pemphigoid; AS, Ankylosing spondylitis; HT, Hashimoto thyroiditis.

Table S6. The causal effects of CD and vitiligo on on PsO with heterogeneity and pleiotropy test resultss after reduce heterogeneiy

Exposures	Outcomes	No.SNPs	MR methods	*P*	OR	95% LCI	95%UCI	Heterogeneiy(I^2)	Egger-intercept *P*-value
CD	PsO	46	MR Egger	0.4	1.06	0.93	1.22	115.49	0.42
			Weighted Median	1.97E-03	1.11	1.05	1.17		
			Inverse variance weighted	1.56E-05	1.12	1.06	1.18		
			Maximum likelihood	5.40E-12	1.12	1.09	1.16		
Vitiligo		26	MR Egger	0.52	1.08	0.85	1.39	26.01	0.75
			Weighted Median	0.11	1.09	0.98	1.22		
			Inverse variance weighted	9.21E-04	1.13	1.05	1.21		
			Maximum likelihood	6.20E-04	1.14	1.06	1.23		

Abbreviations: PsO, psoriasis; CD, Crohn's disease.

Table S7. Detailed information of instrumental variables utilized in the Mendelian randomization analysis of PsO on AIDs.

						Exposure				Outcome		
Exposure	Outcome	SNP	effect allele	other allele	Effect allele frequency	Beta	Se	P-value	F-statistics	Beta	SE	P-value
PsO	SLE	rs10193310	A	G	0.25	0.13	0.02	9.34E-14	2251.31	0.04	0.04	0.27
PsO	SLE	rs1250566	A	G	0.41	-0.09	0.02	6.64E-09	1430.42	0.01	0.02	0.65
PsO	SLE	rs13153019	C	T	0.27	0.10	0.02	6.67E-10	1543.77	-0.02	0.03	0.49
PsO	SLE	rs139298380	A	G	0.03	0.31	0.04	4.59E-14	1982.49	0.03	0.08	0.73
PsO	SLE	rs1611704	T	C	0.34	0.22	0.02	3.43E-49	8737.96	-0.08	0.03	0.01
PsO	SLE	rs16903065	A	C	0.12	-0.14	0.02	5.01E-09	1529.04	-0.13	0.04	0.00
PsO	SLE	rs2021511	T	C	0.27	-0.11	0.02	2.51E-10	1735.35	-0.03	0.03	0.32
PsO	SLE	rs2769979	C	T	0.60	-0.10	0.02	2.64E-10	1641.91	0.08	0.03	0.01
PsO	SLE	rs28998802	A	G	0.18	0.16	0.02	7.83E-18	2919.22	0.07	0.04	0.11
PsO	SLE	rs33980500	T	C	0.07	0.24	0.03	8.26E-19	2856.91	0.04	0.05	0.45
PsO	SLE	rs34955377	A	G	0.12	0.20	0.02	6.70E-20	3133.28	-0.21	0.06	0.00
PsO	SLE	rs60600003	G	T	0.10	0.15	0.02	2.62E-10	1537.75	0.06	0.04	0.19
PsO	SLE	rs653169	G	A	0.57	-0.09	0.02	1.14E-08	1364.28	0.11	0.03	0.00
PsO	SLE	rs674451	C	T	0.34	0.13	0.02	1.29E-16	2789.49	-0.11	0.03	0.00
PsO	SLE	rs74817271	A	G	0.07	0.22	0.03	4.12E-17	2618.00	0.04	0.06	0.52
PsO	SLE	rs7542079	C	T	0.56	0.10	0.02	2.87E-10	1669.65	0.05	0.03	0.06
PsO	SLE	rs76741620	G	A	0.06	0.17	0.03	1.06E-08	1242.41	-0.01	0.14	0.94

PsO	SLE	rs80174646	T	G	0.05	-0.23	0.04	2.81E-09	1688.09	-0.01	0.04	0.82
PsO	SLE	rs847	C	T	0.63	0.13	0.02	8.63E-16	2795.46	0.03	0.04	0.44
PsO	SLE	rs8904	A	G	0.39	-0.13	0.02	2.83E-16	2842.88	0.02	0.02	0.43
PsO	SLE	rs9346778	T	C	0.16	-0.12	0.02	1.55E-08	1400.57	0.06	0.03	0.09
PsO	MS	rs10193310	A	G	0.25	0.13	0.02	9.34E-14	2251.31	0.00	0.02	0.98
PsO	MS	rs13153019	C	T	0.27	0.10	0.02	6.67E-10	1543.77	0.08	0.02	0.00
PsO	MS	rs16903065	A	C	0.12	-0.14	0.02	5.01E-09	1529.04	0.03	0.02	0.24
PsO	MS	rs2021511	T	C	0.27	-0.11	0.02	2.51E-10	1735.35	-0.09	0.02	0.00
PsO	MS	rs2769979	C	T	0.60	-0.10	0.02	2.64E-10	1641.91	-0.04	0.02	0.02
PsO	MS	rs28998802	A	G	0.18	0.16	0.02	7.83E-18	2919.22	-0.02	0.02	0.46
PsO	MS	rs33980500	T	C	0.07	0.24	0.03	8.26E-19	2856.91	0.01	0.03	0.75
PsO	MS	rs34955377	A	G	0.12	0.20	0.02	6.70E-20	3133.28	-0.06	0.04	0.07
PsO	MS	rs60600003	G	T	0.10	0.15	0.02	2.62E-10	1537.75	0.13	0.03	0.00
PsO	MS	rs653169	G	A	0.57	-0.09	0.02	1.14E-08	1364.28	0.01	0.02	0.55
PsO	MS	rs6556423	T	C	0.64	-0.18	0.02	3.05E-32	5671.92	-0.07	0.02	0.00
PsO	MS	rs674451	C	T	0.34	0.13	0.02	1.29E-16	2789.49	-0.03	0.02	0.05
PsO	MS	rs74817271	A	G	0.07	0.22	0.03	4.12E-17	2618.00	-0.02	0.04	0.52
PsO	MS	rs7542079	C	T	0.56	0.10	0.02	2.87E-10	1669.65	-0.07	0.02	0.00
PsO	MS	rs76741620	G	A	0.06	0.17	0.03	1.06E-08	1242.41	0.15	0.05	0.00
PsO	MS	rs80174646	T	G	0.05	-0.23	0.04	2.81E-09	1688.09	0.00	0.03	0.94

PsO	MS	rs847	C	T	0.63	0.13	0.02	8.63E-16	2795.46	0.00	0.02	0.87
PsO	MS	rs8904	A	G	0.39	-0.13	0.02	2.83E-16	2842.88	0.03	0.02	0.05
PsO	MS	rs9346778	T	C	0.16	-0.12	0.02	1.55E-08	1400.57	0.07	0.02	0.00
PsO	RA	rs10193310	A	G	0.25	0.13	0.02	9.34E-14	2251.31	-0.04	0.02	0.04
PsO	RA	rs1250566	A	G	0.41	-0.09	0.02	6.64E-09	1430.42	0.04	0.01	0.01
PsO	RA	rs13153019	C	T	0.27	0.10	0.02	6.67E-10	1543.77	0.04	0.02	0.00
PsO	RA	rs139298380	A	G	0.03	0.31	0.04	4.59E-14	1982.49	0.01	0.04	0.73
PsO	RA	rs144651842	A	G	0.08	0.17	0.03	1.58E-10	1585.05	0.11	0.06	0.10
PsO	RA	rs16903065	A	C	0.12	-0.14	0.02	5.01E-09	1529.04	-0.12	0.03	0.00
PsO	RA	rs2021511	T	C	0.27	-0.11	0.02	2.51E-10	1735.35	-0.03	0.02	0.04
PsO	RA	rs2769979	C	T	0.60	-0.10	0.02	2.64E-10	1641.91	0.02	0.01	0.21
PsO	RA	rs28998802	A	G	0.18	0.16	0.02	7.83E-18	2919.22	0.00	0.02	0.92
PsO	RA	rs33980500	T	C	0.07	0.24	0.03	8.26E-19	2856.91	-0.02	0.03	0.44
PsO	RA	rs60600003	G	T	0.10	0.15	0.02	2.62E-10	1537.75	0.09	0.02	0.00
PsO	RA	rs653169	G	A	0.57	-0.09	0.02	1.14E-08	1364.28	0.04	0.02	0.01
PsO	RA	rs6556423	T	C	0.64	-0.18	0.02	3.05E-32	5671.92	0.01	0.01	0.42
PsO	RA	rs674451	C	T	0.34	0.13	0.02	1.29E-16	2789.49	-0.07	0.01	0.00
PsO	RA	rs74817271	A	G	0.07	0.22	0.03	4.12E-17	2618.00	-0.01	0.03	0.74
PsO	RA	rs7542079	C	T	0.56	0.10	0.02	2.87E-10	1669.65	0.01	0.01	0.63
PsO	RA	rs76741620	G	A	0.06	0.17	0.03	1.06E-08	1242.41	0.02	0.04	0.72

PsO	RA	rs78456138	T	C	0.02	-0.36	0.06	6.61E-10	1992.49	-0.05	0.07	0.40
PsO	RA	rs80174646	T	G	0.05	-0.23	0.04	2.81E-09	1688.09	0.01	0.03	0.77
PsO	RA	rs847	C	T	0.63	0.13	0.02	8.63E-16	2795.46	-0.02	0.02	0.31
PsO	RA	rs8904	A	G	0.39	-0.13	0.02	2.83E-16	2842.88	-0.02	0.02	0.28
PsO	RA	rs9346778	T	C	0.16	-0.12	0.02	1.55E-08	1400.57	0.02	0.01	0.30
PsO	Uveitis	rs10193310	A	G	0.25	0.13	0.02	9.34E-14	2251.31	0.03	0.03	0.36
PsO	Uveitis	rs12206050	T	A	0.32	0.10	0.02	1.50E-10	1676.98	0.12	0.03	0.00
PsO	Uveitis	rs1250566	A	G	0.41	-0.09	0.02	6.64E-09	1430.42	0.04	0.03	0.17
PsO	Uveitis	rs13153019	C	T	0.27	0.10	0.02	6.67E-10	1543.77	-0.02	0.03	0.49
PsO	Uveitis	rs139298380	A	G	0.03	0.31	0.04	4.59E-14	1982.49	-0.07	0.08	0.43
PsO	Uveitis	rs144651842	A	G	0.08	0.17	0.03	1.58E-10	1585.05	0.09	0.06	0.12
PsO	Uveitis	rs1611704	T	C	0.34	0.22	0.02	3.43E-49	8737.96	0.12	0.03	0.00
PsO	Uveitis	rs16903065	A	C	0.12	-0.14	0.02	5.01E-09	1529.04	-0.02	0.04	0.57
PsO	Uveitis	rs181316459	C	G	0.05	0.33	0.03	3.99E-24	3717.38	0.37	0.07	0.00
PsO	Uveitis	rs2021511	T	C	0.27	-0.11	0.02	2.51E-10	1735.35	-0.05	0.03	0.11
PsO	Uveitis	rs2769979	C	T	0.60	-0.10	0.02	2.64E-10	1641.91	0.01	0.03	0.73
PsO	Uveitis	rs28732090	G	C	0.06	0.80	0.03	1.00E-200	28114.64	-0.24	0.06	0.00
PsO	Uveitis	rs28998802	A	G	0.18	0.16	0.02	7.83E-18	2919.22	-0.04	0.04	0.35
PsO	Uveitis	rs33980500	T	C	0.07	0.24	0.03	8.26E-19	2856.91	0.14	0.05	0.01
PsO	Uveitis	rs34536443	C	G	0.03	-0.28	0.05	2.73E-09	1750.56	-0.09	0.08	0.27

PsO	Uveitis	rs4400255	A	T	0.10	0.18	0.02	2.14E-14	2211.40	-0.07	0.05	0.16
PsO	Uveitis	rs60600003	G	T	0.10	0.15	0.02	2.62E-10	1537.75	-0.06	0.05	0.21
PsO	Uveitis	rs653169	G	A	0.57	-0.09	0.02	1.14E-08	1364.28	0.02	0.03	0.47
PsO	Uveitis	rs6556423	T	C	0.64	-0.18	0.02	3.05E-32	5671.92	0.03	0.03	0.30
PsO	Uveitis	rs674451	C	T	0.34	0.13	0.02	1.29E-16	2789.49	0.03	0.03	0.39
PsO	Uveitis	rs74817271	A	G	0.07	0.22	0.03	4.12E-17	2618.00	0.03	0.05	0.53
PsO	Uveitis	rs7542079	C	T	0.56	0.10	0.02	2.87E-10	1669.65	0.02	0.03	0.48
PsO	Uveitis	rs76741620	G	A	0.06	0.17	0.03	1.06E-08	1242.41	-0.09	0.06	0.16
PsO	Uveitis	rs78456138	T	C	0.02	-0.36	0.06	6.61E-10	1992.49	0.03	0.10	0.74
PsO	Uveitis	rs80174646	T	G	0.05	-0.23	0.04	2.81E-09	1688.09	-0.11	0.06	0.08
PsO	Uveitis	rs847	C	T	0.63	0.13	0.02	8.63E-16	2795.46	-0.01	0.03	0.68
PsO	Uveitis	rs8904	A	G	0.39	-0.13	0.02	2.83E-16	2842.88	0.03	0.03	0.31
PsO	Uveitis	rs9346778	T	C	0.16	-0.12	0.02	1.55E-08	1400.57	0.01	0.04	0.83
PsO	CD	rs13153019	C	T	0.27	0.10	0.02	6.67E-10	1543.77	0.04	0.02	0.03
PsO	CD	rs139298380	A	G	0.03	0.31	0.04	4.59E-14	1982.49	0.06	0.04	0.15
PsO	CD	rs1611704	T	C	0.34	0.22	0.02	3.43E-49	8737.96	-0.02	0.02	0.29
PsO	CD	rs2769979	C	T	0.60	-0.10	0.02	2.64E-10	1641.91	0.00	0.02	0.85
PsO	CD	rs28998802	A	G	0.18	0.16	0.02	7.83E-18	2919.22	0.01	0.02	0.73
PsO	CD	rs33980500	T	C	0.07	0.24	0.03	8.26E-19	2856.91	0.09	0.03	0.00
PsO	CD	rs34955377	A	G	0.12	0.20	0.02	6.70E-20	3133.28	-0.02	0.03	0.59

PsO	CD	rs60600003	G	T	0.10	0.15	0.02	2.62E-10	1537.75	0.02	0.03	0.45
PsO	CD	rs653169	G	A	0.57	-0.09	0.02	1.14E-08	1364.28	0.02	0.02	0.23
PsO	CD	rs674451	C	T	0.34	0.13	0.02	1.29E-16	2789.49	0.03	0.02	0.05
PsO	CD	rs74817271	A	G	0.07	0.22	0.03	4.12E-17	2618.00	0.04	0.03	0.23
PsO	CD	rs7542079	C	T	0.56	0.10	0.02	2.87E-10	1669.65	-0.07	0.02	0.00
PsO	CD	rs76741620	G	A	0.06	0.17	0.03	1.06E-08	1242.41	0.15	0.05	0.00
PsO	CD	rs78456138	T	C	0.02	-0.36	0.06	6.61E-10	1992.49	0.03	0.06	0.59
PsO	CD	rs847	C	T	0.63	0.13	0.02	8.63E-16	2795.46	0.07	0.02	0.00
PsO	CD	rs8904	A	G	0.39	-0.13	0.02	2.83E-16	2842.88	0.06	0.02	0.00
PsO	CD	rs9346778	T	C	0.16	-0.12	0.02	1.55E-08	1400.57	-0.05	0.02	0.01
PsO	UC	rs10193310	A	G	0.25	0.13	0.02	9.34E-14	2251.31	0.06	0.02	0.00
PsO	UC	rs1250566	A	G	0.41	-0.09	0.02	6.64E-09	1430.42	-0.04	0.02	0.01
PsO	UC	rs13153019	C	T	0.27	0.10	0.02	6.67E-10	1543.77	0.08	0.02	0.00
PsO	UC	rs139298380	A	G	0.03	0.31	0.04	4.59E-14	1982.49	0.02	0.04	0.64
PsO	UC	rs1611704	T	C	0.34	0.22	0.02	3.43E-49	8737.96	0.08	0.02	0.00
PsO	UC	rs16903065	A	C	0.12	-0.14	0.02	5.01E-09	1529.04	-0.03	0.02	0.27
PsO	UC	rs2021511	T	C	0.27	-0.11	0.02	2.51E-10	1735.35	-0.06	0.02	0.00
PsO	UC	rs2769979	C	T	0.60	-0.10	0.02	2.64E-10	1641.91	0.03	0.02	0.03
PsO	UC	rs28998802	A	G	0.18	0.16	0.02	7.83E-18	2919.22	0.00	0.02	0.84
PsO	UC	rs34955377	A	G	0.12	0.20	0.02	6.70E-20	3133.28	0.13	0.03	0.00

PsO	UC	rs60600003	G	T	0.10	0.15	0.02	2.62E-10	1537.75	0.09	0.03	0.00
PsO	UC	rs653169	G	A	0.57	-0.09	0.02	1.14E-08	1364.28	-0.03	0.02	0.10
PsO	UC	rs674451	C	T	0.34	0.13	0.02	1.29E-16	2789.49	0.00	0.02	0.80
PsO	UC	rs74817271	A	G	0.07	0.22	0.03	4.12E-17	2618.00	-0.07	0.04	0.04
PsO	UC	rs7542079	C	T	0.56	0.10	0.02	2.87E-10	1669.65	-0.02	0.02	0.13
PsO	UC	rs76741620	G	A	0.06	0.17	0.03	1.06E-08	1242.41	0.09	0.05	0.06
PsO	UC	rs78456138	T	C	0.02	-0.36	0.06	6.61E-10	1992.49	0.19	0.06	0.00
PsO	UC	rs847	C	T	0.63	0.13	0.02	8.63E-16	2795.46	0.02	0.02	0.39
PsO	UC	rs8904	A	G	0.39	-0.13	0.02	2.83E-16	2842.88	0.07	0.02	0.00
PsO	UC	rs9346778	T	C	0.16	-0.12	0.02	1.55E-08	1400.57	0.03	0.02	0.13
PsO	BP	rs1108618	G	A	NA	-0.11	0.02	1.62E-11	45.39	-0.02	0.06	0.77
PsO	BP	rs11205044	C	T	NA	0.19	0.02	6.30E-31	133.95	0.12	0.07	0.07
PsO	BP	rs11213262	A	C	NA	-0.13	0.02	4.07E-16	66.05	-0.01	0.07	0.83
PsO	BP	rs11249215	A	G	NA	0.15	0.02	1.17E-21	91.75	-0.07	0.06	0.29
PsO	BP	rs113935720	C	T	NA	-0.37	0.04	4.22E-25	107.18	0.09	0.15	0.54
PsO	BP	rs115059666	A	G	NA	0.46	0.07	3.23E-12	48.59	-0.34	0.38	0.37
PsO	BP	rs11767350	G	A	NA	-0.09	0.02	3.00E-09	35.16	-0.02	0.07	0.71
PsO	BP	rs11795343	C	T	NA	-0.11	0.02	2.36E-12	49.27	-0.05	0.07	0.48
PsO	BP	rs12046909	T	C	NA	-0.13	0.02	1.68E-09	36.44	0.05	0.08	0.51
PsO	BP	rs12133684	A	G	NA	0.11	0.02	4.47E-09	34.34	0.07	0.08	0.37

PsO	BP	rs1295685	G	A	NA	0.17	0.02	1.33E-18	77.82	-0.03	0.07	0.68
PsO	BP	rs1611236	A	G	NA	0.15	0.02	8.51E-21	86.95	0.06	0.08	0.44
PsO	BP	rs1810563	A	G	NA	0.10	0.02	1.22E-09	37.08	0.05	0.07	0.45
PsO	BP	rs2021511	T	C	NA	-0.11	0.02	1.05E-10	41.71	-0.02	0.07	0.75
PsO	BP	rs2056991	C	T	NA	-0.15	0.02	3.43E-22	94.24	-0.08	0.06	0.19
PsO	BP	rs2066819	T	C	NA	-0.32	0.03	6.05E-21	88.25	0.02	0.14	0.86
PsO	BP	rs2111485	G	A	NA	0.15	0.02	1.14E-20	87.23	0.04	0.06	0.57
PsO	BP	rs2451258	T	C	NA	-0.09	0.02	6.36E-09	33.80	-0.17	0.07	0.01
PsO	BP	rs2633310	T	G	NA	-0.11	0.02	1.64E-12	49.88	0.02	0.06	0.81
PsO	BP	rs2735009	A	G	NA	-0.31	0.02	3.43E-59	264.01	0.02	0.08	0.83
PsO	BP	rs28998802	A	G	NA	0.20	0.02	3.54E-22	93.38	0.01	0.08	0.92
PsO	BP	rs33980500	T	C	NA	0.39	0.03	1.04E-48	214.70	0.02	0.12	0.87
PsO	BP	rs3802826	G	A	NA	-0.11	0.02	9.07E-13	51.14	-0.08	0.06	0.23
PsO	BP	rs39841	A	G	NA	-0.16	0.02	3.66E-22	93.95	0.08	0.07	0.25
PsO	BP	rs4685408	A	G	NA	-0.11	0.02	3.31E-13	52.75	0.07	0.06	0.27
PsO	BP	rs4712520	C	T	NA	0.12	0.02	8.15E-10	37.65	-0.02	0.08	0.82
PsO	BP	rs4889526	A	C	NA	0.12	0.02	1.57E-14	59.30	-0.03	0.06	0.62
PsO	BP	rs4919446	A	G	NA	-0.10	0.02	4.11E-08	30.22	-0.04	0.06	0.53
PsO	BP	rs4921485	T	C	NA	-0.31	0.02	3.38E-78	351.72	-0.16	0.07	0.02
PsO	BP	rs510372	T	C	NA	-0.10	0.02	1.34E-10	41.35	-0.13	0.06	0.04

PsO	BP	rs56203253	A	C	NA	0.37	0.06	2.79E-10	39.81	-0.76	0.30	0.01
PsO	BP	rs582757	T	C	NA	-0.18	0.02	8.11E-28	119.56	-0.02	0.07	0.76
PsO	BP	rs59960858	A	C	NA	-0.22	0.02	5.43E-21	88.05	-0.04	0.08	0.60
PsO	BP	rs653178	T	C	NA	-0.09	0.02	1.76E-08	31.61	0.05	0.06	0.45
PsO	BP	rs6894840	G	T	NA	0.11	0.02	2.45E-11	44.36	-0.08	0.06	0.22
PsO	BP	rs7141014	C	T	NA	-0.11	0.02	3.18E-08	30.47	0.00	0.09	0.98
PsO	BP	rs74899623	A	G	NA	-0.25	0.04	7.24E-09	33.54	-0.30	0.20	0.13
PsO	BP	rs771576	C	T	NA	0.10	0.02	1.15E-09	37.14	-0.04	0.07	0.56
PsO	BP	rs9258357	C	T	NA	0.27	0.02	3.47E-33	143.78	0.01	0.07	0.84
PsO	BP	rs9468618	T	C	NA	-0.16	0.03	1.43E-09	36.72	-0.11	0.14	0.43
PsO	BP	rs9504361	G	A	NA	-0.10	0.02	4.79E-10	38.68	-0.08	0.06	0.22
PsO	BP	rs9513593	A	G	NA	-0.12	0.02	9.52E-10	37.34	-0.01	0.08	0.90
PsO	BP	rs9695923	T	C	NA	-0.09	0.02	2.27E-09	35.62	-0.12	0.06	0.06
PsO	BP	rs9749064	G	A	NA	-0.09	0.02	2.37E-08	31.29	-0.07	0.06	0.27
PsO	HT	rs10193310	A	G	0.25	0.13	0.02	9.34E-14	2251.31	0.01	0.02	0.52
PsO	HT	rs12206050	T	A	0.32	0.10	0.02	1.50E-10	1676.98	-0.02	0.02	0.25
PsO	HT	rs1250566	A	G	0.41	-0.09	0.02	6.64E-09	1430.42	0.00	0.02	0.78
PsO	HT	rs13153019	C	T	0.27	0.10	0.02	6.67E-10	1543.77	0.02	0.02	0.30
PsO	HT	rs139298380	A	G	0.03	0.31	0.04	4.59E-14	1982.49	-0.04	0.05	0.42
PsO	HT	rs144651842	A	G	0.08	0.17	0.03	1.58E-10	1585.05	0.04	0.03	0.23

PsO	HT	rs1611704	T	C	0.34	0.22	0.02	3.43E-49	8737.96	-0.04	0.02	0.06
PsO	HT	rs16903065	A	C	0.12	-0.14	0.02	5.01E-09	1529.04	-0.02	0.03	0.37
PsO	HT	rs2021511	T	C	0.27	-0.11	0.02	2.51E-10	1735.35	-0.03	0.02	0.08
PsO	HT	rs2769979	C	T	0.60	-0.10	0.02	2.64E-10	1641.91	-0.02	0.02	0.37
PsO	HT	rs28732090	G	C	0.06	0.80	0.03	1.00E-200	28114.64	-0.07	0.04	0.04
PsO	HT	rs28998802	A	G	0.18	0.16	0.02	7.83E-18	2919.22	0.00	0.02	0.84
PsO	HT	rs33980500	T	C	0.07	0.24	0.03	8.26E-19	2856.91	0.04	0.03	0.28
PsO	HT	rs34536443	C	G	0.03	-0.28	0.05	2.73E-09	1750.56	-0.25	0.05	0.00
PsO	HT	rs34955377	A	G	0.12	0.20	0.02	6.70E-20	3133.28	-0.12	0.03	0.00
PsO	HT	rs4400255	A	T	0.10	0.18	0.02	2.14E-14	2211.40	-0.05	0.03	0.08
PsO	HT	rs60600003	G	T	0.10	0.15	0.02	2.62E-10	1537.75	0.03	0.03	0.25
PsO	HT	rs653169	G	A	0.57	-0.09	0.02	1.14E-08	1364.28	0.01	0.02	0.59
PsO	HT	rs6556423	T	C	0.64	-0.18	0.02	3.05E-32	5671.92	0.03	0.02	0.11
PsO	HT	rs674451	C	T	0.34	0.13	0.02	1.29E-16	2789.49	0.01	0.02	0.59
PsO	HT	rs74817271	A	G	0.07	0.22	0.03	4.12E-17	2618.00	0.01	0.03	0.64
PsO	HT	rs7542079	C	T	0.56	0.10	0.02	2.87E-10	1669.65	0.00	0.02	0.79
PsO	HT	rs76741620	G	A	0.06	0.17	0.03	1.06E-08	1242.41	0.00	0.04	0.98
PsO	HT	rs78456138	T	C	0.02	-0.36	0.06	6.61E-10	1992.49	-0.23	0.06	0.00
PsO	HT	rs80174646	T	G	0.05	-0.23	0.04	2.81E-09	1688.09	0.00	0.04	0.96
PsO	HT	rs847	C	T	0.63	0.13	0.02	8.63E-16	2795.46	-0.02	0.02	0.19

PsO	HT	rs8904	A	G	0.39	-0.13	0.02	2.83E-16	2842.88	-0.01	0.02	0.76
PsO	HT	rs9346778	T	C	0.16	-0.12	0.02	1.55E-08	1400.57	0.06	0.02	0.00

Abbreviations: PsO, psoriasis; SLE, Systemic lupus erythematosus; MS, multiple sclerosis; RA, Rheumatoid arthritis; CD, Crohn's disease; UC, Ulcerative colitis; BP, Bullous pemphigoid; AS, Ankylosing spondylitis; HT, Hashimoto thyroiditis; AIDs, Autoimmune diseases.

Table S8. Detailed information of instrumental variables utilized in the Mendelian randomization analysis of PsA on AIDs.

					Exposure					Outcome		
**Exposure**	**Outcome**	**SNP**	**effect allele**	**other allele**	**Effect allele frequency**	**Beta**	**Se**	**P-value**	**F-statistics**	**Beta**	**SE**	**P-value**
PsA	SLE	rs115174302	T	C	0.09	0.35	0.04	7.97E-19	5142.05	-0.08	0.07	0.24
PsA	SLE	rs115625939	G	A	0.13	-0.22	0.04	1.04E-08	2862.74	-0.12	0.04	0.01
PsA	SLE	rs2546890	G	A	0.41	-0.23	0.03	1.23E-18	6315.99	-0.11	0.03	0.00
PsA	SLE	rs28998802	A	G	0.18	0.21	0.03	1.86E-11	3273.86	0.07	0.04	0.11
PsA	SLE	rs4921493	C	T	0.32	-0.18	0.03	2.80E-11	3620.82	-0.01	0.03	0.75
PsA	SLE	rs674451	C	T	0.34	0.17	0.03	7.14E-11	3224.56	-0.11	0.03	0.00
PsA	SLE	rs847	C	T	0.64	0.19	0.03	3.53E-12	3961.55	0.03	0.04	0.44
PsA	SLE	rs8904	A	G	0.39	-0.17	0.03	8.58E-11	3388.18	0.02	0.02	0.43
PsA	SLE	rs9461693	A	G	0.23	0.61	0.03	1.22E-121	37270.46	-0.17	0.03	0.00
PsA	MS	rs11085727	T	C	0.26	-0.19	0.03	2.63E-10	3318.80	-0.07	0.02	0.00

PsA	MS	rs115174302	T	C	0.09	0.35	0.04	7.97E-19	5142.05	-0.11	0.05	0.02
PsA	MS	rs28998802	A	G	0.18	0.21	0.03	1.86E-11	3273.86	-0.02	0.02	0.46
PsA	MS	rs4921493	C	T	0.32	-0.18	0.03	2.80E-11	3620.82	-0.05	0.02	0.00
PsA	MS	rs674451	C	T	0.34	0.17	0.03	7.14E-11	3224.56	-0.03	0.02	0.05
PsA	MS	rs847	C	T	0.64	0.19	0.03	3.53E-12	3961.55	0.00	0.02	0.87
PsA	MS	rs8904	A	G	0.39	-0.17	0.03	8.58E-11	3388.18	0.03	0.02	0.05
PsA	RA	rs115174302	T	C	0.09	0.35	0.04	7.97E-19	5142.05	0.10	0.03	0.00
PsA	RA	rs144651842	A	G	0.08	0.27	0.04	1.83E-09	2490.50	0.11	0.06	0.10
PsA	RA	rs2523560	T	C	0.51	-0.26	0.02	6.60E-25	8372.71	0.05	0.01	0.00
PsA	RA	rs2546890	G	A	0.41	-0.23	0.03	1.23E-18	6315.99	-0.03	0.01	0.02
PsA	RA	rs28998802	A	G	0.18	0.21	0.03	1.86E-11	3273.86	0.00	0.02	0.92
PsA	RA	rs4921493	C	T	0.32	-0.18	0.03	2.80E-11	3620.82	0.01	0.02	0.49
PsA	RA	rs674451	C	T	0.34	0.17	0.03	7.14E-11	3224.56	-0.07	0.01	0.00
PsA	RA	rs847	C	T	0.64	0.19	0.03	3.53E-12	3961.55	-0.02	0.02	0.31
PsA	RA	rs8904	A	G	0.39	-0.17	0.03	8.58E-11	3388.18	-0.02	0.02	0.28
PsA	Uveitis	rs11085727	T	C	0.26	-0.19	0.03	2.63E-10	3318.80	-0.08	0.03	0.01
PsA	Uveitis	rs115625939	G	A	0.13	-0.22	0.04	1.04E-08	2862.74	-0.04	0.04	0.42
PsA	Uveitis	rs144651842	A	G	0.08	0.27	0.04	1.83E-09	2490.50	0.09	0.06	0.12
PsA	Uveitis	rs181316459	C	G	0.05	0.38	0.05	2.97E-12	3203.81	0.37	0.07	0.00
PsA	Uveitis	rs2546890	G	A	0.41	-0.23	0.03	1.23E-18	6315.99	0.01	0.03	0.64

PsA	Uveitis	rs28998802	A	G	0.18	0.21	0.03	1.86E-11	3273.86	-0.04	0.04	0.35
PsA	Uveitis	rs4921493	C	T	0.32	-0.18	0.03	2.80E-11	3620.82	0.08	0.03	0.01
PsA	Uveitis	rs674451	C	T	0.34	0.17	0.03	7.14E-11	3224.56	0.03	0.03	0.39
PsA	Uveitis	rs847	C	T	0.64	0.19	0.03	3.53E-12	3961.55	-0.01	0.03	0.68
PsA	Uveitis	rs8904	A	G	0.39	-0.17	0.03	8.58E-11	3388.18	0.03	0.03	0.31
PsA	Uveitis	rs9265889	A	T	0.13	0.60	0.03	1.03E-74	21150.19	-0.25	0.05	0.00
PsA	CD	rs11085727	T	C	0.26	-0.19	0.03	2.63E-10	3318.80	-0.06	0.02	0.00
PsA	CD	rs115174302	T	C	0.09	0.35	0.04	7.97E-19	5142.05	0.01	0.04	0.79
PsA	CD	rs115625939	G	A	0.13	-0.22	0.04	1.04E-08	2862.74	0.10	0.03	0.00
PsA	CD	rs2523560	T	C	0.51	-0.26	0.02	6.60E-25	8372.71	-0.02	0.02	0.35
PsA	CD	rs2546890	G	A	0.41	-0.23	0.03	1.23E-18	6315.99	0.02	0.02	0.18
PsA	CD	rs28998802	A	G	0.18	0.21	0.03	1.86E-11	3273.86	0.01	0.02	0.73
PsA	CD	rs674451	C	T	0.34	0.17	0.03	7.14E-11	3224.56	0.03	0.02	0.05
PsA	CD	rs847	C	T	0.64	0.19	0.03	3.53E-12	3961.55	0.07	0.02	0.00
PsA	CD	rs8904	A	G	0.39	-0.17	0.03	8.58E-11	3388.18	0.06	0.02	0.00
PsA	UC	rs11085727	T	C	0.26	-0.19	0.03	2.63E-10	3318.80	0.01	0.02	0.48
PsA	UC	rs115174302	T	C	0.09	0.35	0.04	7.97E-19	5142.05	0.07	0.04	0.09
PsA	UC	rs115625939	G	A	0.13	-0.22	0.04	1.04E-08	2862.74	0.04	0.03	0.16
PsA	UC	rs2546890	G	A	0.41	-0.23	0.03	1.23E-18	6315.99	0.01	0.02	0.56
PsA	UC	rs28998802	A	G	0.18	0.21	0.03	1.86E-11	3273.86	0.00	0.02	0.84

PsA	UC	rs674451	C	T	0.34	0.17	0.03	7.14E-11	3224.56	0.00	0.02	0.80
PsA	UC	rs847	C	T	0.64	0.19	0.03	3.53E-12	3961.55	0.02	0.02	0.39
PsA	UC	rs8904	A	G	0.39	-0.17	0.03	8.58E-11	3388.18	0.07	0.02	0.00
PsA	UC	rs9461693	A	G	0.23	0.61	0.03	1.22E-121	37270.46	0.00	0.02	0.90
PsA	Vitiligo	rs115174302	T	C	0.09	0.35	0.04	7.97E-19	5142.05	0.05	0.06	0.09
PsA	Vitiligo	rs2546890	G	A	0.41	-0.23	0.03	1.23E-18	6315.99	0.00	0.03	0.76
PsA	Vitiligo	rs4921493	C	T	0.32	-0.18	0.03	2.80E-11	3620.82	0.01	0.03	0.61
PsA	Vitiligo	rs674451	C	T	0.34	0.17	0.03	7.14E-11	3224.56	0.00	0.03	0.78
PsA	Vitiligo	rs847	C	T	0.64	0.19	0.03	3.53E-12	3961.55	0.04	0.04	0.03
PsA	BP	rs10476295	G	A	0.38	-0.17	0.02	2.64E-10	346.11	0.03	0.06	0.64
PsA	BP	rs13203895	T	C	0.14	0.85	0.07	1.75E-153	5456.02	0.04	0.11	0.70
PsA	BP	rs1395621	C	T	0.49	0.16	0.03	6.48E-11	335.14	-0.03	0.06	0.65
PsA	BP	rs1655901	C	T	0.59	0.34	0.04	6.16E-38	1548.86	-0.05	0.06	0.42
PsA	BP	rs2020854	C	T	0.07	-0.36	0.03	1.26E-13	425.87	0.03	0.14	0.85
PsA	BP	rs2111485	G	A	0.62	0.22	0.03	1.24E-20	624.54	0.04	0.06	0.57
PsA	BP	rs33980500	T	C	0.08	0.51	0.06	1.14E-36	1032.37	0.02	0.12	0.87
PsA	BP	rs34536443	C	G	0.04	-0.54	0.04	1.16E-14	620.85	0.18	0.18	0.33
PsA	BP	rs3794767	C	T	0.65	0.18	0.03	4.73E-13	387.07	0.07	0.07	0.29
PsA	BP	rs5754467	G	A	0.20	0.18	0.03	1.61E-09	264.48	0.02	0.07	0.82
PsA	BP	rs610604	T	G	0.67	-0.14	0.02	7.76E-09	233.49	0.00	0.07	0.98

PsA	BP	rs62377586	A	G	0.32	-0.31	0.02	8.17E-35	1156.04	-0.20	0.07	0.00
PsA	BP	rs73510898	A	G	0.09	-0.23	0.03	2.94E-08	234.70	0.12	0.11	0.31
PsA	BP	rs848	C	A	0.83	0.25	0.04	9.49E-16	472.61	-0.03	0.07	0.65
PsA	BP	rs9263869	C	T	0.32	-0.22	0.02	3.57E-18	556.55	0.08	0.07	0.24
PsA	AS	rs11085727	T	C	0.26	-0.19	0.03	2.63E-10	3318.80	-0.01	0.00	0.00
PsA	AS	rs2546890	G	A	0.41	-0.23	0.03	1.23E-18	6315.99	0.00	0.00	0.69
PsA	AS	rs28998802	A	G	0.18	0.21	0.03	1.86E-11	3273.86	0.01	0.01	0.23
PsA	AS	rs4921493	C	T	0.32	-0.18	0.03	2.80E-11	3620.82	0.02	0.00	0.00
PsA	AS	rs674451	C	T	0.34	0.17	0.03	7.14E-11	3224.56	0.01	0.00	0.02
PsA	AS	rs8904	A	G	0.39	-0.17	0.03	8.58E-11	3388.18	0.00	0.00	0.92
PsA	HT	rs11085727	T	C	0.26	-0.19	0.03	2.63E-10	3318.80	-0.01	0.02	0.46
PsA	HT	rs115174302	T	C	0.09	0.35	0.04	7.97E-19	5142.05	-0.05	0.03	0.09
PsA	HT	rs115625939	G	A	0.13	-0.22	0.04	1.04E-08	2862.74	-0.08	0.03	0.00
PsA	HT	rs144651842	A	G	0.08	0.27	0.04	1.83E-09	2490.50	0.04	0.03	0.23
PsA	HT	rs2523560	T	C	0.51	-0.26	0.02	6.60E-25	8372.71	0.01	0.02	0.64
PsA	HT	rs2546890	G	A	0.41	-0.23	0.03	1.23E-18	6315.99	0.01	0.02	0.51
PsA	HT	rs28998802	A	G	0.18	0.21	0.03	1.86E-11	3273.86	0.00	0.02	0.84
PsA	HT	rs4921493	C	T	0.32	-0.18	0.03	2.80E-11	3620.82	0.01	0.02	0.53
PsA	HT	rs674451	C	T	0.34	0.17	0.03	7.14E-11	3224.56	0.01	0.02	0.59
PsA	HT	rs847	C	T	0.64	0.19	0.03	3.53E-12	3961.55	-0.02	0.02	0.19

PsA	HT	rs8904	A	G	0.39	-0.17	0.03	8.58E-11	3388.18	-0.01	0.02	0.76
PsA	HT	rs9265889	A	T	0.13	0.60	0.03	1.03E-74	21150.19	0.00	0.03	0.96
PsA	HT	rs9461693	A	G	0.23	0.61	0.03	1.22E-121	37270.46	-0.07	0.02	0.00

Abbreviations: PsA, psoriatic arthritis; SLE, Systemic lupus erythematosus; MS, multiple sclerosis; RA, Rheumatoid arthritis; CD, Crohn's disease; UC, Ulcerative colitis; BP, Bullous pemphigoid; AS, Ankylosing spondylitis; HT, Hashimoto thyroiditis; AIDs, Autoimmune diseases.

Table S9. Detailed information of instrumental variables utilized in the Mendelian randomization analysis of AIDs on PsA.

						Exposure				Outcome		
**Exposure**	**Outcome**	**SNP**	**effect allele**	**other allele**	**Effect allele frequency**	**Beta**	**Se**	**P-value**	**F-statistics**	**Beta**	**SE**	**P-value**
SLE	PsA	rs10200680	T	C	NA	-0.25	0.04	4.96E-09	34.20	0.01	0.03	0.82
SLE	PsA	rs10912578	G	A	NA	-0.25	0.03	1.65E-15	63.44	0.02	0.03	0.48
SLE	PsA	rs1143679	A	G	NA	0.58	0.04	5.03E-48	211.97	-0.01	0.04	0.86
SLE	PsA	rs12094036	C	T	NA	-0.33	0.06	1.37E-08	32.23	-0.05	0.04	0.22
SLE	PsA	rs12524498	T	G	NA	-0.67	0.12	2.48E-08	31.07	-0.06	0.09	0.50
SLE	PsA	rs13019891	T	G	NA	-0.56	0.03	1.65E-83	374.80	-0.01	0.03	0.61
SLE	PsA	rs13136219	T	C	NA	-0.17	0.03	3.50E-10	39.37	-0.02	0.03	0.40
SLE	PsA	rs13332649	G	A	NA	-0.31	0.04	5.43E-17	70.16	-0.07	0.03	0.02
SLE	PsA	rs1464446	T	G	NA	-0.33	0.04	2.79E-16	66.94	-0.03	0.03	0.45
SLE	PsA	rs150180633	T	C	NA	0.93	0.07	2.66E-41	181.17	0.01	0.20	0.94

SLE	PsA	rs17849501	T	C	NA	0.81	0.05	1.81E-59	264.44	0.03	0.07	0.61
SLE	PsA	rs2431697	C	T	NA	-0.22	0.03	2.60E-14	58.01	-0.04	0.03	0.09
SLE	PsA	rs2459611	T	C	NA	0.26	0.05	7.62E-09	33.37	-0.04	0.06	0.43
SLE	PsA	rs2573219	C	A	NA	0.59	0.04	1.13E-42	187.44	-0.03	0.05	0.45
SLE	PsA	rs268124	T	C	NA	0.19	0.03	8.60E-09	33.13	0.02	0.03	0.40
SLE	PsA	rs34703115	C	T	NA	-0.62	0.10	4.08E-09	34.58	0.04	0.07	0.51
SLE	PsA	rs35000415	T	C	NA	0.59	0.04	1.86E-45	200.20	0.03	0.04	0.41
SLE	PsA	rs353608	G	A	NA	0.19	0.03	2.93E-11	44.22	-0.01	0.03	0.80
SLE	PsA	rs3747093	A	G	NA	0.26	0.03	2.88E-14	57.81	0.03	0.03	0.33
SLE	PsA	rs4274624	T	C	NA	-0.56	0.03	9.73E-66	293.21	-0.03	0.03	0.31
SLE	PsA	rs4388254	T	C	NA	0.38	0.06	3.71E-10	39.25	-0.05	0.04	0.23
SLE	PsA	rs4661543	G	T	NA	0.27	0.04	9.40E-11	41.94	0.02	0.05	0.76
SLE	PsA	rs4916215	T	C	NA	0.22	0.03	5.07E-11	43.15	0.02	0.03	0.51
SLE	PsA	rs6671847	A	G	NA	0.20	0.03	6.64E-12	47.12	0.04	0.03	0.09
SLE	PsA	rs6889239	C	T	NA	0.28	0.03	2.19E-18	76.50	0.04	0.03	0.16
SLE	PsA	rs7097397	A	G	NA	-0.19	0.03	8.60E-11	42.11	0.00	0.03	0.90
SLE	PsA	rs73050535	T	C	NA	-0.71	0.12	9.11E-09	33.02	-0.10	0.18	0.57
SLE	PsA	rs73068668	A	G	NA	-0.31	0.06	4.40E-08	29.96	-0.05	0.05	0.29
SLE	PsA	rs7768653	T	C	NA	-0.21	0.03	3.11E-12	48.61	0.01	0.03	0.72
SLE	PsA	rs7823055	T	G	NA	-0.35	0.03	1.64E-34	150.09	-0.02	0.03	0.38

SLE	PsA	rs9852014	G	A	NA	0.62	0.05	2.26E-36	158.61	0.03	0.05	0.57
MS	PsA	rs10063294	A	G	NA	-0.10	0.02	1.13E-09	37.09	-0.04	0.03	0.12
MS	PsA	rs1014486	C	T	NA	0.11	0.02	1.36E-10	41.21	-0.02	0.03	0.45
MS	PsA	rs1026916	G	A	NA	-0.13	0.02	1.02E-13	55.33	0.02	0.03	0.45
MS	PsA	rs1077667	T	C	NA	-0.15	0.02	8.37E-13	51.19	0.01	0.03	0.71
MS	PsA	rs10801908	T	C	NA	-0.21	0.03	3.54E-16	66.48	-0.02	0.03	0.48
MS	PsA	rs1112718	G	A	NA	-0.11	0.02	2.46E-10	40.06	0.04	0.03	0.12
MS	PsA	rs11256593	T	C	NA	0.19	0.02	6.78E-27	115.29	-0.02	0.03	0.54
MS	PsA	rs114872782	T	C	NA	-2.45	0.26	7.72E-22	92.23	0.23	0.13	0.07
MS	PsA	rs11666263	G	A	NA	-0.10	0.02	1.20E-08	32.49	-0.07	0.03	0.01
MS	PsA	rs11711621	T	C	NA	-0.10	0.02	5.63E-08	29.49	-0.01	0.03	0.82
MS	PsA	rs11749040	A	G	NA	0.20	0.02	3.54E-17	71.02	-0.02	0.03	0.60
MS	PsA	rs1177228	G	A	NA	0.11	0.02	8.57E-09	33.14	-0.03	0.03	0.37
MS	PsA	rs11809700	T	C	NA	0.14	0.02	3.51E-15	61.96	0.00	0.03	0.99
MS	PsA	rs1204649	C	A	NA	-0.09	0.02	1.24E-07	27.95	0.07	0.03	0.01
MS	PsA	rs12147246	G	A	NA	-0.10	0.02	4.29E-09	34.49	0.01	0.03	0.83
MS	PsA	rs12365699	A	G	NA	-0.14	0.02	3.15E-10	39.58	0.07	0.03	0.03
MS	PsA	rs1250551	T	G	NA	0.12	0.02	2.66E-11	44.41	-0.07	0.03	0.01
MS	PsA	rs12612620	A	G	NA	0.21	0.04	4.26E-09	34.50	0.02	0.03	0.61
MS	PsA	rs12925972	C	T	NA	0.09	0.02	3.07E-08	30.66	-0.01	0.03	0.72

MS	PsA	rs13327021	T	C	NA	0.12	0.02	1.73E-11	45.26	-0.04	0.03	0.10
MS	PsA	rs140522	C	T	NA	-0.11	0.02	2.85E-10	39.78	-0.03	0.03	0.34
MS	PsA	rs1465697	T	C	NA	0.12	0.02	3.48E-11	43.89	0.05	0.03	0.14
MS	PsA	rs16822584	T	C	NA	-0.29	0.06	1.60E-07	27.47	-0.24	0.06	0.00
MS	PsA	rs17051321	T	C	NA	0.09	0.02	4.95E-07	25.28	-0.03	0.03	0.35
MS	PsA	rs17124032	A	G	NA	-0.22	0.03	7.08E-12	47.00	-0.03	0.05	0.61
MS	PsA	rs1738074	C	T	NA	0.11	0.02	9.91E-12	46.35	-0.04	0.03	0.08
MS	PsA	rs17724508	C	T	NA	-0.21	0.04	5.30E-08	29.60	0.05	0.06	0.35
MS	PsA	rs1860545	A	G	NA	0.12	0.02	7.79E-12	46.82	0.01	0.03	0.74
MS	PsA	rs2150879	A	G	NA	-0.10	0.02	3.29E-10	39.49	-0.03	0.03	0.19
MS	PsA	rs2242508	G	A	NA	-0.09	0.02	2.94E-07	26.29	-0.01	0.03	0.59
MS	PsA	rs2248461	A	G	NA	-0.11	0.02	5.33E-10	38.55	0.00	0.03	0.86
MS	PsA	rs2317231	T	G	NA	-0.10	0.02	1.90E-09	36.07	-0.03	0.03	0.27
MS	PsA	rs2681424	C	T	NA	-0.12	0.02	2.71E-13	53.41	-0.03	0.03	0.21
MS	PsA	rs28703878	G	A	NA	0.13	0.02	4.51E-10	38.88	0.01	0.03	0.82
MS	PsA	rs34695601	C	T	NA	-0.11	0.02	3.16E-08	30.60	-0.04	0.03	0.17
MS	PsA	rs354033	A	G	NA	-0.11	0.02	1.21E-08	32.48	-0.09	0.03	0.01
MS	PsA	rs35540610	C	T	NA	0.14	0.02	2.88E-12	48.77	0.04	0.03	0.25
MS	PsA	rs35703946	A	G	NA	-0.17	0.03	1.94E-09	36.03	-0.01	0.03	0.76
MS	PsA	rs3737798	G	A	NA	-0.09	0.02	1.40E-07	27.72	0.04	0.03	0.08

MS	PsA	rs3783196	A	C	NA	-0.08	0.02	4.34E-07	25.53	0.01	0.03	0.75
MS	PsA	rs3809627	A	C	NA	-0.10	0.02	3.25E-08	30.55	-0.03	0.03	0.24
MS	PsA	rs415759	C	T	NA	0.12	0.02	4.76E-08	29.81	0.03	0.04	0.47
MS	PsA	rs4325907	T	C	NA	-0.10	0.02	3.68E-09	34.78	0.00	0.03	0.86
MS	PsA	rs438613	C	T	NA	0.14	0.02	9.43E-17	69.08	-0.02	0.03	0.48
MS	PsA	rs478093	G	A	NA	0.11	0.02	4.30E-09	34.48	-0.06	0.03	0.04
MS	PsA	rs4796224	G	A	NA	0.09	0.02	1.62E-07	27.44	0.00	0.03	0.90
MS	PsA	rs4947255	T	C	NA	-0.55	0.06	2.54E-23	98.98	-0.29	0.06	0.00
MS	PsA	rs55970742	T	C	NA	-0.10	0.02	2.05E-08	31.45	0.03	0.03	0.25
MS	PsA	rs56272720	G	A	NA	0.48	0.09	1.63E-07	27.43	-0.03	0.07	0.65
MS	PsA	rs59655222	C	T	NA	-0.12	0.02	3.76E-11	43.74	0.00	0.03	0.98
MS	PsA	rs6012503	G	A	NA	0.09	0.02	1.62E-07	27.44	0.01	0.03	0.82
MS	PsA	rs6032662	T	C	NA	-0.13	0.02	2.85E-13	53.31	0.00	0.03	0.95
MS	PsA	rs60600003	G	T	NA	0.13	0.03	4.20E-07	25.60	0.14	0.04	0.00
MS	PsA	rs62195662	C	T	NA	0.49	0.09	2.47E-07	26.62	-0.06	0.04	0.17
MS	PsA	rs62420820	A	G	NA	0.14	0.02	2.50E-13	53.57	-0.02	0.03	0.45
MS	PsA	rs6564681	T	C	NA	-0.09	0.02	1.80E-07	27.24	0.06	0.03	0.03
MS	PsA	rs6670198	C	T	NA	-0.15	0.02	2.03E-16	67.57	-0.02	0.03	0.51
MS	PsA	rs67111717	G	A	NA	0.10	0.02	4.96E-07	25.28	0.06	0.03	0.02
MS	PsA	rs6742	C	T	NA	0.16	0.03	1.30E-07	27.87	0.04	0.03	0.17

MS	PsA	rs6763437	A	G	NA	-0.78	0.13	3.72E-09	34.76	0.06	0.14	0.64
MS	PsA	rs6837324	G	A	NA	0.09	0.02	3.16E-07	26.15	-0.02	0.03	0.44
MS	PsA	rs6990534	G	A	NA	0.11	0.02	3.60E-09	34.83	0.05	0.03	0.06
MS	PsA	rs701006	G	A	NA	0.11	0.02	1.35E-11	45.74	-0.02	0.03	0.38
MS	PsA	rs7190580	G	A	NA	-0.10	0.02	4.64E-08	29.86	-0.04	0.03	0.19
MS	PsA	rs7200146	T	G	NA	-0.17	0.02	7.00E-24	101.54	0.00	0.03	0.91
MS	PsA	rs72928038	A	G	NA	0.16	0.02	9.01E-11	42.02	-0.02	0.04	0.57
MS	PsA	rs7385730	T	G	NA	-0.12	0.02	3.99E-07	25.70	0.01	0.04	0.69
MS	PsA	rs743771	A	C	NA	-0.10	0.02	8.55E-10	37.63	-0.04	0.03	0.11
MS	PsA	rs74449127	G	A	NA	-0.20	0.03	1.36E-14	59.29	-0.02	0.03	0.40
MS	PsA	rs7592560	A	G	NA	0.10	0.02	2.87E-10	39.76	-0.05	0.03	0.08
MS	PsA	rs7855251	C	T	NA	-0.11	0.02	4.23E-08	30.04	-0.05	0.03	0.11
MS	PsA	rs7975763	T	C	NA	0.12	0.02	7.80E-09	33.32	-0.07	0.03	0.04
MS	PsA	rs9277647	T	C	NA	-0.21	0.02	5.47E-24	102.03	0.05	0.03	0.11
MS	PsA	rs9327104	G	A	NA	0.31	0.06	4.41E-07	25.50	-0.04	0.07	0.61
MS	PsA	rs9591325	C	T	NA	-0.21	0.03	4.16E-10	39.04	-0.17	0.05	0.00
MS	PsA	rs9878602	G	T	NA	-0.08	0.02	2.60E-07	26.52	-0.05	0.03	0.06
MS	PsA	rs9955954	G	A	NA	-0.11	0.02	1.54E-08	32.00	-0.04	0.03	0.18
MS	PsA	rs9992763	T	G	NA	-0.09	0.02	4.51E-08	29.91	-0.02	0.03	0.48
RA	PsA	rs10175798	A	G	NA	0.09	0.01	5.40E-09	38.70	0.03	0.03	0.21

RA	PsA	rs11217044	C	T	NA	-0.13	0.02	3.60E-15	51.70	0.05	0.03	0.12
RA	PsA	rs11574914	A	G	NA	0.11	0.02	2.10E-13	40.07	0.02	0.03	0.41
RA	PsA	rs11889341	T	C	NA	0.13	0.02	6.70E-19	55.45	0.03	0.03	0.31
RA	PsA	rs11933540	C	T	NA	0.14	0.02	8.80E-17	60.50	0.03	0.03	0.28
RA	PsA	rs13142500	C	T	NA	0.09	0.02	5.00E-09	30.40	0.02	0.03	0.39
RA	PsA	rs1571878	T	C	NA	-0.15	0.01	6.10E-30	165.34	-0.02	0.03	0.55
RA	PsA	rs1611236	A	G	NA	-0.12	0.01	2.10E-15	105.63	-0.06	0.03	0.04
RA	PsA	rs1858036	G	A	NA	-0.11	0.01	1.20E-14	66.93	-0.02	0.03	0.53
RA	PsA	rs2105325	C	A	NA	0.11	0.02	3.10E-10	37.10	0.01	0.03	0.82
RA	PsA	rs2233424	T	C	NA	0.23	0.03	7.60E-19	70.19	-0.01	0.05	0.85
RA	PsA	rs2235924	A	G	NA	-0.09	0.02	3.60E-09	32.48	0.02	0.03	0.51
RA	PsA	rs2301888	A	G	NA	-0.13	0.02	2.20E-18	55.86	-0.04	0.03	0.11
RA	PsA	rs2317230	T	G	NA	0.08	0.01	2.10E-08	30.31	-0.02	0.03	0.38
RA	PsA	rs2736337	C	T	NA	0.11	0.01	4.80E-12	84.44	0.05	0.03	0.11
RA	PsA	rs28411352	T	C	NA	0.11	0.01	3.60E-12	70.61	0.01	0.03	0.80
RA	PsA	rs3087243	A	G	NA	-0.14	0.02	1.70E-22	64.82	-0.02	0.03	0.41
RA	PsA	rs3784099	A	G	NA	-0.09	0.02	7.10E-10	32.48	0.03	0.03	0.36
RA	PsA	rs3806624	G	A	NA	0.08	0.01	1.90E-08	55.29	-0.01	0.03	0.66
RA	PsA	rs4239702	C	T	NA	0.12	0.01	9.00E-15	100.99	0.00	0.03	0.95
RA	PsA	rs4409785	C	T	NA	0.11	0.02	3.00E-08	37.10	0.00	0.03	0.98

RA	PsA	rs5019428	A	G	NA	0.09	0.01	7.20E-10	38.70	-0.03	0.03	0.30
RA	PsA	rs6712515	C	T	NA	-0.10	0.01	6.70E-15	55.73	-0.01	0.03	0.56
RA	PsA	rs6930468	G	A	NA	0.09	0.01	5.50E-11	69.18	0.03	0.03	0.28
RA	PsA	rs71508903	T	C	NA	0.16	0.02	2.30E-20	83.79	-0.03	0.03	0.30
RA	PsA	rs73013527	T	C	NA	-0.09	0.02	9.80E-11	32.48	-0.01	0.03	0.65
RA	PsA	rs73081554	T	C	NA	0.17	0.03	4.60E-08	31.69	0.07	0.05	0.16
RA	PsA	rs76153210	T	C	NA	0.17	0.03	2.20E-09	48.04	0.04	0.12	0.72
RA	PsA	rs8026898	A	G	NA	0.15	0.01	6.50E-19	129.80	0.01	0.03	0.74
RA	PsA	rs8032939	C	T	NA	0.12	0.02	4.80E-16	44.37	0.04	0.03	0.18
RA	PsA	rs9348832	A	G	NA	0.25	0.03	5.20E-19	68.80	0.06	0.06	0.36
RA	PsA	rs947474	A	G	NA	0.10	0.02	1.50E-08	33.38	0.00	0.03	0.97
Uveitis	PsA	rs117327462	A	C	0.02	0.92	0.19	7.31E-07	12625.93	-0.02	0.22	0.94
Uveitis	PsA	rs11907497	C	T	0.20	-0.17	0.03	7.24E-07	4309.06	0.00	0.03	0.93
Uveitis	PsA	rs144614916	C	A	0.02	0.79	0.12	2.85E-10	14442.40	0.27	0.10	0.01
Uveitis	PsA	rs145639523	T	C	0.03	0.68	0.07	5.52E-22	11536.13	0.18	0.06	0.00
Uveitis	PsA	rs148498636	G	A	0.05	0.72	0.15	2.22E-06	23521.20	-0.05	0.06	0.40
Uveitis	PsA	rs17315135	T	C	0.02	0.70	0.15	2.81E-06	10173.31	-0.14	0.17	0.40
Uveitis	PsA	rs189949127	T	G	0.01	0.93	0.20	4.44E-06	6744.28	-0.27	0.16	0.09
Uveitis	PsA	rs201276681	A	C	0.01	2.07	0.43	1.70E-06	36938.21	0.06	0.09	0.52
Uveitis	PsA	rs2223591	A	G	0.04	0.36	0.07	2.47E-07	4480.27	0.17	0.06	0.00

Uveitis	PsA	rs28366149	A	G	0.24	0.33	0.03	1.67E-21	19496.75	0.04	0.03	0.18
Uveitis	PsA	rs28432918	A	G	0.25	-0.18	0.03	3.55E-09	5996.51	-0.10	0.03	0.00
Uveitis	PsA	rs28892580	T	G	0.07	-0.29	0.05	1.34E-07	5070.29	-0.06	0.05	0.20
Uveitis	PsA	rs55679315	G	A	0.12	0.22	0.05	3.38E-06	4993.30	0.04	0.05	0.35
Uveitis	PsA	rs6138056	G	A	0.72	0.14	0.03	3.55E-06	3622.64	-0.04	0.03	0.16
Uveitis	PsA	rs62256214	A	C	0.02	0.46	0.10	2.20E-06	3359.75	0.12	0.08	0.14
Uveitis	PsA	rs62264596	C	A	0.05	0.38	0.08	3.72E-06	6672.30	0.07	0.08	0.38
Uveitis	PsA	rs685031	A	G	0.70	-0.35	0.03	2.20E-23	25704.84	0.01	0.03	0.58
Uveitis	PsA	rs73208917	T	C	0.02	0.59	0.13	3.94E-06	6719.79	0.01	0.12	0.94
Uveitis	PsA	rs79211491	C	A	0.08	0.22	0.04	6.93E-08	3488.60	-0.03	0.03	0.42
UC	PsA	rs10272963	T	C	NA	-0.15	0.02	4.11E-21	89.30	0.01	0.03	0.65
UC	PsA	rs10408351	A	G	NA	0.15	0.02	2.92E-14	57.58	0.07	0.03	0.05
UC	PsA	rs10761659	G	A	NA	0.13	0.02	1.33E-15	63.60	0.04	0.03	0.16
UC	PsA	rs10817678	A	G	NA	0.13	0.02	4.42E-15	61.39	0.04	0.03	0.13
UC	PsA	rs11209026	A	G	NA	-0.48	0.04	1.99E-41	182.02	-0.26	0.07	0.00
UC	PsA	rs1131095	C	T	NA	0.16	0.02	2.18E-21	89.91	0.00	0.03	0.99
UC	PsA	rs113846785	CG	C	NA	-0.16	0.02	1.15E-12	50.48	0.00	0.04	0.96
UC	PsA	rs113986290	T	C	NA	-0.31	0.05	7.59E-09	33.34	-0.08	0.09	0.38
UC	PsA	rs11651246	G	T	NA	0.15	0.02	2.01E-11	45.05	0.06	0.03	0.06
UC	PsA	rs12825700	A	G	NA	0.19	0.02	7.33E-32	137.66	0.01	0.03	0.70

UC	PsA	rs12936409	T	C	NA	0.14	0.02	5.62E-18	74.63	0.04	0.03	0.09
UC	PsA	rs1317209	A	G	NA	0.18	0.02	2.90E-19	80.20	-0.03	0.03	0.38
UC	PsA	rs13200059	A	G	NA	0.29	0.04	1.48E-11	45.59	0.18	0.06	0.00
UC	PsA	rs1359946	A	G	NA	0.16	0.02	6.58E-15	60.48	0.01	0.03	0.67
UC	PsA	rs137845	G	A	NA	0.10	0.02	1.50E-10	40.94	-0.02	0.03	0.47
UC	PsA	rs16940186	C	T	NA	0.14	0.02	2.18E-10	40.21	0.01	0.04	0.70
UC	PsA	rs1736161	A	G	NA	-0.12	0.02	2.22E-14	58.08	-0.01	0.03	0.76
UC	PsA	rs17656349	T	C	NA	0.09	0.02	1.54E-08	32.04	-0.05	0.03	0.07
UC	PsA	rs17715902	A	G	NA	0.10	0.02	4.62E-09	34.43	0.01	0.03	0.63
UC	PsA	rs2045241	A	G	NA	-0.11	0.02	2.83E-10	39.56	-0.02	0.03	0.44
UC	PsA	rs2212434	T	C	NA	0.13	0.02	2.80E-15	62.00	0.02	0.03	0.45
UC	PsA	rs2836881	T	G	NA	-0.22	0.02	1.11E-32	142.06	0.01	0.03	0.86
UC	PsA	rs28383224	G	A	NA	-0.15	0.02	4.65E-19	79.15	0.13	0.03	0.00
UC	PsA	rs3024493	A	C	NA	0.21	0.02	7.46E-24	100.95	0.11	0.03	0.00
UC	PsA	rs3734851	A	G	NA	0.50	0.06	6.58E-18	74.27	-0.46	0.12	0.00
UC	PsA	rs3812565	C	T	NA	0.13	0.02	6.50E-17	69.62	0.00	0.03	0.93
UC	PsA	rs3820330	A	C	NA	-0.16	0.02	3.91E-19	79.49	-0.04	0.03	0.15
UC	PsA	rs4676408	A	G	NA	0.14	0.02	1.19E-17	73.63	-0.02	0.03	0.45
UC	PsA	rs4728142	A	G	NA	0.10	0.02	3.23E-10	39.66	0.07	0.03	0.01
UC	PsA	rs4993442	T	G	NA	-0.10	0.02	3.54E-08	30.46	-0.01	0.03	0.84

UC	PsA	rs55905347	A	G	NA	0.11	0.02	2.09E-10	40.31	-0.02	0.03	0.37
UC	PsA	rs56062135	T	C	NA	0.11	0.02	4.66E-09	34.32	0.02	0.03	0.60
UC	PsA	rs6017342	C	A	NA	0.19	0.02	3.95E-30	130.76	0.04	0.03	0.13
UC	PsA	rs6062496	A	G	NA	0.14	0.02	8.97E-17	69.51	-0.02	0.03	0.54
UC	PsA	rs62180181	T	C	NA	0.12	0.02	8.08E-13	51.40	0.03	0.03	0.21
UC	PsA	rs67111717	G	A	NA	0.09	0.02	3.27E-08	30.47	0.06	0.03	0.02
UC	PsA	rs6889364	A	G	NA	0.13	0.02	7.87E-09	33.42	-0.02	0.03	0.64
UC	PsA	rs6933404	C	T	NA	0.15	0.02	2.69E-15	62.47	-0.04	0.03	0.22
UC	PsA	rs72704802	T	C	NA	-0.12	0.02	2.89E-09	35.25	-0.06	0.04	0.10
UC	PsA	rs7523335	A	G	NA	-0.14	0.02	3.42E-11	43.75	0.03	0.03	0.30
UC	PsA	rs7554511	A	C	NA	-0.14	0.02	4.27E-16	66.17	0.00	0.03	0.93
UC	PsA	rs7608697	C	A	NA	0.16	0.02	3.03E-23	98.39	0.05	0.03	0.04
UC	PsA	rs78064630	A	G	NA	0.18	0.03	1.08E-08	32.61	-0.17	0.06	0.00
UC	PsA	rs79051659	A	G	NA	0.16	0.03	1.30E-09	36.96	0.05	0.05	0.29
UC	PsA	rs7911117	G	T	NA	-0.13	0.02	1.84E-08	31.53	0.04	0.04	0.32
UC	PsA	rs7911680	C	A	NA	-0.15	0.02	6.71E-22	91.99	0.02	0.03	0.50
UC	PsA	rs798506	C	T	NA	-0.12	0.02	1.47E-11	45.39	-0.01	0.03	0.79
UC	PsA	rs9611131	C	T	NA	-0.15	0.02	5.11E-11	43.31	0.06	0.04	0.13
UC	PsA	rs989960	T	C	NA	-0.12	0.02	3.28E-14	57.57	0.00	0.03	0.93
Vitiligo	PsA	rs10200159	C	T	NA	0.18	0.05	3.73E-19	98.10	0.03	0.07	0.64

Vitiligo	PsA	rs1031034	A	C	NA	-0.07	0.03	3.43E-08	77.75	0.01	0.03	0.67
Vitiligo	PsA	rs1043101	G	A	NA	0.09	0.02	5.26E-18	169.01	-0.01	0.03	0.76
Vitiligo	PsA	rs10986311	C	T	NA	0.06	0.02	1.01E-08	73.51	0.06	0.03	0.02
Vitiligo	PsA	rs11021232	C	T	NA	0.13	0.03	2.10E-23	228.11	0.00	0.03	0.90
Vitiligo	PsA	rs1126809	A	G	NA	-0.17	0.03	1.16E-43	511.29	-0.04	0.03	0.19
Vitiligo	PsA	rs117744081	G	A	NA	0.26	0.06	8.72E-26	186.11	0.17	0.07	0.01
Vitiligo	PsA	rs12203592	T	C	NA	-0.10	0.04	2.95E-10	95.56	0.06	0.07	0.41
Vitiligo	PsA	rs12421615	A	G	NA	-0.06	0.03	4.81E-08	74.17	-0.07	0.03	0.01
Vitiligo	PsA	rs12771452	A	G	NA	-0.09	0.03	4.43E-12	124.47	0.00	0.03	0.95
Vitiligo	PsA	rs13076312	T	C	NA	0.12	0.02	1.61E-30	323.65	0.05	0.03	0.05
Vitiligo	PsA	rs13136820	C	T	NA	0.07	0.03	3.60E-11	102.29	-0.01	0.03	0.85
Vitiligo	PsA	rs1635168	A	C	NA	0.14	0.04	8.78E-14	156.59	0.02	0.12	0.89
Vitiligo	PsA	rs16843742	C	T	NA	-0.08	0.03	1.02E-09	101.22	-0.03	0.03	0.30
Vitiligo	PsA	rs2247314	C	T	NA	-0.10	0.03	1.72E-18	218.69	-0.02	0.03	0.57
Vitiligo	PsA	rs229527	A	C	NA	0.12	0.02	1.14E-30	316.27	0.05	0.03	0.05
Vitiligo	PsA	rs2304206	A	G	NA	-0.08	0.03	2.36E-09	97.73	-0.03	0.03	0.38
Vitiligo	PsA	rs231725	A	G	NA	0.07	0.03	1.49E-10	99.28	-0.01	0.03	0.64
Vitiligo	PsA	rs34346645	A	C	NA	-0.10	0.03	7.99E-19	205.33	-0.05	0.03	0.06
Vitiligo	PsA	rs35860234	G	T	NA	0.07	0.03	4.76E-09	81.92	0.03	0.03	0.32
Vitiligo	PsA	rs41342147	A	G	NA	-0.10	0.04	3.70E-09	84.97	0.05	0.05	0.30

Vitiligo	PsA	rs4268748	C	T	NA	-0.15	0.03	2.88E-33	347.02	-0.05	0.03	0.10
Vitiligo	PsA	rs4308124	C	T	NA	0.06	0.02	3.96E-09	80.01	-0.02	0.03	0.43
Vitiligo	PsA	rs4807000	A	G	NA	0.08	0.02	1.94E-14	146.64	0.03	0.03	0.23
Vitiligo	PsA	rs561079	C	T	NA	0.06	0.02	3.76E-08	67.13	0.01	0.03	0.68
Vitiligo	PsA	rs6012953	G	A	NA	0.06	0.02	9.47E-09	81.50	0.05	0.03	0.05
Vitiligo	PsA	rs706779	C	T	NA	-0.11	0.02	7.20E-27	286.59	-0.05	0.03	0.05
Vitiligo	PsA	rs71508903	T	C	NA	0.07	0.03	6.93E-09	78.56	-0.03	0.03	0.30
Vitiligo	PsA	rs72928038	A	G	NA	0.10	0.03	1.00E-14	141.18	-0.02	0.04	0.57
Vitiligo	PsA	rs78037977	G	A	NA	0.12	0.03	6.74E-17	134.65	0.04	0.04	0.29
Vitiligo	PsA	rs78521699	G	A	NA	-0.10	0.04	2.54E-08	102.55	-0.02	0.03	0.54
Vitiligo	PsA	rs8083511	C	A	NA	0.08	0.03	2.81E-10	101.83	-0.01	0.03	0.68
Vitiligo	PsA	rs8192917	C	T	NA	0.10	0.03	8.91E-16	155.39	-0.03	0.03	0.37
BP	PsA	rs1394944	A	C	0.04	0.59	0.13	4.60E-06	10815.23	0.00	0.05	0.98
BP	PsA	rs1688134	G	A	0.60	-0.29	0.06	2.02E-06	16360.36	0.02	0.03	0.58
BP	PsA	rs241430	C	T	0.71	-0.30	0.07	3.65E-06	14597.25	0.11	0.03	0.00
BP	PsA	rs76965871	A	G	0.01	1.05	0.21	6.08E-07	9472.05	0.15	0.10	0.10
BP	PsA	rs9270891	G	T	0.61	0.44	0.06	4.98E-12	38629.01	0.38	0.11	0.00
AS	PsA	rs11065898	T	C	NA	0.03	0.00	4.71E-08	29.83	0.02	0.03	0.44
AS	PsA	rs1128905	C	T	NA	-0.02	0.00	6.95E-09	33.55	0.02	0.03	0.51
AS	PsA	rs11624293	C	T	NA	0.04	0.01	1.49E-10	41.04	0.01	0.05	0.81

AS	PsA	rs12615545	C	T	NA	0.03	0.00	1.03E-09	37.26	0.03	0.03	0.25
AS	PsA	rs1801274	G	A	NA	0.03	0.00	1.35E-09	36.74	0.04	0.03	0.08
AS	PsA	rs1860545	A	G	NA	-0.03	0.00	2.78E-10	39.82	0.01	0.03	0.74
AS	PsA	rs2517655	T	C	NA	0.09	0.00	3.47E-80	359.55	0.09	0.03	0.00
AS	PsA	rs2531875	T	G	NA	-0.03	0.00	1.22E-10	41.43	-0.02	0.03	0.38
AS	PsA	rs2836883	A	G	NA	-0.04	0.00	6.46E-17	69.83	0.01	0.03	0.86
AS	PsA	rs4129267	T	C	NA	-0.03	0.00	3.32E-13	53.00	-0.07	0.03	0.01
AS	PsA	rs41299637	G	T	NA	-0.04	0.00	1.81E-15	63.25	0.00	0.03	0.94
AS	PsA	rs4676410	A	G	NA	0.03	0.00	9.90E-09	32.86	0.02	0.03	0.41
AS	PsA	rs7191548	C	T	NA	0.02	0.00	5.51E-09	34.00	0.02	0.03	0.45
HT	PsA	rs11611029	T	C	0.39	-0.10	0.02	1.16E-08	110.96	-0.02	0.03	0.35
HT	PsA	rs2110451	A	G	0.38	0.10	0.02	3.33E-08	363.08	0.05	0.03	0.09
HT	PsA	rs34636506	G	A	0.37	-0.13	0.02	1.92E-14	74.90	-0.02	0.03	0.44
HT	PsA	rs6679677	A	C	0.11	0.33	0.02	5.37E-44	831.95	0.09	0.04	0.01
HT	PsA	rs7030280	T	C	0.75	0.21	0.02	6.44E-31	15.85	-0.01	0.03	0.72
HT	PsA	rs73192661	T	C	0.43	-0.10	0.02	1.45E-09	306.18	-0.04	0.03	0.12
HT	PsA	rs9357119	T	G	0.38	-0.15	0.02	3.65E-14	420.44	-0.05	0.03	0.07
CD	PsA	rs10114470	C	T	NA	0.17	0.02	1.76E-21	90.84	0.03	0.03	0.29
CD	PsA	rs10884966	A	G	NA	0.11	0.02	4.13E-11	43.74	0.02	0.03	0.42
CD	PsA	rs11236797	A	C	NA	0.18	0.02	8.51E-28	119.50	0.02	0.03	0.44

CD	PsA	rs112856973	C	T	NA	-0.16	0.02	3.61E-11	44.00	0.00	0.03	0.95
CD	PsA	rs1148246	T	C	NA	-0.13	0.02	2.09E-15	62.76	0.02	0.03	0.50
CD	PsA	rs11677002	C	T	NA	-0.11	0.02	4.57E-12	47.55	-0.01	0.03	0.65
CD	PsA	rs1250573	A	G	NA	-0.15	0.02	1.92E-17	72.29	-0.05	0.03	0.08
CD	PsA	rs12936409	T	C	NA	0.14	0.02	4.31E-19	79.43	0.04	0.03	0.09
CD	PsA	rs1297264	G	A	NA	-0.18	0.02	1.59E-27	117.78	-0.01	0.03	0.77
CD	PsA	rs13107325	T	C	NA	0.20	0.03	1.66E-12	49.89	0.01	0.11	0.92
CD	PsA	rs1321859	T	C	NA	-0.10	0.02	1.18E-09	37.19	-0.01	0.03	0.84
CD	PsA	rs1373904	G	A	NA	0.14	0.02	9.11E-14	55.65	0.03	0.03	0.32
CD	PsA	rs145568234	G	T	NA	0.86	0.06	4.31E-42	184.66	-0.12	0.21	0.56
CD	PsA	rs181826	A	C	NA	0.12	0.02	3.24E-12	48.41	0.03	0.03	0.21
CD	PsA	rs194746	T	C	NA	0.10	0.02	1.24E-09	36.67	0.00	0.03	0.88
CD	PsA	rs2002695	G	A	NA	-0.13	0.02	8.31E-12	46.80	-0.05	0.03	0.09
CD	PsA	rs2076756	G	A	NA	0.39	0.02	1.80E-108	489.55	0.01	0.04	0.83
CD	PsA	rs2110735	G	A	NA	-0.14	0.02	1.20E-13	55.00	0.01	0.03	0.69
CD	PsA	rs212408	T	G	NA	-0.11	0.02	9.12E-12	46.27	-0.04	0.03	0.15
CD	PsA	rs2143178	C	T	NA	-0.21	0.02	6.84E-21	87.58	0.05	0.04	0.20
CD	PsA	rs2284553	G	A	NA	0.13	0.02	1.14E-14	59.90	0.02	0.03	0.40
CD	PsA	rs28999107	T	G	NA	0.11	0.02	1.06E-09	37.02	-0.02	0.03	0.39
CD	PsA	rs2948542	G	A	NA	0.10	0.02	5.15E-10	38.85	-0.01	0.03	0.84

CD	PsA	rs34004493	G	A	NA	0.13	0.02	2.00E-12	49.39	0.01	0.03	0.69
CD	PsA	rs34635748	T	C	NA	0.48	0.05	1.95E-21	90.47	0.00	0.08	0.98
CD	PsA	rs3761158	A	G	NA	-0.11	0.02	2.65E-11	44.28	0.00	0.03	0.93
CD	PsA	rs3816234	A	G	NA	0.27	0.02	1.51E-62	278.59	-0.02	0.03	0.44
CD	PsA	rs4077515	T	C	NA	0.18	0.02	3.14E-30	130.12	0.00	0.03	0.88
CD	PsA	rs42861	G	A	NA	0.12	0.02	8.87E-14	55.40	0.03	0.03	0.27
CD	PsA	rs4380956	A	G	NA	0.13	0.02	1.15E-15	64.00	-0.02	0.03	0.53
CD	PsA	rs4807570	A	G	NA	0.18	0.02	6.03E-21	88.04	0.03	0.03	0.32
CD	PsA	rs492602	G	A	NA	0.11	0.02	2.33E-11	44.77	0.04	0.03	0.12
CD	PsA	rs55946629	A	C	NA	0.18	0.02	2.85E-14	57.72	-0.01	0.05	0.75
CD	PsA	rs56116661	T	C	NA	-0.13	0.02	5.67E-10	38.30	-0.03	0.03	0.30
CD	PsA	rs5754100	C	T	NA	0.17	0.02	3.02E-16	67.06	0.04	0.03	0.12
CD	PsA	rs59926756	A	G	NA	0.11	0.02	1.74E-09	36.41	-0.03	0.03	0.24
CD	PsA	rs6062496	A	G	NA	0.12	0.02	2.62E-13	53.63	-0.02	0.03	0.54
CD	PsA	rs62126620	A	G	NA	0.14	0.02	8.61E-13	51.32	0.00	0.04	1.00
CD	PsA	rs62324212	A	C	NA	0.11	0.02	8.02E-11	42.29	0.03	0.03	0.29
CD	PsA	rs6451494	C	T	NA	0.26	0.02	8.26E-56	246.25	0.00	0.03	0.86
CD	PsA	rs6579807	T	C	NA	0.20	0.02	3.44E-16	66.71	-0.01	0.04	0.77
CD	PsA	rs6584282	G	A	NA	-0.17	0.02	3.44E-25	107.38	0.02	0.03	0.45
CD	PsA	rs6704109	T	C	NA	0.17	0.02	5.10E-22	93.26	-0.01	0.03	0.66

CD	PsA	rs6740847	G	A	NA	-0.10	0.02	9.72E-11	41.72	0.01	0.03	0.57
CD	PsA	rs6873866	C	T	NA	-0.13	0.02	1.35E-15	64.19	-0.02	0.03	0.44
CD	PsA	rs72743461	A	C	NA	0.17	0.02	2.26E-19	81.09	0.01	0.03	0.70
CD	PsA	rs72798422	C	T	NA	0.55	0.04	6.05E-47	206.91	0.06	0.07	0.36
CD	PsA	rs744166	G	A	NA	-0.11	0.02	1.80E-12	49.69	-0.03	0.03	0.28
CD	PsA	rs7608697	C	A	NA	0.12	0.02	4.03E-14	56.85	0.05	0.03	0.04
CD	PsA	rs77566919	A	G	NA	-0.11	0.02	4.13E-09	34.65	-0.03	0.03	0.37
CD	PsA	rs79832570	C	T	NA	0.22	0.03	8.90E-11	42.17	0.03	0.05	0.51
CD	PsA	rs9482770	C	T	NA	0.10	0.02	1.01E-09	37.12	0.04	0.03	0.16
CD	PsA	rs9656588	C	T	NA	0.12	0.02	8.73E-12	46.76	0.04	0.03	0.14
CD	PsA	rs9836291	A	G	NA	0.17	0.02	3.77E-24	102.60	0.00	0.03	0.97

Abbreviations: PsA, psoriatic arthritis; SLE, Systemic lupus erythematosus; MS, multiple sclerosis; RA, Rheumatoid arthritis; CD, Crohn's disease; UC, Ulcerative colitis; BP, Bullous pemphigoid; AS, Ankylosing spondylitis; HT, Hashimoto thyroiditis; AIDs, Autoimmune diseases.

Table S10. Detailed information of instrumental variables utilized in the Mendelian randomization analysis of AIDs on PsO.

						**Exposure**				**Outcome**		
**Exposure**	**Outcome**	**SNP**	**effect allele**	**other allele**	**Effect allele frequency**	**Beta**	**Se**	**P-value**	**F-statistics**	**Beta**	**SE**	**P-value**
SLE	PsO	rs10048743	T	G	NA	-0.23	0.04	2.04E-08	31.45	-0.03	0.02	0.12
SLE	PsO	rs10200680	T	C	NA	-0.25	0.04	4.96E-09	34.20	0.01	0.02	0.63

SLE	PsO	rs1078324	A	C	NA	-0.71	0.08	7.11E-20	83.27	0.03	0.04	0.35
SLE	PsO	rs10912578	G	A	NA	-0.25	0.03	1.65E-15	63.44	0.01	0.02	0.55
SLE	PsO	rs1143679	A	G	NA	0.58	0.04	5.03E-48	211.97	-0.04	0.02	0.08
SLE	PsO	rs12094036	C	T	NA	-0.33	0.06	1.37E-08	32.23	-0.03	0.03	0.26
SLE	PsO	rs12524498	T	G	NA	-0.67	0.12	2.48E-08	31.07	-0.03	0.05	0.52
SLE	PsO	rs13019891	T	G	NA	-0.56	0.03	1.65E-83	374.80	-0.01	0.02	0.70
SLE	PsO	rs13136219	T	C	NA	-0.17	0.03	3.50E-10	39.37	-0.01	0.02	0.64
SLE	PsO	rs13332649	G	A	NA	-0.31	0.04	5.43E-17	70.16	-0.02	0.02	0.32
SLE	PsO	rs1464446	T	G	NA	-0.33	0.04	2.79E-16	66.94	-0.01	0.02	0.69
SLE	PsO	rs150180633	T	C	NA	0.93	0.07	2.66E-41	181.17	-0.09	0.12	0.43
SLE	PsO	rs17849501	T	C	NA	0.81	0.05	1.81E-59	264.44	0.04	0.04	0.34
SLE	PsO	rs2431697	C	T	NA	-0.22	0.03	2.60E-14	58.01	-0.03	0.02	0.06
SLE	PsO	rs2459611	T	C	NA	0.26	0.05	7.62E-09	33.37	-0.01	0.03	0.82
SLE	PsO	rs2573219	C	A	NA	0.59	0.04	1.13E-42	187.44	-0.01	0.03	0.72
SLE	PsO	rs268124	T	C	NA	0.19	0.03	8.60E-09	33.13	0.01	0.02	0.44
SLE	PsO	rs34703115	C	T	NA	-0.62	0.10	4.08E-09	34.58	0.01	0.04	0.75
SLE	PsO	rs35000415	T	C	NA	0.59	0.04	1.86E-45	200.20	0.03	0.02	0.15
SLE	PsO	rs353608	G	A	NA	0.19	0.03	2.93E-11	44.22	0.01	0.01	0.51
SLE	PsO	rs4274624	T	C	NA	-0.56	0.03	9.73E-66	57.81	-0.01	0.02	0.60
SLE	PsO	rs4661543	G	T	NA	0.27	0.04	9.40E-11	41.94	0.01	0.03	0.69

SLE	PsO	rs4916215	T	C	NA	0.22	0.03	5.07E-11	43.15	-0.01	0.02	0.67
SLE	PsO	rs6671847	A	G	NA	0.20	0.03	6.64E-12	47.12	0.00	0.02	0.93
SLE	PsO	rs6679677	A	C	NA	0.34	0.05	4.55E-13	52.38	0.03	0.02	0.20
SLE	PsO	rs6889239	C	T	NA	0.28	0.03	2.19E-18	76.50	0.03	0.02	0.05
SLE	PsO	rs7097397	A	G	NA	-0.19	0.03	8.60E-11	42.11	0.02	0.02	0.29
SLE	PsO	rs73050535	T	C	NA	-0.71	0.12	9.11E-09	33.02	-0.13	0.10	0.22
SLE	PsO	rs73068668	A	G	NA	-0.31	0.06	4.40E-08	29.96	-0.04	0.03	0.12
SLE	PsO	rs7768653	T	C	NA	-0.21	0.03	3.11E-12	48.61	0.01	0.02	0.49
SLE	PsO	rs7823055	T	G	NA	-0.35	0.03	1.64E-34	150.09	-0.01	0.02	0.71
SLE	PsO	rs9852014	G	A	NA	0.62	0.05	2.26E-36	158.61	-0.01	0.03	0.62
MS	PsO	rs10063294	A	G	NA	-0.10	0.02	1.13E-09	37.09	-0.04	0.02	0.00
MS	PsO	rs1014486	C	T	NA	0.11	0.02	1.36E-10	41.21	-0.01	0.02	0.48
MS	PsO	rs1026916	G	A	NA	-0.13	0.02	1.02E-13	55.33	-0.02	0.02	0.11
MS	PsO	rs1077667	T	C	NA	-0.15	0.02	8.37E-13	51.19	0.02	0.02	0.14
MS	PsO	rs10801908	T	C	NA	-0.21	0.03	3.54E-16	66.48	-0.04	0.02	0.02
MS	PsO	rs1112718	G	A	NA	-0.11	0.02	2.46E-10	40.06	0.02	0.02	0.23
MS	PsO	rs11256593	T	C	NA	0.19	0.02	6.78E-27	115.29	-0.01	0.01	0.67
MS	PsO	rs114872782	T	C	NA	-2.45	0.26	7.72E-22	92.23	0.29	0.07	0.00
MS	PsO	rs11666263	G	A	NA	-0.10	0.02	1.20E-08	32.49	-0.03	0.02	0.04
MS	PsO	rs11711621	T	C	NA	-0.10	0.02	5.63E-08	29.49	0.02	0.02	0.25

MS	PsO	rs11749040	A	G	NA	0.20	0.02	3.54E-17	71.02	0.04	0.02	0.02
MS	PsO	rs1177228	G	A	NA	0.11	0.02	8.57E-09	33.14	0.00	0.02	0.84
MS	PsO	rs11809700	T	C	NA	0.14	0.02	3.51E-15	61.96	-0.02	0.02	0.18
MS	PsO	rs1204649	C	A	NA	-0.09	0.02	1.24E-07	27.95	0.03	0.02	0.06
MS	PsO	rs12147246	G	A	NA	-0.10	0.02	4.29E-09	34.49	0.00	0.02	0.97
MS	PsO	rs12365699	A	G	NA	-0.14	0.02	3.15E-10	39.58	0.01	0.02	0.67
MS	PsO	rs12612620	A	G	NA	0.21	0.04	4.26E-09	34.50	0.02	0.02	0.32
MS	PsO	rs12925972	C	T	NA	0.09	0.02	3.07E-08	30.66	-0.01	0.02	0.54
MS	PsO	rs13327021	T	C	NA	0.12	0.02	1.73E-11	45.26	-0.02	0.02	0.25
MS	PsO	rs140522	C	T	NA	-0.11	0.02	2.85E-10	39.78	-0.02	0.02	0.17
MS	PsO	rs1465697	T	C	NA	0.12	0.02	3.48E-11	43.89	0.04	0.02	0.06
MS	PsO	rs17051321	T	C	NA	0.09	0.02	4.95E-07	25.28	-0.06	0.02	0.00
MS	PsO	rs17124032	A	G	NA	-0.22	0.03	7.08E-12	47.00	-0.03	0.03	0.40
MS	PsO	rs1738074	C	T	NA	0.11	0.02	9.91E-12	46.35	-0.04	0.02	0.01
MS	PsO	rs17724508	C	T	NA	-0.21	0.04	5.30E-08	29.60	0.04	0.03	0.18
MS	PsO	rs1860545	A	G	NA	0.12	0.02	7.79E-12	46.82	0.01	0.02	0.54
MS	PsO	rs2150879	A	G	NA	-0.10	0.02	3.29E-10	39.49	-0.02	0.01	0.31
MS	PsO	rs2242508	G	A	NA	-0.09	0.02	2.94E-07	26.29	0.00	0.02	0.82
MS	PsO	rs2248461	A	G	NA	-0.11	0.02	5.33E-10	38.55	0.00	0.02	0.90
MS	PsO	rs2317231	T	G	NA	-0.10	0.02	1.90E-09	36.07	0.02	0.02	0.29

MS	PsO	rs2681424	C	T	NA	-0.12	0.02	2.71E-13	53.41	0.00	0.01	0.97
MS	PsO	rs28703878	G	A	NA	0.13	0.02	4.51E-10	38.88	0.01	0.02	0.53
MS	PsO	rs34695601	C	T	NA	-0.11	0.02	3.16E-08	30.60	0.00	0.02	0.95
MS	PsO	rs354033	A	G	NA	-0.11	0.02	1.21E-08	32.48	-0.06	0.02	0.00
MS	PsO	rs35540610	C	T	NA	0.14	0.02	2.88E-12	48.77	0.07	0.02	0.00
MS	PsO	rs35703946	A	G	NA	-0.17	0.03	1.94E-09	36.03	0.00	0.02	0.93
MS	PsO	rs3737798	G	A	NA	-0.09	0.02	1.40E-07	27.72	0.02	0.02	0.20
MS	PsO	rs3783196	A	C	NA	-0.08	0.02	4.34E-07	25.53	0.01	0.02	0.48
MS	PsO	rs3809627	A	C	NA	-0.10	0.02	3.25E-08	30.55	-0.02	0.01	0.10
MS	PsO	rs415759	C	T	NA	0.12	0.02	4.76E-08	29.81	0.00	0.02	0.99
MS	PsO	rs4325907	T	C	NA	-0.10	0.02	3.68E-09	34.78	-0.01	0.02	0.57
MS	PsO	rs438613	C	T	NA	0.14	0.02	9.43E-17	69.08	0.00	0.02	0.74
MS	PsO	rs478093	G	A	NA	0.11	0.02	4.30E-09	34.48	-0.01	0.02	0.39
MS	PsO	rs4796224	G	A	NA	0.09	0.02	1.62E-07	27.44	0.01	0.02	0.57
MS	PsO	rs4947255	T	C	NA	-0.55	0.06	2.54E-23	98.98	-0.13	0.04	0.00
MS	PsO	rs55970742	T	C	NA	-0.10	0.02	2.05E-08	31.45	0.00	0.02	0.77
MS	PsO	rs56272720	G	A	NA	0.48	0.09	1.63E-07	27.43	-0.03	0.04	0.51
MS	PsO	rs59655222	C	T	NA	-0.12	0.02	3.76E-11	43.74	0.00	0.02	0.85
MS	PsO	rs6012503	G	A	NA	0.09	0.02	1.62E-07	27.44	0.00	0.02	0.91
MS	PsO	rs6032662	T	C	NA	-0.13	0.02	2.85E-13	53.31	-0.03	0.02	0.09

MS	PsO	rs62195662	C	T	NA	0.49	0.09	2.47E-07	26.62	0.00	0.03	0.87
MS	PsO	rs62420820	A	G	NA	0.14	0.02	2.50E-13	53.57	0.01	0.02	0.62
MS	PsO	rs6564681	T	C	NA	-0.09	0.02	1.80E-07	27.24	0.05	0.02	0.00
MS	PsO	rs6670198	C	T	NA	-0.15	0.02	2.03E-16	67.57	-0.01	0.02	0.52
MS	PsO	rs6742	C	T	NA	0.16	0.03	1.30E-07	27.87	0.01	0.02	0.61
MS	PsO	rs6763437	A	G	NA	-0.78	0.13	3.72E-09	34.76	0.19	0.08	0.02
MS	PsO	rs6837324	G	A	NA	0.09	0.02	3.16E-07	26.15	-0.01	0.02	0.53
MS	PsO	rs6990534	G	A	NA	0.11	0.02	3.60E-09	34.83	0.01	0.02	0.41
MS	PsO	rs701006	G	A	NA	0.11	0.02	1.35E-11	45.74	0.00	0.02	0.77
MS	PsO	rs7190580	G	A	NA	-0.10	0.02	4.64E-08	29.86	-0.06	0.02	0.00
MS	PsO	rs7200146	T	G	NA	-0.17	0.02	7.00E-24	101.54	0.02	0.02	0.13
MS	PsO	rs72928038	A	G	NA	0.16	0.02	9.01E-11	42.02	-0.01	0.02	0.64
MS	PsO	rs7385730	T	G	NA	-0.12	0.02	3.99E-07	25.70	0.01	0.02	0.68
MS	PsO	rs743771	A	C	NA	-0.10	0.02	8.55E-10	37.63	0.02	0.01	0.16
MS	PsO	rs74449127	G	A	NA	-0.20	0.03	1.36E-14	59.29	-0.01	0.02	0.46
MS	PsO	rs7592560	A	G	NA	0.10	0.02	2.87E-10	39.76	0.00	0.01	0.83
MS	PsO	rs7855251	C	T	NA	-0.11	0.02	4.23E-08	30.04	-0.06	0.02	0.00
MS	PsO	rs7975763	T	C	NA	0.12	0.02	7.80E-09	33.32	-0.03	0.02	0.17
MS	PsO	rs9277647	T	C	NA	-0.21	0.02	5.47E-24	102.03	0.04	0.02	0.06
MS	PsO	rs9327104	G	A	NA	0.31	0.06	4.41E-07	25.50	0.03	0.04	0.46

MS	PsO	rs9591325	C	T	NA	-0.21	0.03	4.16E-10	39.04	-0.13	0.03	0.00
MS	PsO	rs9878602	G	T	NA	-0.08	0.02	2.60E-07	26.52	-0.02	0.01	0.22
MS	PsO	rs9955954	G	A	NA	-0.11	0.02	1.54E-08	32.00	-0.01	0.02	0.66
MS	PsO	rs9992763	T	G	NA	-0.09	0.02	4.51E-08	29.91	0.00	0.01	0.89
RA	PsO	rs10175798	A	G	NA	0.09	0.01	5.40E-09	38.70	0.01	0.02	0.71
RA	PsO	rs11217044	C	T	NA	-0.13	0.02	3.60E-15	51.70	0.00	0.02	0.87
RA	PsO	rs112733823	T	C	NA	0.29	0.02	6.60E-39	229.39	-0.06	0.02	0.00
RA	PsO	rs11574914	A	G	NA	0.11	0.02	2.10E-13	40.07	0.03	0.02	0.11
RA	PsO	rs11889341	T	C	NA	0.13	0.02	6.70E-19	55.45	0.01	0.02	0.66
RA	PsO	rs11933540	C	T	NA	0.14	0.02	8.80E-17	60.50	0.02	0.02	0.16
RA	PsO	rs12126142	A	G	NA	-0.08	0.02	3.50E-09	25.94	-0.05	0.02	0.00
RA	PsO	rs13142500	C	T	NA	0.09	0.02	5.00E-09	30.40	0.00	0.02	0.91
RA	PsO	rs1571878	T	C	NA	-0.15	0.01	6.10E-30	165.34	0.02	0.02	0.20
RA	PsO	rs1611236	A	G	NA	-0.12	0.01	2.10E-15	105.63	-0.03	0.02	0.06
RA	PsO	rs1858036	G	A	NA	-0.11	0.01	1.20E-14	66.93	0.01	0.02	0.63
RA	PsO	rs1893592	C	A	NA	-0.10	0.01	3.70E-12	55.73	0.03	0.02	0.06
RA	PsO	rs1953126	C	T	NA	-0.09	0.01	1.00E-09	36.63	-0.01	0.02	0.70
RA	PsO	rs2105325	C	A	NA	0.11	0.02	3.10E-10	37.10	-0.01	0.02	0.78
RA	PsO	rs212389	A	G	NA	0.10	0.02	3.30E-10	27.35	-0.07	0.02	0.00
RA	PsO	rs2233424	T	C	NA	0.23	0.03	7.60E-19	70.19	-0.05	0.03	0.12

RA	PsO	rs2235924	A	G	NA	-0.09	0.02	3.60E-09	32.48	0.00	0.02	0.95
RA	PsO	rs2301888	A	G	NA	-0.13	0.02	2.20E-18	55.86	0.00	0.02	0.81
RA	PsO	rs2317230	T	G	NA	0.08	0.01	2.10E-08	30.31	0.02	0.02	0.21
RA	PsO	rs2561477	A	G	NA	-0.08	0.01	1.90E-09	57.74	-0.04	0.02	0.01
RA	PsO	rs2736337	C	T	NA	0.11	0.01	4.80E-12	84.44	0.03	0.02	0.08
RA	PsO	rs28411352	T	C	NA	0.11	0.01	3.60E-12	70.61	-0.01	0.02	0.48
RA	PsO	rs3087243	A	G	NA	-0.14	0.02	1.70E-22	64.82	-0.01	0.02	0.38
RA	PsO	rs3778753	G	A	NA	0.11	0.02	1.10E-14	37.10	0.04	0.02	0.00
RA	PsO	rs3784099	A	G	NA	-0.09	0.02	7.10E-10	32.48	0.02	0.02	0.19
RA	PsO	rs3806624	G	A	NA	0.08	0.01	1.90E-08	55.29	-0.02	0.02	0.28
RA	PsO	rs4239702	C	T	NA	0.12	0.01	9.00E-15	100.99	-0.03	0.02	0.07
RA	PsO	rs4409785	C	T	NA	0.11	0.02	3.00E-08	37.10	-0.02	0.02	0.22
RA	PsO	rs5019428	A	G	NA	0.09	0.01	7.20E-10	38.70	-0.04	0.02	0.01
RA	PsO	rs6679677	A	C	NA	0.59	0.02	2.10E-149	722.96	0.03	0.02	0.20
RA	PsO	rs6712515	C	T	NA	-0.10	0.01	6.70E-15	55.73	0.01	0.02	0.65
RA	PsO	rs6930468	G	A	NA	0.09	0.01	5.50E-11	69.18	0.02	0.02	0.16
RA	PsO	rs706778	T	C	NA	0.09	0.01	1.50E-10	38.70	0.05	0.01	0.00
RA	PsO	rs71508903	T	C	NA	0.16	0.02	2.30E-20	83.79	-0.01	0.02	0.63
RA	PsO	rs73013527	T	C	NA	-0.09	0.02	9.80E-11	32.48	0.01	0.02	0.60
RA	PsO	rs73081554	T	C	NA	0.17	0.03	4.60E-08	31.69	0.00	0.03	0.97

RA	PsO	rs76153210	T	C	NA	0.17	0.03	2.20E-09	48.04	-0.04	0.07	0.61
RA	PsO	rs7731626	A	G	NA	-0.19	0.02	7.30E-24	105.79	-0.05	0.02	0.00
RA	PsO	rs7752903	G	T	NA	0.33	0.03	2.70E-26	126.89	-0.27	0.06	0.00
RA	PsO	rs7754520	T	C	NA	-0.46	0.02	9.00E-67	378.94	-0.07	0.03	0.02
RA	PsO	rs8026898	A	G	NA	0.15	0.01	6.50E-19	129.80	-0.01	0.02	0.38
RA	PsO	rs8032939	C	T	NA	0.12	0.02	4.80E-16	44.37	0.01	0.02	0.57
RA	PsO	rs8083786	G	A	NA	0.13	0.02	1.00E-15	52.18	0.06	0.02	0.00
RA	PsO	rs9277411	T	C	NA	-0.29	0.01	1.50E-85	459.03	-0.07	0.02	0.00
RA	PsO	rs9348832	A	G	NA	0.25	0.03	5.20E-19	68.80	0.01	0.04	0.75
RA	PsO	rs947474	A	G	NA	0.10	0.02	1.50E-08	33.38	0.01	0.02	0.77
RA	PsO	rs9603616	T	C	NA	-0.11	0.02	4.60E-12	39.66	-0.07	0.02	0.00
RA	PsO	rs9747973	T	C	NA	-0.09	0.01	1.90E-12	72.29	0.00	0.02	0.77
Uveitis	PsO	rs117327462	A	C	0.0153977	0.92	0.19	7.31E-07	12625.93	-0.06	0.13	0.64
Uveitis	PsO	rs11907497	C	T	0.203639	-0.17	0.03	7.24E-07	4309.06	-0.02	0.02	0.22
Uveitis	PsO	rs144614916	C	A	0.0241206	0.79	0.12	2.85E-10	14442.40	0.06	0.07	0.39
Uveitis	PsO	rs145639523	T	C	0.0258446	0.68	0.07	5.52E-22	11536.13	0.06	0.03	0.11
Uveitis	PsO	rs148498636	G	A	0.0472465	0.72	0.15	2.22E-06	23521.20	0.02	0.03	0.66
Uveitis	PsO	rs17315135	T	C	0.0216639	0.70	0.15	2.81E-06	10173.31	-0.01	0.10	0.89
Uveitis	PsO	rs189949127	T	G	0.008049	0.93	0.20	4.44E-06	6744.28	-0.10	0.09	0.26
Uveitis	PsO	rs201276681	A	C	0.00842703	2.07	0.43	1.70E-06	36938.21	0.03	0.05	0.59

Uveitis	PsO	rs2223591	A	G	0.0379502	0.36	0.07	2.47E-07	4480.27	0.11	0.04	0.00
Uveitis	PsO	rs28366149	A	G	0.239279	0.33	0.03	1.67E-21	19496.75	-0.03	0.02	0.04
Uveitis	PsO	rs28432918	A	G	0.249808	-0.18	0.03	3.55E-09	5996.51	-0.04	0.02	0.02
Uveitis	PsO	rs28892580	T	G	0.0673654	-0.29	0.05	1.34E-07	5070.29	-0.09	0.03	0.00
Uveitis	PsO	rs55679315	G	A	0.119026	0.22	0.05	3.38E-06	4993.30	-0.01	0.03	0.71
Uveitis	PsO	rs6138056	G	A	0.724646	0.14	0.03	3.55E-06	3622.64	-0.02	0.02	0.14
Uveitis	PsO	rs62256214	A	C	0.0169765	0.46	0.10	2.20E-06	3359.75	0.11	0.05	0.02
Uveitis	PsO	rs62264596	C	A	0.0493943	0.38	0.08	3.72E-06	6672.30	0.02	0.05	0.71
Uveitis	PsO	rs685031	A	G	0.698084	-0.35	0.03	2.20E-23	25704.84	0.07	0.02	0.00
Uveitis	PsO	rs73208917	T	C	0.0201047	0.59	0.13	3.94E-06	6719.79	0.03	0.07	0.65
Uveitis	PsO	rs79211491	C	A	0.083634	0.22	0.04	6.93E-08	3488.60	-0.01	0.02	0.61
CD	PsO	rs10114470	C	T	NA	0.17	0.02	1.76E-21	90.84	0.04	0.02	0.03
CD	PsO	rs1012636	T	G	NA	0.13	0.02	7.01E-11	42.51	0.07	0.02	0.00
CD	PsO	rs10492862	A	C	NA	0.11	0.02	1.26E-09	36.75	-0.03	0.02	0.05
CD	PsO	rs10822050	C	T	NA	0.18	0.02	2.35E-29	127.18	0.01	0.02	0.37
CD	PsO	rs10884966	A	G	NA	0.11	0.02	4.13E-11	43.74	0.02	0.02	0.13
CD	PsO	rs11236797	A	C	NA	0.18	0.02	8.51E-28	119.50	0.05	0.02	0.00
CD	PsO	rs112856973	C	T	NA	-0.16	0.02	3.61E-11	44.00	-0.03	0.02	0.15
CD	PsO	rs11378157	AG	A	NA	-0.14	0.02	9.29E-14	55.32	-0.03	0.02	0.18
CD	PsO	rs114802258	T	C	NA	-0.22	0.04	5.11E-09	34.18	-0.06	0.03	0.05

CD	PsO	rs1148246	T	C	NA	-0.13	0.02	2.09E-15	62.76	0.00	0.02	0.99
CD	PsO	rs11683692	C	T	NA	-0.21	0.04	1.75E-08	31.83	0.02	0.03	0.45
CD	PsO	rs1250573	A	G	NA	-0.15	0.02	1.92E-17	72.29	-0.07	0.02	0.00
CD	PsO	rs12936409	T	C	NA	0.14	0.02	4.31E-19	79.43	0.00	0.01	0.78
CD	PsO	rs1297264	G	A	NA	-0.18	0.02	1.59E-27	117.78	-0.03	0.02	0.06
CD	PsO	rs1321859	T	C	NA	-0.10	0.02	1.18E-09	37.19	0.00	0.02	0.91
CD	PsO	rs1373904	G	A	NA	0.14	0.02	9.11E-14	55.65	0.01	0.02	0.65
CD	PsO	rs144309607	T	C	NA	-0.37	0.05	2.69E-15	62.37	-0.22	0.05	0.00
CD	PsO	rs145568234	G	T	NA	0.86	0.06	4.31E-42	184.66	-0.08	0.12	0.54
CD	PsO	rs181826	A	C	NA	0.12	0.02	3.24E-12	48.41	0.03	0.02	0.05
CD	PsO	rs194746	T	C	NA	0.10	0.02	1.24E-09	36.67	0.03	0.01	0.05
CD	PsO	rs2002695	G	A	NA	-0.13	0.02	8.31E-12	46.80	0.00	0.02	0.87
CD	PsO	rs2076756	G	A	NA	0.39	0.02	1.80E-108	489.55	0.01	0.02	0.54
CD	PsO	rs212409	A	G	NA	-0.11	0.02	1.49E-11	45.77	-0.04	0.02	0.01
CD	PsO	rs2143178	C	T	NA	-0.21	0.02	6.84E-21	87.58	0.00	0.02	0.92
CD	PsO	rs2284553	G	A	NA	0.13	0.02	1.14E-14	59.90	-0.01	0.02	0.60
CD	PsO	rs28999107	T	G	NA	0.11	0.02	1.06E-09	37.02	-0.02	0.02	0.17
CD	PsO	rs2948542	G	A	NA	0.10	0.02	5.15E-10	38.85	-0.01	0.02	0.34
CD	PsO	rs3122605	A	G	NA	-0.17	0.02	1.24E-14	59.29	-0.06	0.02	0.00
CD	PsO	rs34004493	G	A	NA	0.13	0.02	2.00E-12	49.39	0.06	0.02	0.00

CD	PsO	rs34635748	T	C	NA	0.48	0.05	1.95E-21	90.47	0.00	0.05	0.98
CD	PsO	rs3761158	A	G	NA	-0.11	0.02	2.65E-11	44.28	-0.01	0.02	0.33
CD	PsO	rs3816234	A	G	NA	0.27	0.02	1.51E-62	278.59	0.00	0.02	0.98
CD	PsO	rs4077515	T	C	NA	0.18	0.02	3.14E-30	130.12	0.04	0.02	0.01
CD	PsO	rs4343432	G	A	NA	0.11	0.02	3.50E-12	48.05	0.05	0.02	0.00
CD	PsO	rs4380956	A	G	NA	0.13	0.02	1.15E-15	64.00	0.04	0.02	0.01
CD	PsO	rs4807570	A	G	NA	0.18	0.02	6.03E-21	88.04	0.01	0.02	0.59
CD	PsO	rs56116661	T	C	NA	-0.13	0.02	5.67E-10	38.30	-0.07	0.02	0.00
CD	PsO	rs61839660	T	C	NA	0.15	0.03	1.98E-08	31.63	0.09	0.04	0.01
CD	PsO	rs62126620	A	G	NA	0.14	0.02	8.61E-13	51.32	0.00	0.02	0.84
CD	PsO	rs62324212	A	C	NA	0.11	0.02	8.02E-11	42.29	0.03	0.02	0.07
CD	PsO	rs6451494	C	T	NA	0.26	0.02	8.26E-56	246.25	0.03	0.02	0.02
CD	PsO	rs6579807	T	C	NA	0.20	0.02	3.44E-16	66.71	-0.03	0.02	0.25
CD	PsO	rs6584282	G	A	NA	-0.17	0.02	3.44E-25	107.38	0.03	0.01	0.08
CD	PsO	rs6679677	A	C	NA	-0.23	0.03	1.77E-15	63.27	0.03	0.02	0.20
CD	PsO	rs6704109	T	C	NA	0.17	0.02	5.10E-22	93.26	0.02	0.02	0.29
CD	PsO	rs6740847	G	A	NA	-0.10	0.02	9.72E-11	41.72	0.01	0.02	0.39
CD	PsO	rs6873866	C	T	NA	-0.13	0.02	1.35E-15	64.19	-0.01	0.02	0.65
CD	PsO	rs714910	C	A	NA	-0.15	0.02	2.49E-17	71.54	0.00	0.02	0.82
CD	PsO	rs72798422	C	T	NA	0.55	0.04	6.05E-47	206.91	0.08	0.04	0.06

CD	PsO	rs744166	G	A	NA	-0.11	0.02	1.80E-12	49.69	0.02	0.02	0.18
CD	PsO	rs7517847	G	T	NA	-0.34	0.02	5.84E-97	436.41	0.00	0.02	0.81
CD	PsO	rs7608697	C	A	NA	0.12	0.02	4.03E-14	56.85	0.06	0.02	0.00
CD	PsO	rs77566919	A	G	NA	-0.11	0.02	4.13E-09	34.65	-0.03	0.02	0.05
CD	PsO	rs79832570	C	T	NA	0.22	0.03	8.90E-11	42.17	0.02	0.03	0.48
CD	PsO	rs80262450	A	G	NA	0.23	0.02	1.34E-20	86.39	0.05	0.02	0.03
CD	PsO	rs9656588	C	T	NA	0.12	0.02	8.73E-12	46.76	0.01	0.02	0.40
UC	PsO	rs10272963	T	C	NA	-0.15	0.02	4.11E-21	89.30	0.00	0.01	0.74
UC	PsO	rs10408351	A	G	NA	0.15	0.02	2.92E-14	57.58	0.04	0.02	0.06
UC	PsO	rs10761659	G	A	NA	0.13	0.02	1.33E-15	63.60	0.03	0.01	0.02
UC	PsO	rs10817678	A	G	NA	0.13	0.02	4.42E-15	61.39	0.03	0.02	0.10
UC	PsO	rs1131095	C	T	NA	0.16	0.02	2.18E-21	89.91	-0.02	0.02	0.15
UC	PsO	rs113846785	CG	C	NA	-0.16	0.02	1.15E-12	50.48	0.00	0.02	0.89
UC	PsO	rs113986290	T	C	NA	-0.31	0.05	7.59E-09	33.34	-0.05	0.06	0.35
UC	PsO	rs11651246	G	T	NA	0.15	0.02	2.01E-11	45.05	0.03	0.02	0.14
UC	PsO	rs12825700	A	G	NA	0.19	0.02	7.33E-32	137.66	-0.02	0.02	0.24
UC	PsO	rs12936409	T	C	NA	0.14	0.02	5.62E-18	74.63	0.00	0.01	0.78
UC	PsO	rs1317209	A	G	NA	0.18	0.02	2.90E-19	80.20	-0.05	0.02	0.01
UC	PsO	rs1359946	A	G	NA	0.16	0.02	6.58E-15	60.48	0.00	0.02	0.98
UC	PsO	rs137845	G	A	NA	0.10	0.02	1.50E-10	40.94	-0.02	0.02	0.18

UC	PsO	rs16940186	C	T	NA	0.14	0.02	2.18E-10	40.21	-0.01	0.02	0.51
UC	PsO	rs1736161	A	G	NA	-0.12	0.02	2.22E-14	58.08	-0.03	0.02	0.05
UC	PsO	rs17656349	T	C	NA	0.09	0.02	1.54E-08	32.04	-0.02	0.02	0.11
UC	PsO	rs17715902	A	G	NA	0.10	0.02	4.62E-09	34.43	0.02	0.02	0.21
UC	PsO	rs2045241	A	G	NA	-0.11	0.02	2.83E-10	39.56	-0.01	0.02	0.57
UC	PsO	rs2212434	T	C	NA	0.13	0.02	2.80E-15	62.00	0.05	0.02	0.00
UC	PsO	rs2836881	T	G	NA	-0.22	0.02	1.11E-32	142.06	-0.02	0.02	0.23
UC	PsO	rs3024493	A	C	NA	0.21	0.02	7.46E-24	100.95	0.07	0.02	0.00
UC	PsO	rs3734851	A	G	NA	0.50	0.06	6.58E-18	74.27	-0.20	0.07	0.00
UC	PsO	rs3812565	C	T	NA	0.13	0.02	6.50E-17	69.62	0.04	0.02	0.02
UC	PsO	rs3820330	A	C	NA	-0.16	0.02	3.91E-19	79.49	0.00	0.02	0.95
UC	PsO	rs4676408	A	G	NA	0.14	0.02	1.19E-17	73.63	-0.01	0.02	0.38
UC	PsO	rs4728142	A	G	NA	0.10	0.02	3.23E-10	39.66	0.04	0.02	0.01
UC	PsO	rs4993442	T	G	NA	-0.10	0.02	3.54E-08	30.46	-0.02	0.02	0.13
UC	PsO	rs55905347	A	G	NA	0.11	0.02	2.09E-10	40.31	0.00	0.02	0.98
UC	PsO	rs56062135	T	C	NA	0.11	0.02	4.66E-09	34.32	0.00	0.02	0.90
UC	PsO	rs6017342	C	A	NA	0.19	0.02	3.95E-30	130.76	0.01	0.02	0.40
UC	PsO	rs6062496	A	G	NA	0.14	0.02	8.97E-17	69.51	0.01	0.02	0.55
UC	PsO	rs62180181	T	C	NA	0.12	0.02	8.08E-13	51.40	0.01	0.02	0.64
UC	PsO	rs6889364	A	G	NA	0.13	0.02	7.87E-09	33.42	0.05	0.02	0.01

UC	PsO	rs6933404	C	T	NA	0.15	0.02	2.69E-15	62.47	-0.05	0.02	0.01
UC	PsO	rs72704802	T	C	NA	-0.12	0.02	2.89E-09	35.25	-0.04	0.02	0.07
UC	PsO	rs7523335	A	G	NA	-0.14	0.02	3.42E-11	43.75	0.03	0.02	0.07
UC	PsO	rs7554511	A	C	NA	-0.14	0.02	4.27E-16	66.17	0.00	0.02	0.83
UC	PsO	rs7608697	C	A	NA	0.16	0.02	3.03E-23	98.39	0.06	0.02	0.00
UC	PsO	rs78064630	A	G	NA	0.18	0.03	1.08E-08	32.61	-0.14	0.03	0.00
UC	PsO	rs79051659	A	G	NA	0.16	0.03	1.30E-09	36.96	0.06	0.03	0.03
UC	PsO	rs7911117	G	T	NA	-0.13	0.02	1.84E-08	31.53	0.07	0.02	0.00
UC	PsO	rs7911680	C	A	NA	-0.15	0.02	6.71E-22	91.99	0.02	0.01	0.10
UC	PsO	rs798506	C	T	NA	-0.12	0.02	1.47E-11	45.39	-0.01	0.02	0.70
UC	PsO	rs9611131	C	T	NA	-0.15	0.02	5.11E-11	43.31	-0.01	0.02	0.76
UC	PsO	rs989960	T	C	NA	-0.12	0.02	3.28E-14	57.57	-0.01	0.01	0.36
Vitiligo	PsO	rs10200159	C	T	0.0358	0.18	0.05	3.73E-19	98.10	-0.01	0.04	0.82
Vitiligo	PsO	rs1031034	A	C	0.7137	-0.07	0.03	3.43E-08	77.75	0.01	0.02	0.51
Vitiligo	PsO	rs1043101	G	A	0.3787	0.09	0.02	5.26E-18	169.01	0.00	0.02	0.91
Vitiligo	PsO	rs10986311	C	T	0.3419	0.06	0.02	1.01E-08	73.51	0.05	0.02	0.00
Vitiligo	PsO	rs11021232	C	T	0.1978	0.13	0.03	2.10E-23	228.11	-0.03	0.02	0.20
Vitiligo	PsO	rs1126809	A	G	0.2525	-0.17	0.03	1.16E-43	511.29	0.01	0.02	0.77
Vitiligo	PsO	rs117744081	G	A	0.9692	0.26	0.06	8.72E-26	186.11	0.18	0.04	0.00
Vitiligo	PsO	rs12203592	T	C	0.8837	-0.10	0.04	2.95E-10	95.56	-0.01	0.04	0.81

Vitiligo	PsO	rs12421615	A	G	0.3539	-0.06	0.03	4.81E-08	74.17	-0.06	0.02	0.00
Vitiligo	PsO	rs12771452	A	G	0.2525	-0.09	0.03	4.43E-12	124.47	-0.03	0.02	0.07
Vitiligo	PsO	rs13076312	T	C	0.5189	0.12	0.02	1.61E-30	323.65	0.03	0.01	0.03
Vitiligo	PsO	rs13136820	C	T	0.336	0.07	0.03	3.60E-11	102.29	0.00	0.02	0.79
Vitiligo	PsO	rs1635168	A	C	0.8946	0.14	0.04	8.78E-14	156.59	-0.01	0.07	0.92
Vitiligo	PsO	rs16843742	C	T	0.2247	-0.08	0.03	1.02E-09	101.22	-0.05	0.02	0.00
Vitiligo	PsO	rs2247314	C	T	0.3757	-0.10	0.03	1.72E-18	218.69	-0.02	0.02	0.14
Vitiligo	PsO	rs229527	A	C	0.5775	0.12	0.02	1.14E-30	316.27	0.00	0.02	0.83
Vitiligo	PsO	rs2304206	A	G	0.7406	-0.08	0.03	2.36E-09	97.73	-0.02	0.02	0.32
Vitiligo	PsO	rs231725	A	G	0.3171	0.07	0.03	1.49E-10	99.28	-0.02	0.01	0.16
Vitiligo	PsO	rs34346645	A	C	0.4354	-0.10	0.03	7.99E-19	205.33	-0.03	0.02	0.08
Vitiligo	PsO	rs35860234	G	T	0.2734	0.07	0.03	4.76E-09	81.92	0.03	0.02	0.04
Vitiligo	PsO	rs41342147	A	G	0.8847	-0.10	0.04	3.70E-09	84.97	0.06	0.03	0.05
Vitiligo	PsO	rs4268748	C	T	0.2276	-0.15	0.03	2.88E-33	347.02	-0.01	0.02	0.44
Vitiligo	PsO	rs4308124	C	T	0.4284	0.06	0.02	3.96E-09	80.01	-0.02	0.02	0.24
Vitiligo	PsO	rs4807000	A	G	0.4046	0.08	0.02	1.94E-14	146.64	0.01	0.02	0.51
Vitiligo	PsO	rs561079	C	T	0.3728	0.06	0.02	3.76E-08	67.13	0.00	0.02	0.96
Vitiligo	PsO	rs6012953	G	A	0.5239	0.06	0.02	9.47E-09	81.50	0.03	0.02	0.09
Vitiligo	PsO	rs706779	C	T	0.5189	-0.11	0.02	7.20E-27	286.59	-0.03	0.02	0.03
Vitiligo	PsO	rs71508903	T	C	0.7803	0.07	0.03	6.93E-09	78.56	-0.01	0.02	0.63

Vitiligo	PsO	rs72928038	A	G	0.1799	0.10	0.03	1.00E-14	141.18	-0.01	0.02	0.64
Vitiligo	PsO	rs78037977	G	A	0.8817	0.12	0.03	6.74E-17	134.65	0.06	0.02	0.01
Vitiligo	PsO	rs78521699	G	A	0.8738	-0.10	0.04	2.54E-08	102.55	-0.01	0.02	0.63
Vitiligo	PsO	rs8083511	C	A	0.7873	0.08	0.03	2.81E-10	101.83	0.02	0.02	0.25
Vitiligo	PsO	rs8192917	C	T	0.7525	0.10	0.03	8.91E-16	155.39	-0.01	0.02	0.59
BP	PsO	rs1394944	A	C	0.0421821	0.59	0.13	4.60E-06	10815.23	-0.01	0.04	0.81
BP	PsO	rs2395306	T	A	0.290652	0.35	0.06	4.58E-08	20005.04	-0.04	0.02	0.01
BP	PsO	rs76965871	A	G	0.0112079	1.05	0.21	6.08E-07	9472.05	0.04	0.07	0.56
BP	PsO	rs77062257	T	A	0.0740825	0.91	0.09	1.77E-25	48145.79	-0.10	0.03	0.00
BP	PsO	rs9270891	G	T	0.605266	0.44	0.06	4.98E-12	38629.01	-0.05	0.02	0.00
AS	PsO	rs1041926	A	G	NA	-0.07	0.01	1.55E-10	40.96	0.20	0.05	0.00
AS	PsO	rs11065898	T	C	NA	0.03	0.00	4.71E-08	29.83	-0.03	0.02	0.16
AS	PsO	rs11190133	T	C	NA	-0.03	0.00	4.84E-14	56.79	0.04	0.02	0.02
AS	PsO	rs1128905	C	T	NA	-0.02	0.00	6.95E-09	33.55	0.03	0.01	0.04
AS	PsO	rs11624293	C	T	NA	0.04	0.01	1.49E-10	41.04	0.02	0.03	0.57
AS	PsO	rs1250550	A	C	NA	-0.03	0.00	1.46E-09	36.58	-0.07	0.02	0.00
AS	PsO	rs12615545	C	T	NA	0.03	0.00	1.03E-09	37.26	0.01	0.01	0.43
AS	PsO	rs1801274	G	A	NA	0.03	0.00	1.35E-09	36.74	0.00	0.01	0.78
AS	PsO	rs1860545	A	G	NA	-0.03	0.00	2.78E-10	39.82	0.01	0.02	0.54
AS	PsO	rs2517655	T	C	NA	0.09	0.00	3.47E-80	359.55	0.06	0.02	0.00

AS	PsO	rs2531875	T	G	NA	-0.03	0.00	1.22E-10	41.43	0.00	0.02	0.80
AS	PsO	rs27529	G	A	NA	-0.06	0.00	3.28E-47	208.25	-0.03	0.02	0.03
AS	PsO	rs2836883	A	G	NA	-0.04	0.00	6.46E-17	69.83	-0.02	0.02	0.23
AS	PsO	rs35164067	A	G	NA	-0.03	0.00	3.43E-10	39.41	-0.06	0.02	0.00
AS	PsO	rs4129267	T	C	NA	-0.03	0.00	3.32E-13	53.00	-0.05	0.02	0.00
AS	PsO	rs41299637	G	T	NA	-0.04	0.00	1.81E-15	63.25	0.00	0.02	0.81
AS	PsO	rs4676410	A	G	NA	0.03	0.00	9.90E-09	32.86	0.01	0.02	0.67
AS	PsO	rs7191548	C	T	NA	0.02	0.00	5.51E-09	34.00	0.00	0.02	0.82
AS	PsO	rs9901869	A	G	NA	0.03	0.00	6.04E-15	60.88	0.00	0.01	0.79
HT	PsO	rs11611029	T	C	0.393049	-0.10	0.02	1.16E-08	1740.06	-0.01	0.02	0.33
HT	PsO	rs11889341	T	C	0.249062	0.12	0.02	6.64E-11	2307.30	0.01	0.02	0.66
HT	PsO	rs2110451	A	G	0.375548	0.10	0.02	3.33E-08	1856.89	0.02	0.02	0.22
HT	PsO	rs34636506	G	A	0.373849	-0.13	0.02	1.92E-14	3384.49	0.00	0.02	0.80
HT	PsO	rs6679677	A	C	0.10821	0.33	0.02	5.37E-44	8363.06	0.03	0.02	0.20
HT	PsO	rs7030280	T	C	0.747314	0.21	0.02	6.44E-31	6387.03	0.00	0.02	0.76
HT	PsO	rs73192661	T	C	0.428715	-0.10	0.02	1.45E-09	2010.66	-0.03	0.02	0.04
HT	PsO	rs9271365	G	T	0.442657	0.25	0.02	1.44E-39	12913.72	-0.05	0.02	0.00

Abbreviations: PsO, psoriasis; SLE, Systemic lupus erythematosus; MS, multiple sclerosis; RA, Rheumatoid arthritis; CD, Crohn's disease; UC, Ulcerative colitis; BP, Bullous pemphigoid; AS, Ankylosing spondylitis; HT, Hashimoto thyroiditis; AIDs, Autoimmune diseases.

Table S11. F-statistics for all MR analyses.

Exposure	Outcomes	F-statistics*
PsO	SLE	2702.49
	MS	19648.45
	CD	2832.61
	UC	2044.02
	Uveitis	4281.15
	BP	80.28
	HT	4245.47
	RA	2430.11
	AS	2165.14
	Vitiligo	2480.66
PsA	SLE	9552.15
	MS	4073.36
	CD	5164.37
	UC	9492.59
	Uveitis	6389.79
	BP	1586.78
	HT	12356.27
	RA	5153.26
	AS	4182.11
	Vitiligo	4797.54
SLE	PsO	135.50
MS		43.84
CD		99.42
UC		65.23
Uveitis		18376.55
BP		33944.50
HT		5287.04
RA		98.46
AS		72.88

Vitiligo		175.46
SLE		121.49
MS		43.25
CD		91.45
UC		67.72
Uveitis		18376.55
BP	PsA	21799.41
HT		304.56
RA		62.04
AS		69.19
Vitiligo		175.46

Abbreviations: PsO, psoriasis; PsA, psoriatic arthritis, SLE, Systemic lupus erythematosus; MS, multiple sclerosis; RA, Rheumatoid arthritis; CD, Crohn's disease; UC, Ulcerative colitis; BP, Bullous pemphigoid; AS, Ankylosing spondylitis; HT, Hashimoto thyroiditis; AIDs, Autoimmune diseases; MR, Mendelian randomization
