# Supplementary material for: Causal associations between both psoriasis and psoriatic arthritis and multiple autoimmune diseases: a bidirectional two-sample Mendelian randomization study
Source: Front Immunol. 2024 Jul 25;15:1422626. doi: 10.3389/fimmu.2024.1422626 (PMC11306030; doi:10.3389/fimmu.2024.1422626)
Supplement: Supplementary file 2 [file DataSheet_2.pdf]

STROBE-MR checklist of recommended items to address in reports of Mendelian randomization studies[1 2]

Item No.	Section	Checklist item	Relevant text from manuscript
1	**TITLE and ABSTRACT**	Indicate Mendelian randomization (MR) as the study's design in the title and/or the abstract if that is a main purpose of the study	"Causal Associations between Both Psoriasis and Psoriatic Arthritis and Multiple Autoimmune Diseases: A Bidirectional Two-Sample Mendelian Randomization Study"
	**INTRODUCTION**		
2	**Background**	Explain the scientific background and rationale for the reported study. What is the exposure? Is a potential causal relationship between exposure and outcome plausible? Justify why MR is a helpful method to address the study question	"Numerous observational studies have found that psoriasis (PsO) and psoriatic arthritis (PsA) could be comorbid with autoimmune diseases (AIDs). Specifically, research indicated that the risk of comorbidities with AIDs in PsO patients was five times greater than that of the general population9. Additionally, a South Korean study found a significantly higher risk of rheumatoid arthritis (RA), ankylosing spondylitis (AS), Crohn's disease (CD), and systemic lupus erythematosus (SLE) in 321,354 PsO patients compared to 321,354 healthy individuals10. Sardu C et al. selected 25,885 individuals from Sardinia, Italy, to assess the prevalence of 12 AIDs, finding that PsO/PsA ranked second in prevalence within the population. Additionally, a retrospective study demonstrated a direct correlation between comorbidity incidence and disease severity in PsO patients, with PsA patients experiencing a significantly higher incidence of comorbidities than those with PsO. Although observational studies are widely used for the preliminary etiological investigations, their susceptibility to confounding factors and challenges in determining the temporal sequence of causality make these relationships unclear.Therefore, we performed a Mendelian randomization analysis to delve deeper into the causal relationships between PsO, PsA, and AIDs."

3	**Objectives**	State specific objectives clearly, including pre-specified causal hypotheses (if any). State that MR is a method that, under specific assumptions, intends to estimate causal effects	"We performed a Mendelian randomization (MR) analysis to delve deeper into the causal relationships between PsO, PsA, and AIDs such as SLE, multiple sclerosis (MS), CD, ulcerative colitis (UC), uveitis, bullous pemphigoid (BP), Hashimoto's thyroiditis (HT), RA, AS, and vitiligo."
	**METHODS**		
4	**Study design and data sources**	Present key elements of the study design early in the article. Consider including a table listing sources of data for all phases of the study. For each data source contributing to the analysis, describe the following:	Figure 1 provides a detailed illustration of the three fundamental assumptions of Mendelian randomization studies, the GWAS data information for various phenotypes, and the specific research steps involved. These steps include the selection criteria for instrumental variables, statistical methods for Mendelian randomization, and sensitivity analysis.
	a)	Setting: Describe the study design and the underlying population, if possible. Describe the setting, locations, and relevant dates, including periods of recruitment, exposure, follow-up, and data collection, when available.	"We conducted a bidirectional two-sample MR analysis based on the Genome-Wide Association Studies (GWAS) database to explore the causal links between PsO, PsA, and AIDs. This MR study followed three fundamental assumptions: (1) the correlation assumption: the IVs should be strongly correlated with the exposure; (2) the exclusivity assumption: the IVs have no direct relation to the outcome; (3) the independence assumption: the IVs shouldn't be related to any confounder that affects the exposure-outcome relationship.<br><br>The GWAS databases for PsO (9,267 cases and 364,071 controls), PsA (3,186 cases and 240,862 controls), and BP (507 cases, 375,767 controls) all originated from the FinnGen Consortium, which is a large-scale biomedical research project based in Finland that aims to uncover new biomarkers and therapeutic targets by analyzing the genetic information and health data of Finnish participants. To address the sample overlap among PsO, PsA, and BP, additional GWAS databases were selected for PsO and PsA. Specifically, the GWAS data for PsO (15,967

		cases and 28,194 controls) were derived from a cross-ethnic investigation conducted by Stuart PE et al., which compared PsO susceptibility between South Asians and Europeans17. The GWAS data for PsA (5,065 cases and 21,286 controls) were sourced from a study by Soomro M et al.18, which developed a database to examine genetic markers differentiating PsA from PsO without arthritis. GWAS data for SLE (5,201 cases and 9,066 controls), MS (47,429 cases and 68,374 controls), CD (12,194 cases and 28,072 controls), UC (12,366 cases and 33,609 controls), uveitis (2,616 cases and 478,126 controls), AS (9,069 cases and 13,578 controls), RA (14,361 cases and 33,609 controls), and HT (15,654 cases and 379,986 controls) were sourced from the IEU database (https://gwas.mrcieu.ac.uk). Moreover, GWAS data for vitiligo (4,680 cases and 39,586 controls) were sourced from the most extensive meta-analyses available19. The comprehensive GWAS data information is summarized in Table S1.”
	b) Participants: Give the eligibility criteria, and the sources and methods of selection of participants. Report the sample size, and whether any power or sample size calculations were carried out prior to the main analysis	“The GWAS databases for PsO (9,267 cases and 364,071 controls), PsA (3,186 cases and 240,862 controls), and BP (507 cases, 375,767 controls) all originated from the FinnGen Consortium, which is a large-scale biomedical research project based in Finland that aims to uncover new biomarkers and therapeutic targets by analyzing the genetic information and health data of Finnish participants. To address the sample overlap among PsO, PsA, and BP, additional GWAS databases were selected for PsO and PsA. Specifically, the GWAS data for PsO (15,967 cases and 28,194 controls) were derived from a cross-ethnic investigation conducted by Stuart PE et al., which compared PsO susceptibility between South Asians and Europeans17. The GWAS data for PsA (5,065 cases and 21,286 controls) were sourced from a study by Soomro M et al.18, which developed a database to examine genetic markers

		differentiating PsA from PsO without arthritis. GWAS data for SLE (5,201 cases and 9,066 controls), MS (47,429 cases and 68,374 controls), CD (12,194 cases and 28,072 controls), UC (12,366 cases and 33,609 controls), uveitis (2,616 cases and 478,126 controls), AS (9,069 cases and 13,578 controls), RA (14,361 cases and 33,609 controls), and HT (15,654 cases and 379,986 controls) were sourced from the IEU database (https://gwas.mrcieu.ac.uk). Moreover, GWAS data for vitiligo (4,680 cases and 39,586 controls) were sourced from the most extensive meta-analyses available19. The comprehensive GWAS data information is summarized in Table S1."<br>Table S1 in the supplementary material presents detailed information on the traits involved in this study.
c)	Describe measurement, quality control and selection of genetic variants	"Selecting appropriate IVs requires adherence to six steps. Firstly, a single nucleotide polymorphism (SNP) must demonstrate a strong correlation with the exposure (p < 5×10-8). Secondly, we set a linkage disequilibrium (LD) threshold (r2) of 0.001 and a 10 Mb clumping window to guarantee the independence of each SNP20. Should the number of selected SNPs be insufficient, the thresholds for p and r2 can be adjusted to a minimum of p < 5×10-6 and r2 < 0.01, respectively. Thirdly, we exclude the SNPs that exhibit a strong correlation with the outcome variable (poutcome < pexposure) . Fourthly, harmonize the data between SNPexposure and SNPoutcome to ensure alignment of allelic directions and compatibility for analysis. Fifthly, confounders are eliminated through the Phenoscanner website to mitigate potential pleiotropic effects21. Finally, the association strength between IVs and the exposure is measured using F-statistics, computed as F=R2/(1-R2)×(N-K-1)/K, with R2=2×MAF×(1-MAF)×β2."<br>Table S1 in the supplementary material presents detailed information on the traits

				involved in this study.
		d)	For each exposure, outcome, and other relevant variables, describe methods of assessment and diagnostic criteria for diseases	This manuscript mentions the definitions of the diseases in the introduction, methods, and discussion sections.
		e)	Provide details of ethics committee approval and participant informed consent, if relevant	"All data utilized herein were derived from the public genome-wide association studies, thus obviating the need for further ethical consent."
5	**Assumptions**		Explicitly state the three core IV assumptions for the main analysis (relevance, independence and exclusion restriction) as well assumptions for any additional or sensitivity analysis	"We conducted a bidirectional two-sample MR analysis based on the Genome-Wide Association Studies (GWAS) database to explore the causal links between PsO, PsA, and AIDs. This MR study followed three fundamental assumptions: (1) the correlation assumption: the IVs should be strongly correlated with the exposure; (2) the exclusivity assumption: the IVs have no direct relation to the outcome; (3) the independence assumption: the IVs shouldn't be related to any confounder that affects the exposure-outcome relationship. MR analysis primarily employs Inverse Variance Weighted (IVW) to determine the existence of causality, while MR Egger (ME), Weighted Median (WM), and Maximum Likelihood (ML) serve as supplementary analytical approaches. IVW is characterized by its disregard for the intercept term, fitting the data using the inverse of the outcome variance as weights. Unlike IVW, ME incorporates the intercept term within its regression model, also applying the inverse of outcome variance as weights for fitting. WM is the median of the distribution function derived from sorting all SNP effect values by their weights. It delivers a reliable causal effect estimate, despite having up to 50% invalid IVs. ML, grounded in principles of probability theory, estimates unknown parameters by identifying model parameters that maximize the likelihood of the observed data.<br><br>We employed Cochran's Q test, leave-one-out analysis, and the MR-Egger intercept for sensitivity analysis. The objectives of the

				sensitivity analyses are threefold: first, to evaluate the dependability of the MR analysis outcomes; second, to explore potential biases, such as genetic pleiotropy and data heterogeneity; and third, to determine whether a specific SNP significantly affects the outcome. We employed Cochran's Q test to assess the extent of heterogeneity. When significant heterogeneity occurs (P < 0.05), we employ MR radial analysis to remove outliers and correct the estimates to verify the reliability of the findings. Leave-one-out analysis determines the combined effect of the remaining SNPs by sequentially excluding each SNP, with all error lines located consistently on one side of zero, suggesting dependable outcomes. Furthermore, MR studies should primarily focus on the horizontal pleiotropy to avoid genetic variants influencing the outcome through exposure. If the intercept of the MR-Egger regression is significantly different from zero (P < 0.05), it indicates the presence of horizontal pleiotropy, and we use MR radial analysis to correct the estimates by excluding outliers.”
6	**Statistical methods: main analysis**		Describe statistical methods and statistics used	
		a)	Describe how quantitative variables were handled in the analyses (i.e., scale, units, model)	“MR analysis primarily employs Inverse Variance Weighted (IVW) to determine the existence of causality, while MR Egger (ME), Weighted Median (WM), and Maximum Likelihood (ML) serve as supplementary analytical approaches. IVW is characterized by its disregard for the intercept term, fitting the data using the inverse of the outcome variance as weights25. Unlike IVW, ME incorporates the intercept term within its regression model, also applying the inverse of outcome variance as weights for fitting26. WM is the median of the distribution function derived from sorting all SNP effect values by their weights. It delivers a reliable causal effect estimate, despite having up to 50%

		invalid IVs27. ML, grounded in principles of probability theory, estimates unknown parameters by identifying model parameters that maximize the likelihood of the observed data28. If P < 0.05 for IVW, a causal link between exposure and outcome is inferred when all five conditions are met simultaneously: (1) at least one other statistical method yields a p-value < 0.05; (2) the odds ratios (OR) from IVW, WM, and ML consistently indicate the same direction of effect; (3) there is no significant evidence of horizontal pleiotropy (P > 0.05); (4) all error lines of the leave-one-out analysis plot are all on the same side of zero; (5) these conditions still hold after adjusting for heterogeneity.”
b)	Describe how genetic variants were handled in the analyses and, if applicable, how their weights were selected	“Selecting appropriate IVs requires adherence to six steps. Firstly, a single nucleotide polymorphism (SNP) must demonstrate a strong correlation with the exposure (p < 5×10-8). Secondly, we set a linkage disequilibrium (LD) threshold (r2) of 0.001 and a 10 Mb clumping window to guarantee the independence of each SNP20. Should the number of selected SNPs be insufficient, the thresholds for p and r2 can be adjusted to a minimum of p < 5×10-6 and r2 < 0.01, respectively. Thirdly, we exclude the SNPs that exhibit a strong correlation with the outcome variable (poutcome < pexposure) . Fourthly, harmonize the data between SNPexposure and SNPoutcome to ensure alignment of allelic directions and compatibility for analysis. Fifthly, confounders are eliminated through the Phenoscanner website to mitigate potential pleiotropic effects21. Finally, the association strength between IVs and the exposure is measured using F-statistics, computed as F=R2/(1-R2)×(N-K-1)/K, with R2=2×MAF×(1-MAF)×β2. In the absence of MAF values, R2 is determined by R2=β2/(β2+SE2×N)22-24, where R2 indicates the variance in exposure explained by the IVs, N represents the total sample size of the exposure GWAS, MAF denotes

				the frequency of minor allele, K refers to the number of IVs, and β signifies the SNP's effect size on exposure. If the F-statistic exceeds 10, it implies that MR analyses are unlikely to be biased by weak IVs. Conversely, SNPs with F-statistics below this threshold should be excluded.”
		c)	Describe the MR estimator (e.g. two-stage least squares, Wald ratio) and related statistics. Detail the included covariates and, in case of two-sample MR, whether the same covariate set was used for adjustment in the two samples	/
		d)	Explain how missing data were addressed	This requirement is not very applicable to MR studies.
		e)	If applicable, indicate how multiple testing was addressed	This study is not a multivariable MR study.
7	**Assessment of assumptions**		Describe any methods or prior knowledge used to assess the assumptions or justify their validity	This study used F-statistics to estimate statistical power:<br>“Finally, the association strength between IVs and the exposure is measured using F-statistics, computed as F=R2/(1-R2)×(N-K-1)/K, with R2=2×MAF×(1-MAF)×β2. In the absence of MAF values, R2 is determined byR2=β2/(β2+SE2×N)22-24, where R2 indicates the variance in exposure explained by the IVs, N represents the total sample size of the exposure GWAS, MAF denotes the frequency of minor allele, K refers to the number of IVs, and β signifies the SNP's effect size on exposure. If the F-statistic exceeds 10, it implies that MR analyses are unlikely to be biased by weak IVs. Conversely, SNPs with F-statistics below this threshold should be excluded.”
8	**Sensitivity analyses and additional analyses**		Describe any sensitivity analyses or additional analyses performed (e.g. comparison of effect estimates from different approaches, independent replication, bias analytic techniques, validation of instruments, simulations)	**“We employed Cochran's Q test, leave-one-out analysis, and the MR-Egger intercept for sensitivity analysis.** The objectives of the sensitivity analyses are threefold: first, to evaluate the dependability of the MR analysis outcomes; second, to explore potential biases, such as genetic pleiotropy and data heterogeneity; and third, to determine whether a specific SNP

				significantly affects the outcome. We employed Cochran's Q test to assess the extent of heterogeneity. **When significant heterogeneity occurs (P < 0.05), we employ MR radial analysis to remove outliers and correct the estimates to verify the reliability of the findings.** Leave-one-out analysis determines the combined effect of the remaining SNPs by sequentially excluding each SNP, with all error lines located consistently on one side of zero, suggesting dependable outcomes. Furthermore, MR studies should primarily focus on the horizontal pleiotropy to avoid genetic variants influencing the outcome through exposure. **If the intercept of the MR-Egger regression is significantly different from zero (P < 0.05), it indicates the presence of horizontal pleiotropy, and we use MR radial analysis to correct the estimates by excluding outliers.**"
9	**Software and pre-registration**			
		a)	Name statistical software and package(s), including version and settings used	"This study conducted MR analysis using R software (version 4.2.3), utilizing the R packages TwoSampleMR and RadialMR."
		b)	State whether the study protocol and details were pre-registered (as well as when and where)	This study did not register a research protocol.
	**RESULTS**			
10	**Descriptive data**			
		a)	Report the numbers of individuals at each stage of included studies and reasons for exclusion. Consider use of a flow diagram	The number of participants in the sample is provided in the Methods section of this paper; consequently, it is not reiterated in the Results section.
		b)	Report summary statistics for phenotypic exposure(s), outcome(s), and other relevant variables (e.g. means, SDs, proportions)	Tables S7-S10 present a detailed summary of the summary statistics for phenotypic exposures and outcomes.

		c)	If the data sources include meta-analyses of previous studies, provide the assessments of heterogeneity across these studies	/
		d)	For two-sample MR:<br>i. Provide justification of the similarity of the genetic variant-exposure associations between the exposure and outcome samples<br>ii. Provide information on the number of individuals who overlap between the exposure and outcome studies	i. All the sample populations in this study originate from Europe, thus exhibiting minimal ethnic heterogeneity.<br>ii. The exposure and outcome populations investigated in this study are sourced from different databases, thereby avoiding potential biases caused by sample overlap.
11	**Main results**			
		a)	Report the associations between genetic variant and exposure, and between genetic variant and outcome, preferably on an interpretable scale	This article reports the content of this entry in Tables S7-S10 of the "Supplementary Material", including the number of instrumental variable SNPs, specific information about the SNPs, the association strength between the exposure and instrumental variables, and the association strength between the outcome and instrumental variables.
		b)	Report MR estimates of the relationship between exposure and outcome, and the measures of uncertainty from the MR analysis, on an interpretable scale, such as odds ratio or relative risk per SD difference	"When PsO and PsA were considered as outcomes, genetically predicted CD [IVW odds ratio (ORIVW), 1.11; 95% confidence interval (CI), 1.06-1.17; P = 8.40E-06], vitiligo (ORIVW, 1.16; 95% CI, 1.05-1.28; P = 2.45E-03) were risk factors for PsO, while BP may reduce the incidence of PsO (ORIVW, 0.91; 95% CI, 0.87-0.96; P = 1.26E-04). Furthermore, no causal link was found between the remaining AIDs and PsO. CD (ORIVW, 1.07; 95% CI, 1.02-1.12; P = 0.01), HT (ORIVW, 1.23; 95% CI, 1.08-1.40; P = 1.43E-03), RA (ORIVW, 1.11; 95% CI, 1.02-1.21; P = 2.05E-02), AS (ORIVW, 2.18; 95% CI, 1.46-3.27; P = 1.55E-04), SLE (ORIVW, 1.04; 95% CI, 1.01-1.08; P = 1.07E-02) and vitiligo (ORIVW, 1.27; 95% CI, 1.14-1.42; P = 2.67E-05) were risk factors for PsA. There were no causal associations found between the remaining AIDs and PsO/PsA (Figures 1, 2; Tables S3, S4)."

				Detailed effect sizes and precision for the associations between exposures and outcomes are presented in Figures 1 and 2, as well as in Supplementary Material Figures S1, S2; Tables S2-S5.
		c)	If relevant, consider translating estimates of relative risk into absolute risk for a meaningful time period	This study continues to use relative risk for causal assessment.
		d)	Consider plots to visualize results (e.g. forest plot, scatterplot of associations between genetic variants and outcome versus between genetic variants and exposure)	Forest plots and scatter plots can be found in Figures 2 and 3 of the manuscript, as well as in supplementary material Figures S1-S19.
12	**Assessment of assumptions**			
		a)	Report the assessment of the validity of the assumptions	Firstly, this article specifies six steps for selecting instrumental variables with robust statistical power in the 'Instrumental Variables Selection' section. Additionally, the statistical power of these instrumental variables is detailed in Tables S7-S11 of the Supplementary Material, with results presented as F-statistics.
		b)	Report any additional statistics (e.g., assessments of heterogeneity across genetic variants, such as I^2, Q statistic or E-value)	This article presents detailed I^2 values in Tables S2-S6 of the Supplementary Material to evaluate the heterogeneity of causal estimates from instrumental variables.
13	**Sensitivity analyses and additional analyses**			
		a)	Report any sensitivity analyses to assess the robustness of the main results to violations of the assumptions	"In addition to the previously mentioned pleiotropy between CD and PsA, we also found pleiotropy between SLE and PsO (P = 0.038). Therefore, we utilized the MR-Radial to remove five outlier SNPs—rs389884, rs4274624, rs4388254, rs58688157, and rs58721818—which effectively mitigated pleiotropy. In studies with causal

				relationships, significant heterogeneity was observed when CD and vitiligo affected PsO. Therefore, we used MR-Radial to reduce heterogeneity and found that the positive results remained stable (Figure S19, Table S6). Furthermore, in a leave-one-out analysis, excluding each SNP individually did not result in significant variation in the estimated causal effects (Figure S3-S18). In summary, our study findings are deemed reliable and valid."
		b)	Report results from other sensitivity analyses or additional analyses	Leave-one-out plots are detailed in Figures S3-S19 of the Supplementary Material.
		c)	Report any assessment of direction of causal relationship (e.g., bidirectional MR)	This study is a bidirectional two-sample Mendelian Randomization analysis. The results section not only provides a detailed report on the evaluation of the bidirectional MR analysis but also organizes the relevant detailed data in the supplementary materials.
		d)	When relevant, report and compare with estimates from non-MR analyses	We conducted a comprehensive discussion of the MR analysis results and their correlation with observational studies and disease mechanisms in the discussion section.
		e)	Consider additional plots to visualize results (e.g., leave-one-out analyses)	Leave-one-out plots and scatter plots are detailed in Figures S3-S19 of the Supplementary Material.
	**DISCUSSION**			
14	**Key results**		Summarize key results with reference to study objectives	Genetic prediction results indicate that BP might lower the risk of developing PsO, while CD and vitiligo may increase it. Additionally, certain AIDs including CD, AS, RA, HT, vitiligo, and SLE are more likely to induce PsA.
15	**Limitations**		Discuss limitations of the study, taking into account the validity of the IV assumptions, other sources of potential bias, and imprecision. Discuss both	"However, our study also presents several limitations. Firstly, the lack of pertinent GWAS data prevents us from exploring the

			direction and magnitude of any potential bias and any efforts to address them	causal links between different subtypes. Secondly, the GWAS database we used primarily targeted European populations, potentially limiting its applicability to other ethnicities. Thirdly, the small GWAS dataset for BP in our study necessitates larger future datasets for validating our findings. Fourthly, although using multiple methods to control confounders, potential horizontal pleiotropy may still exist. Lastly, this study may have overlooked other AIDs are causally linked to PsO and PsA.”
16	**Interpretation**			
		a)	Meaning: Give a cautious overall interpretation of results in the context of their limitations and in comparison with other studies	We comprehensively analyzed the connections between the MR analysis results and observational studies, as well as the pathogenesis of the disease, providing a reasonable explanation for the MR findings.
		b)	Mechanism: Discuss underlying biological mechanisms that could drive a potential causal relationship between the investigated exposure and the outcome, and whether the gene-environment equivalence assumption is reasonable. Use causal language carefully, clarifying that IV estimates may provide causal effects only under certain assumptions	We comprehensively analyzed the connections between the MR analysis results and observational studies, as well as the biomechanism, providing a reasonable explanation for the MR findings.
		c)	Clinical relevance: Discuss whether the results have clinical or public policy relevance, and to what extent they inform effect sizes of possible interventions	“Our study has three important implications: (1) It provides evidence of potential causal relationships between certain AIDs and both PsO and PsA, suggesting that increased surveillance of these conditions should be considered in clinical practice; (2) Given that AIDs are more likely to precipitate the occurrence of PsA and most PsA patients initially present only with skin lesions, it can be inferred that patients with PsO and comorbid AIDs are more likely to develop PsA. It indicates that AIDs could be potential risk factors for progressing from PsO to PsA, underscoring the importance of early monitoring for PsA; (3) This study also underscores the critical importance of establishing GWAS database for PsO subtypes, which is vital for future etiological research into PsO. “

17	**Generalizability**	Discuss the generalizability of the study results (a) to other populations, (b) across other exposure periods/timings, and (c) across other levels of exposure	"The GWAS database we used primarily targeted European populations, potentially limiting its applicability to other ethnicities."
	**OTHER INFORMATION**		
18	**Funding**	Describe sources of funding and the role of funders in the present study and, if applicable, sources of funding for the databases and original study or studies on which the present study is based	"This study was supported by the Research Project "Study on the Effects of Subcutaneous Injection and Intravenous Infusion of Adipose-Derived Mesenchymal Stem Cells on Psoriasis Mouse Animal Models(2023KTSCX026)" and "Guangdong Province's Third Batch of Famous Traditional Chinese Medicine Practitioner Inheritance Projects - Ruiqiang Fan Guangdong Famous Traditional Chinese Medicine Inheritance Studio."
19	**Data and data sharing**	Provide the data used to perform all analyses or report where and how the data can be accessed, and reference these sources in the article. Provide the statistical code needed to reproduce the results in the article, or report whether the code is publicly accessible and if so, where	"The detailed data and GWAS data sources referenced in this study are available in the article/supplementary materials. Should you require further data, please contact the author directly."
20	**Conflicts of Interest**	All authors should declare all potential conflicts of interest	"The authors declare that the research was conducted in the absence of any commercial or financial relationships that could be construed as a potential conflict of interest."

This checklist is copyrighted by the Equator Network under the Creative Commons Attribution 3.0 Unported (CC BY 3.0) license.

1. Skrivankova VW, Richmond RC, Woolf BAR, Yarmolinsky J, Davies NM, Swanson SA, et al. Strengthening the Reporting of Observational Studies in Epidemiology using Mendelian Randomization (STROBE-MR) Statement. JAMA. 2021;under review.

2. Skrivankova VW, Richmond RC, Woolf BAR, Davies NM, Swanson SA, VanderWeele TJ, et al. Strengthening the Reporting of Observational Studies in Epidemiology using Mendelian Randomisation (STROBE-MR): Explanation and Elaboration. BMJ. 2021;375:n2233.
